# Reindeer health and supplementary feeding practices – a survey among herders in Norway and Sweden

**DOI:** 10.1186/s13028-026-00868-3

**Published:** 2026-09-22

**Authors:** Stine Bull-Aurbakken, Ester Malmström, Jerome Baron, Javier Sánchez Romano, Karin Wallin Philippot, Heidi Rautiainen, Anna Omazic, Ingebjørg Helena Nymo, Morten Tryland

**Affiliations:** 1 Department of Forestry and Wildlife Management, University of Inland Norway, Anne Evenstad vei 80, Koppang, 2480 Norway; 2 Institute of Global Health, Heidelberg University Hospital, Heidelberg, Germany; 3 Institute of Zoology, Zoological Society of London, Regent’s Park, London, NW8 7LS UK; 4 Department of Medical Biology, Faculty of Health Sciences, UiT The Arctic University of 12 Norway, Hansine Hansens veg 18, Tromsø, 9091 Norway; 5 Department of Clinical Sciences, Swedish University of Agricultural Sciences (SLU), Uppsala, Sweden; 6 Department of Animal Health and Antimicrobial Strategies, Swedish Veterinary Agency, Uppsala, 75189 Sweden; 7 Department of Applied Animal Science and Welfare, Swedish University of Agricultural Sciences, Uppsala, Sweden; 8 Department of Chemistry, Environment and Feed Hygiene, Swedish Veterinary Agency, Uppsala, 75189 Sweden; 9 The Norwegian Veterinary Institute, Holtvegen 66, Tromsø, 9016 Norway; 10 Department of Arctic and Marine Biology, Faculty of Biosciences, Fisheries and Economics, UiT The Arctic University of Norway, Hansine Hansens veg 18, Tromsø, 9019 Norway

**Keywords:** Climate change, Diarrhoea, Enclosure, Infectious disease, One health, Pasture, Reindeer husbandry, Veterinary medicine, Wet belly.

## Abstract

**Background:**

The well-being, health and nutrition of reindeer (*Rangifer tarandus tarandus*) and the practice of reindeer husbandry in Fennoscandia are strongly affected by climate change and the loss and fragmentation of pastures. Supplementary feeding has become increasingly common to cope with limited pasture availability, especially to avoid starvation during winters with ice-locked pastures and challenging snow conditions. Increased reliance on feeding shifts traditional reindeer husbandry towards a more farm-like system and raises concerns about changes in reindeer foraging behaviour, increased stress and disease transmission. In this study, a questionnaire was conducted among reindeer herders in Norway and Sweden, to review the extent of supplementary feeding and to explore links between feeding strategies and observations of disease in reindeer. The study also evaluated factors affecting access to and use of veterinary services.

**Results:**

A total of 76 respondents, 33 from Norway and 43 from Sweden, participated. Most (87%) had applied supplementary feeding between 2015 and 2021, mainly on free-range pasture, although we observed a non-significant increasing trend in feeding in enclosures during this period. Detailed analysis of the challenging winter of 2019–2020 showed that it was most common to feed a combination of roughage (hay or silage) and pellets, and feeding typically lasted longer than one month. The most frequently reported health issues included parasites, diarrhoea, abortion, and wet belly. Reports of diarrhoea, wet belly, and necrobacillosis were significantly more common in Sweden than in Norway. Multiple correspondence analysis and hierarchical clustering analysis demonstrated an association between feeding in enclosures and diarrhoea. Veterinary services were used by 34% of the respondents for necropsies and 55% for clinical disease management, although many noted barriers such as cost and limited veterinary knowledge and expertise in reindeer health.

**Conclusions:**

Supplementary feeding is widespread in reindeer husbandry in Norway and Sweden. Feeding in enclosures was associated with a higher incidence of diarrhoea. Continued competence building for good management decisions and veterinary services is essential for sustainable reindeer husbandry. An integrated One Health approach is key for the promotion of reindeer health and welfare and in preparedness for transmissible diseases among reindeer, domestic livestock and wildlife.

**Supplementary Information:**

The online version contains supplementary material available at https://doi.org/10.1186/s13028-026-00868-3.

## Background

Reindeer husbandry in Norway and Sweden is a northern pastoralist livelihood based on the use of natural pastures as the food source for reindeer [1], which rely on functional landscapes and flexibility in pasture use. These systems are challenged by ecosystem changes caused by anthropogenic installations and activities along with climate change, leading to reduced availability and accessibility to pastures [2–4]. In 2024, the spring populations of semidomesticated reindeer counted approximately 215,000 in Norway [5] and 250,000 in Sweden [6].

The increased frequency and severity of critical periods with ice-locked pastures resulting from freeze‒thaw cycles and rain-on-snow events are major challenges directly related to rapid climate change in arctic and sub-arctic regions [7–9]. Another pressing issue is that reindeer pastures and migratory routes are fragmented and made unavailable by anthropogenic installations and activities such as roads, wind farms, powerlines, mining and forestry, along with increased tourism and recreational activity and the presence of predators [8, 10–12]. The loss of flexibility in pasture use resulting from these factors is a pronounced challenge for reindeer herders [13–15].

To mitigate constraints on pasture availability, there has been a marked increase in the use of supplementary feeding in reindeer husbandry in Norway and Sweden in recent decades, whereas it has been common in Finland since the 1980s [16–18]. The advantages of feeding include improved body condition and survival through harsh winters [19] and utility as a herding tool [20]. There are, however, concerns about the effects of supplementary feeding on reindeer’s natural foraging behaviour [21] and how altered strategies might lead to a loss of traditional knowledge about herd dynamics and pastoral landscapes [3].

Supplementary feeding can also introduce stressors, including abrupt dietary changes, increased animal density during corralling or enclosure feeding, and greater human disturbance [16]. Severe or prolonged stress may suppress immune function and increase susceptibility to infectious diseases, which could increase risk of disease outbreaks from opportunistic pathogens [22, 23] and exacerbation of the severity of clinical conditions [24, 25]. Feeding conditions may also impact meat quality and food safety [26].

Under natural grazing conditions, clinical diseases related to digestive disturbances and gastrointestinal parasites are rarely observed in reindeer. However, certain reindeer diseases are directly related to the type, quality or quantity of forage, and the transition from natural pasture to feed [16]. As for other ruminants, one common example is ruminal acidosis, which is associated with the intake of large amounts of readily fermentable carbohydrates without being accustomed to it. The resulting drop in rumen pH represents a life-threatening condition, as the absorbed acid may exceed the buffer capacity of the blood [27]. Typical clinical signs of ruminal acidosis are fluid splashing sounds in the rumen (giving rise to the common names "Skvalpmage” in Norwegian and Swedish and “Skulči/duški čoavji (čoalit skuhraidit)” in Northern Sámi, translating to fluid-filled rumen or “rippling belly” in English), along with depression, dehydration and diarrhoea [27]. Another condition seen in reindeer is “wet belly" (from the common name “våt buk” in Norwegian and Swedish and “Čáhcečoavji/Njuoskaoaččát čoavjjevuolli” in Northern Sámi), characterized by wet fur on part of the ventral trunk and legs. The pathogenesis of this condition is unknown but appears to be strictly associated with feeding [16, 28, 29]. Wet belly has been reported as relatively common in Sweden and Finland [30, 31].

Furthermore, increased parasite transmission may become a concern when reindeer are kept under conditions with higher animal density and increased faecal contamination, such as feeding sites and enclosures [32, 33]. The clinical relevance of several gastrointestinal parasites commonly found in reindeer remains poorly described. However, some parasite species have demonstrated pathogenic effects, depending on their virulence and predilection site. For example, *Eimeria* spp., unicellular parasites of the small intestine, have been associated with diarrhoea in reindeer calves, whereas the reindeer abomasal nematode *Ostertagia gruehneri* has been linked to weight loss, inflammation and reduced fecundity (summarized in [34]).

Reduced fecundity in ruminants may in general be attributed to a multitude of conditions, including stress, starvation [35], and several infectious diseases that may be relevant in reindeer [29, 34]. Globally, pestivirus infections are a prominent cause of reduced fecundity in livestock. The impact of pestivirus is poorly understood in reindeer, although it is shown to be prevalent in reindeer populations in Norway and Sweden [36, 37].

Several infectious diseases in reindeer have been found to be associated with stress, crowding and unfavourable hygienic conditions. This includes necrobacillosis (*Fusobacterium necrophorum*) [38], contagious ecthyma (parapoxvirus) [39] and pasteurellosis (*Pasteurella multocida*) [40, 41]. Increased transmission and manifestation under such circumstances likely also apply to *Cervidpoxvirus reindeerpox* (CvRPV) (tentative name), a novel virus recently detected in semi-domesticated reindeer [42]. Feeding in enclosures has also been identified as a risk factor for a higher prevalence of infectious keratoconjunctivitis (IKC) [43, 44], a well known disease in reindeer, typically caused by cervid herpesvirus 2 (CvHV2) in an interplay with bacterial pathogens [45, 46].

Assessing the impacts of local management practices on pathogen exposure in combination with those of climate change need to be addressed together with those who have first-hand experience with both the prerequisites, the animals and the applied practices – the reindeer herders. In this study, we analysed data from a comprehensive questionnaire survey among reindeer herders in Norway and Sweden to map the extent of supplementary feeding between 2015 and 2021 and to investigate how feeding strategies may influence disease occurrence in reindeer. In addition, we addressed the use of veterinary support for managing the health and welfare of reindeer.

## Materials and methods

### The questionnaire and included data

A digital questionnaire made in Questback Essentials (v. 38, Questback Sweden Ltd, 2021) covering demographic data, supplementary feeding practices, disease observations, and disease management was generated in Norwegian, Swedish, and Northern Sámi (see Additional files 1–3, respectively). A link to the survey and accompanying information was distributed to the registered contact person or chairperson of each herding district in Norway (*n* = 83) and Sweden (*n* = 51), inviting all herding unit leaders to participate, potentially reaching a total of 1,583 active herding groups (535 siida units in Norway and 1,048 family herding groups in Sweden). The questionnaire was open from April to September 2021. Further details regarding distribution and follow-up of the questionnaire are provided in a previous publication [43].

Supplementary feeding was defined as “supplying feedstuff to a winter herd for more than two weeks” (hereafter referred to as “feeding”). The data included in this study, encompass general demographic information (country, county/geographical unit, and herd size) and feeding practices during the previous six herding years (2015–2021), with particular focus on the winter season of 2019–2020, when pasture conditions were especially challenging. For this season, additional details were collected on feed type (roughage, pellets, or a combination thereof) and feeding duration (< 1 month, 1–3 months, or > 3 months).

**Fig. 1:**
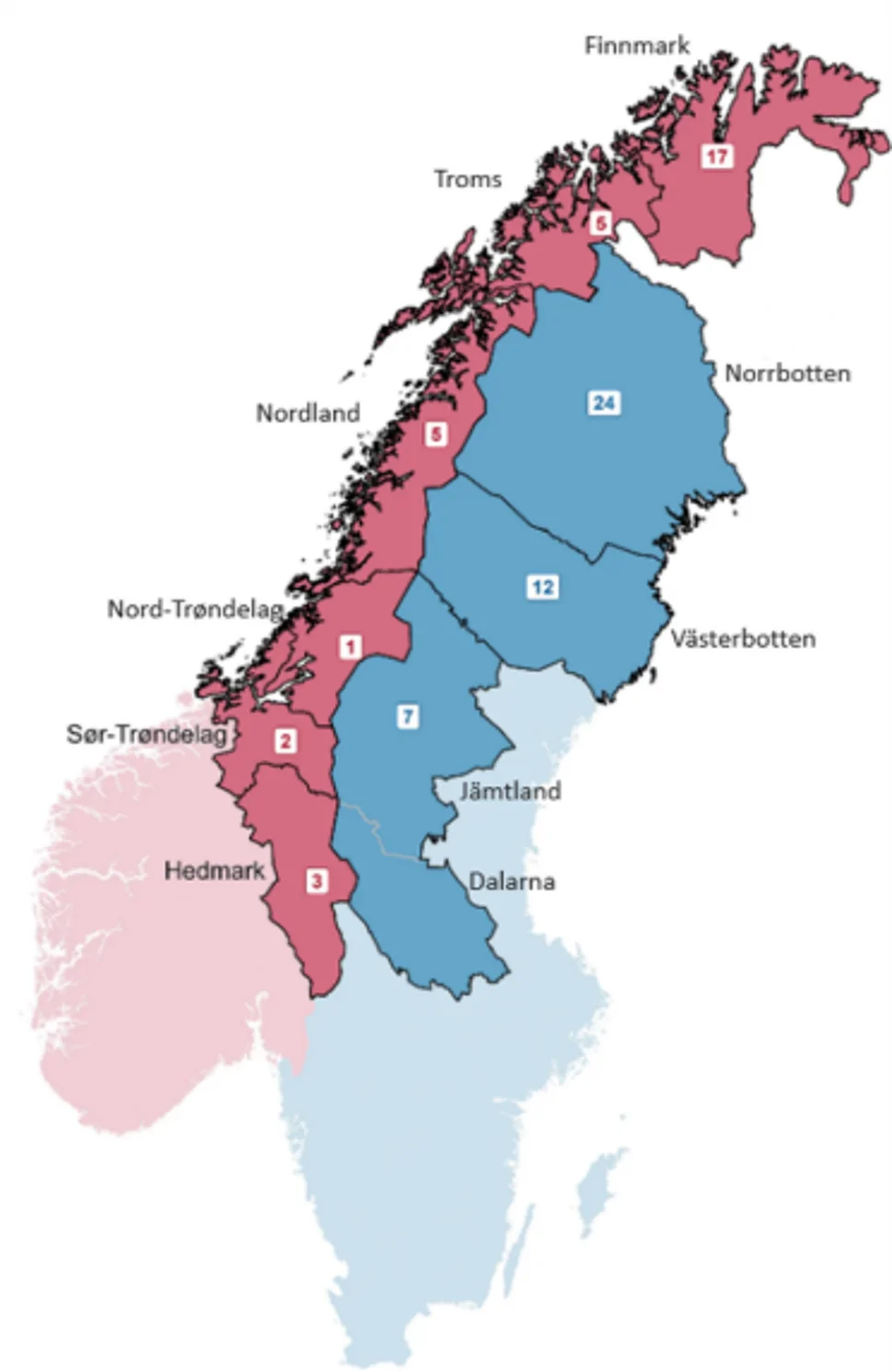
Distribution of respondents (*N* = 76) in a survey among reindeer herders in Norway and Sweden. Norwegian counties are presented as they were named before 2018. Figure adapted from a previous study based on material from the same survey [43]

Reported disease observations from the winter season of 2019–2020 until the end of the survey (September 2021) were also included. These referred to observed signs of the following health issues and diseases: acidosis, diarrhoea, bloating, wet belly, contagious ecthyma (parapoxvirus), necrobacillosis, pneumonia, parasites, brainworm, ataxia, abortion, and unspecified mortality. Data regarding the disease infectious keratoconjunctivitis (IKC) were also collected and have been published in a previous article [43]. Respondents could additionally report and specify other health issues that were not listed in the questionnaire. Information on disease intervention and follow-up, including the use of veterinary assistance, was also included. Due to the low number of respondents in certain herding areas, responses were aggregated into larger geographical units. In Norway, West Finnmark and East Finnmark were combined into one unit; Nordland and Troms into a second; and North Trøndelag and South Trøndelag/Hedmark into a third. In Sweden, Dalarna and Jämtland were combined into one geographical unit (Fig. 1). Herd size was categorized into four groups: <500, 500–999, 1,000–1,999, and ≥ 2,000 animals.

### Statistical analysis

The analysis focused on two main questions: (1) “How were feeding and management practices associated with observation of the listed diseases?” and (2) “Which management practices were associated with requesting veterinary help?”. To address these questions, we combined descriptive, exploratory, and complementary inferential approaches.

### Descriptive analysis

To provide an overview of feeding practices and disease observations, the distribution of responses was summarized in counts and proportions. Between-country differences were evaluated using Fisher’s exact test due to small sample sizes in several categories. Simple linear regression was used to assess the change in numbers of respondents reporting the different feeding practices across the study period.

### Multiple correspondence analysis and hierarchical clustering analysis

To explore relationships across multiple categorical variables, we applied multiple correspondence analysis (MCA) using the FactoMineR package [47]. MCA is suitable for visualizing structural patterns among categorical variables in complex survey datasets [48, 49]. Feeding and demographic variables were included as active (exposure) variables, computing the dimensions of principal components in the model. Disease observations and variables relating to necropsy and veterinary assistance were included as passive (outcome) variables, meaning that they did not influence how herds were grouped, but were instead projected onto the model matrix, to explore their relationships with the identified pattern of principal components. This approach enabled us to evaluate how well the grouping of herds, as determined by their feeding practices and demographic characteristics, corresponded to the observation of diseases and the actions taken in response to it.

Based on the principal components and dimensions from the MCA, hierarchical clustering analysis (HCA) was performed to identify groups or clusters with similar feeding and demographic profiles. The distribution of disease observations was interpreted within these clusters.

A total of 12 exposure variables, encompassing seven binary and five multi-categorical variables related to supplementary feeding and herd demographics were included in the MCA and HCA models. Among the 16 outcome variables, 12 were related to the presence or absence of health conditions and diseases, and four were related to actions taken in response to disease (Table 1).

**Table 1:** Variables included in multivariate analyses of feeding practices and disease observations

Demographics and supplementary feeding (exposure/active variables)		Disease (outcome/passive variables)
Presence or absence variables (*n* = 7)	Multi-categorical variables (*n* = 5)	Presence or absence variables (*n* = 16)
Feeding during the study period (2015–2021) Feeding on pasture 2019–2020 Feeding pellets on pasture 2019–2020 Feeding roughage on pasture 2019–2020 Feeding in enclosure 2019–2020 Feeding pellets in enclosure 2019–2020 Feeding roughage in enclosure 2019–2020	Herd size Country Geographical unit Duration of feeding on pasture Duration of feeding in enclosure	Fluid filled rumen Diarrhoea Bloating Wet belly Contagious ecthyma (Orf) Necrobacillosis Pneumonia Parasites Brainworm Ataxia Abortion Unspecified mortality Requesting veterinary assistance Necropsy performed by any actor Necropsy performed by owner Necropsy performed by veterinarian

The variables were included in multiple correspondence analysis and hierarchical clustering analysis, derived from a questionnaire survey with 76 responding reindeer herders in Norway and Sweden in 2021. The survey covered demography and recent years’ supplementary feeding practices, disease observations, and follow-up actions

### Logistic regression

A logistic regression model with binomial distribution was used to assess the trend of feeding in enclosure between 2015 and 2021, estimating the change in the odds of applying this feeding practice per year.

Based on the exploratory analysis in MCA and HCA, the following hypotheses were formed:

1. Feeding in enclosures was associated with a higher likelihood of observed digestive disturbances (including diarrhoea and wet belly).
2. Feeding type and duration influenced the probability of observing disease.
3. Feeding practices and disease observation differed between countries.

To complement exploratory analyses and address these questions further in an inferential framework, we conducted a series of both univariate and multivariate logistic regression models with binomial distribution.

Associations between observation of disease and feeding practices were analysed, with results reported as odds ratios (OR), 95% confidence intervals (CI), and corresponding P-values, where *P* < 0.05 was considered statistically significant. Full model outputs are provided in Additional file 6.

## Results

In total, 76 reindeer herders completed the questionnaire: 33 from Norway (43%) and 43 from Sweden (57%). Responses were obtained from all regional areas in the Sámi reindeer herding area (Fig. 1). The distribution of respondents by country, district type, and herd size is shown in Table 2.

**Table 2:** Distribution of survey respondents by district type and herd size

Country	Reindeer herding district type			Reindeer herd size			
	Concession	Mountain	Forest	< 500	500- 999	1000- 1999	≥ 2000
Sweden (*n* = 43)	9	10	24	10	11	12	10
Norway (*n* = 33)	–	–	–	13	6	4	10
Total	9	10	24	23	17	16	20

Numbers represent the distribution of respondents (*N* = 76) in a 2021 questionnaire survey among reindeer herders, by district types in Sweden (concession, mountain, and forest) and by herd size in both Norway and Sweden

### Supplementary feeding 2015–2021

Of the 76 respondents, 66 (87%) reported using supplementary feeding (i.e., feeding > 2 weeks) during the period of 2015–2021. A higher proportion of respondents in Sweden (91%) than Norway (82%) had been feeding (Table 3). Feeding on pasture was the most common practice overall, followed by feeding both on pasture and in enclosures. In Norway, feeding on pasture only was the most common practice, followed by a combination of feeding on pasture and feeding in enclosure. In Sweden, a combination of both feeding practices was most common, followed by feeding on pasture only.

**Table 3:** Supplementary feeding practices 2015–2021 reported by reindeer herders in Norway and Sweden (*N* = 76)

Year	No supplementary feeding		Feeding on pasture only		Feeding in enclosure only		Feeding both in enclosure and on pasture		Total feeding in enclosure	Total feeding overall
	Norway Sweden		Norway Sweden		Norway Sweden		Norway Sweden			
2015/2016	6	5	1	7	0	6	3	13	22	47
2016/2017	6	11	14	9	1	5	5	12	23	46
2017/2018	6	7	16	11	0	5	5	13	23	50
2018/2019	5	5	16	11	0	6	6	14	26	53
2019/2020	1	4	17	12	0	4	9	17	30	59
2020/2021	10	6	13	10	0	7	4	16	27	50

The table shows the distribution of survey respondents from Norway (*n*=33) and Sweden (*n*=43), respectively, reporting no supplementary feeding or feeding on pasture, in enclosure, or both, during each reindeer herding year (April 1 – March 31), 2015–2021

**Fig. 2:**
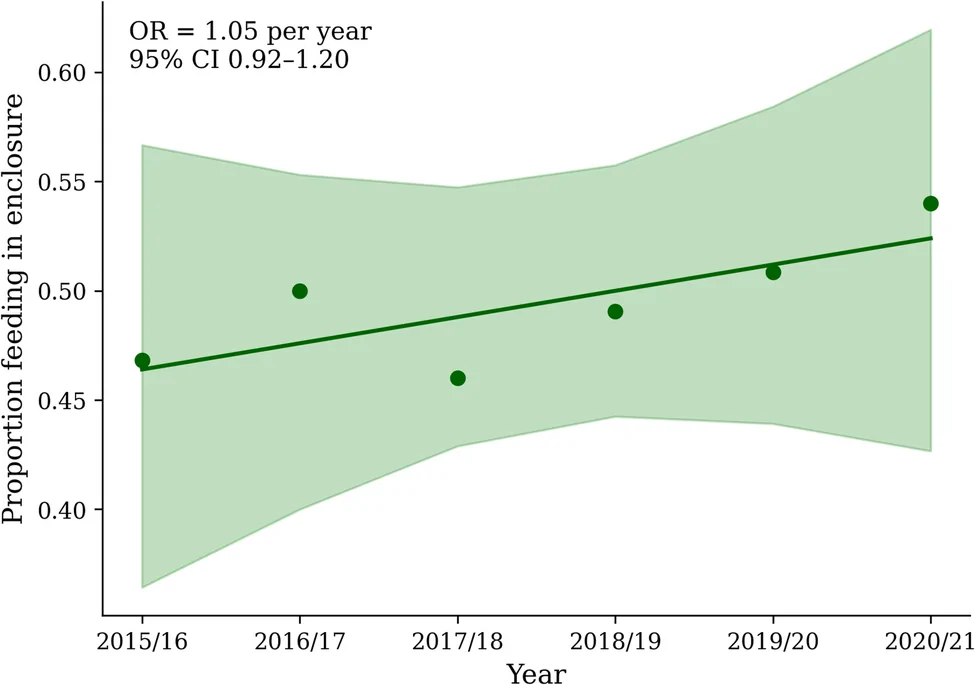
Annual proportion of respondents feeding in an enclosure among reindeer herders reporting supplementary feeding 2015–2021. Points represent the observed proportions, and the dark green line shows the predicted probability from the logistic regression model. The shaded ribbon indicates the 95% confidence interval of the trend. The proportion of respondents feeding in an enclosure showed a non-significant increase across the study period (OR = 1.05 per year, *P* = 0.48)

The number of respondents reporting feeding in enclosure increased across the study period (linear regression: slope =increase of 1.4 per year, *P* = 0.029). However, when accounting for variation in response rate between years with logistic regression, the proportion of respondents feeding in enclosure showed only a modest non-significant increase (OR = 1.05 per year, 95% CI: 0.92–1.20, *P* = 0.48) (Fig. 2.) Still, feeding exclusively in enclosure was the least common practice in both countries (Table 3).

### Supplementary feeding during the winter of 2019–2020

The highest feeding rate occurred in winter 2019–2020, when 59 respondents (78%) reported supplementary feeding. Feeding on pasture only was most common (Norway: 17; Sweden: 12), followed by combined feeding in pasture and enclosures (Norway: 9; Sweden: 17). Four respondents in Sweden and no respondents in Norway reported feeding exclusively in enclosures. Feeding in enclosures was more common among smaller herds (< 300 animals), whereas larger herds were typically fed on pasture.

The most used feed type was a combination of pellets and roughage, reported by 73% of those feeding on pasture and 91% feeding in enclosures. The use of pellets only was less common, followed by roughage only, which was practiced by two respondents (one from each country) when feeding on pasture (Table 4). Feeding usually exceeded one month in both countries. In Sweden, it was more common to feed for longer durations in enclosures, with a significantly larger proportion (*P* < 0.05) feeding > 3 months (52%), than in Norway (11%) (Table 4). Feeding in enclosure was relatively uncommon in Norway, both for feeding calves only (9%) or the entire herd (12%), whereas the practice of feeding calves in enclosures was more common in Sweden (35%). Several herders noted that annual feeding of specific groups, such as calves, weak individuals, and tamed reindeer, was routine practice.

**Table 4:** Feeding practices reported by reindeer herders in Norway and Sweden for the winter of 2019–2020

Feeding practice		Feeding on pasture			Feeding in enclosure		
		Norway n(%)^1^	Sweden n(%)	Total n(%)	Norway n(%)	Sweden n(%)	Total n(%)
Feeding	No	1(3)	10(30)	11(17)	18(54)	6(14)	24(43)
	Yes	26(79)	29(67)	55(83)	2(6)	23(53)	32(57)
Feed type	Roughage only	1(4)	1(3)	2(4)	0	0	0
	Roughage and pellets	17(65)	23(79)	40(73)	8(89)	21(91)	29(91)
	Pellets only	8(31)	5(17)	13(23)	1(11)	2(9)	3(9)
	Total	26(100)	29(100)	55(100)	9(100)	23(100)	32(100)
Duration of feeding	< 1 month	3(12)	3(10)	6(11)	3(33)	4(17)	7(22)
	1–3 months	11(42)	12(41)	23(42)	5(56)	7(30)	12(38)
	> 3 months	12(46)	14(48)	26(47)	1(11)*	12(52)*	13(40)
	Total	26(100)	29(100)	55(100)	9(100)	23(100)	32(100)

^1^n=number of respondents in each category with percentages in brackets. For feeding (yes/no), percentages (%) are given relative to the total number of respondents from Norway (*n*=33) and Sweden (*n*=43), respectively, and the number of respondents in both countries combined (N=76). For feed type and duration of feeding, percentages are given relative to the number of respondents who answered “yes” to feeding on pasture and in enclosure during this herding year, within each country and in total. Significant differences between countries according to Fisher’s exact test (*P*<0.05) are indicated by *

### Observations of disease

Overall, the most reported conditions were parasites (66%), diarrhoea (57%), abortion (36%), wet belly (30%) and fluid-filled rumen (“skvalpmage”), indicating a state of acidosis (26%).

Fisher’s exact tests indicated that diarrhoea, wet belly, and necrobacillosis were reported significantly more often in Sweden than in Norway (Table 5). When asked to specify any other disease observation that was not listed in the questionnaire, pneumonia was the only distinct disease mentioned, as reported by four respondents. Pneumonia is therefore listed as an observed disease in Table 5 and included as an outcome variable in further analysis (Table 1).

**Table 5:** Observation of diseases in reindeer 2019–2021 reported in a survey among herders in Norway and Sweden

Observed disease/condition	Norway *n* (%)^1^	Sweden *n* (%)	Total *n* (%)	Fisher’s exact test: *P*-value
Parasites	19 (58)	27 (63)	46 (61)	0.81
Diarrhoea	11 (33)	32 (74)	43 (57)	< 0.001*
Abortion	16 (49)	27 (63)	43 (57)	0.25
Wet belly	4 (12)	19 (44)	23 (30)	0.003*
Fluid-filled rumen	7 (21)	12 (28)	19 (25)	0.60
Unspecified mortality	8 (24)	10 (23)	18 (24)	> 0.99
Uterine prolapse	8 (24)	8 (19)	16 (21)	0.60
Brainworm	6 (18)	8 (19)	14 (18)	> 0.99
Ataxia	5 (15)	6 (14)	11 (15)	> 0.99
Bloating	2 (6)	5 (12)	7 (9)	0.69
Necrobacillosis	0 (0)	6 (14)	6 (8)	0.03*
Pneumonia	1 (3)	3 (7)	4 (5)	0.63
Contagious ecthyma (Orf)	1 (3)	1 (2)	2 (3)	0.99

^1^n=number of respondents who reported the disease or condition. The percentages (%) are given relative to the total number of respondents from Norway (*n* = 33) and Sweden (*n* = 43), and the number of respondents in both countries combined (*N* = 76). Significant differences between countries according to Fisher’s exact test (*P* < 0.05) are indicated by *. The diseases or disease conditions are explained in further detail in the text

### Multiple correspondence analysis

**Fig. 3:**
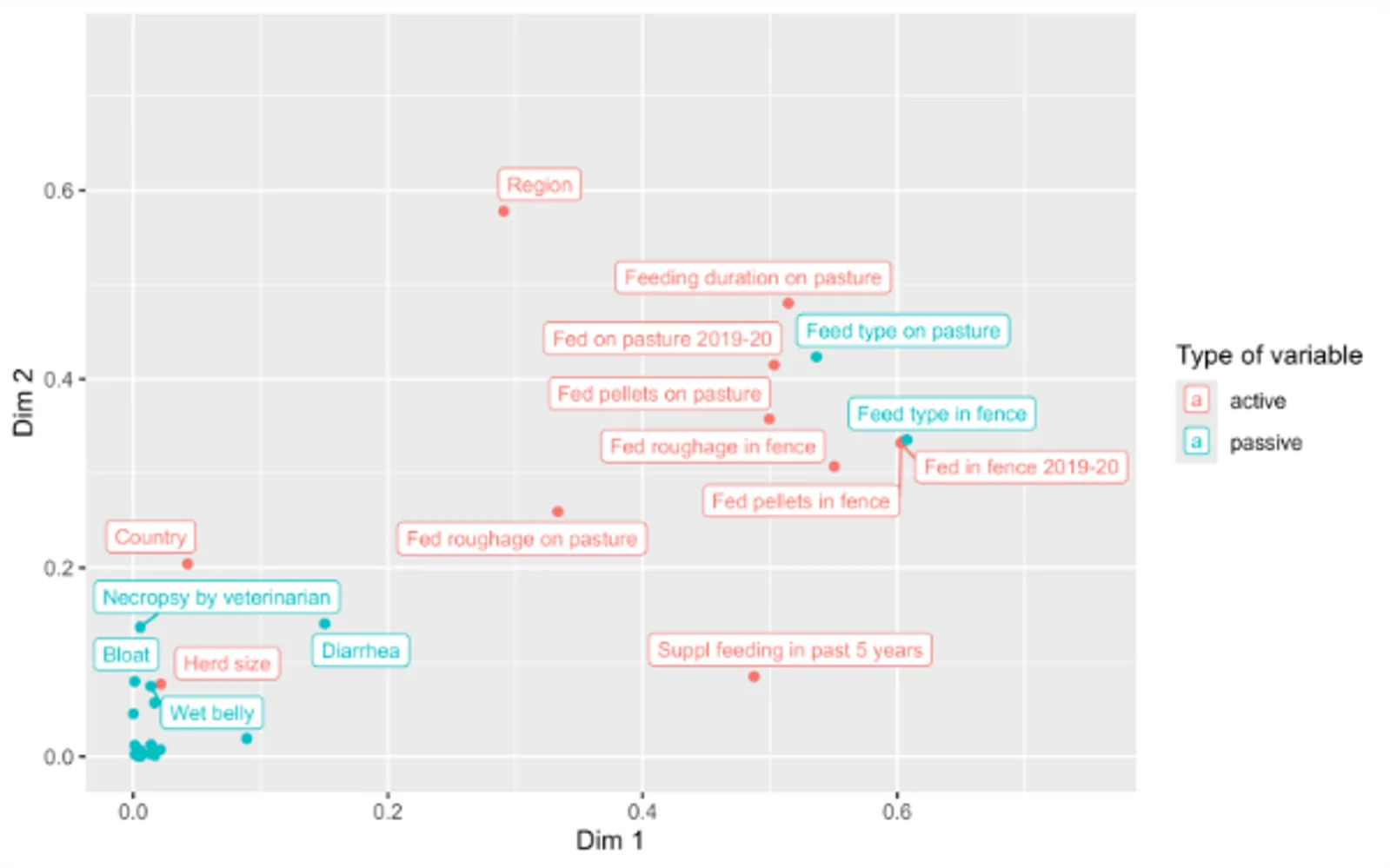
Distribution of R² values for variables in a survey among reindeer herders in Norway and Sweden in 2021. The distribution is shown in two dimensions defined in a multiple correspondence analysis (MCA) model. R² values indicate how well a variable contributes to explaining the patterns or structure observed in the data. R² values close to 1 suggest that the variable explains a large amount of the variation, whereas values close to 0 indicate that the variable has little influence on the observed patterns

The MCA identified two main dimensions explaining 23% and 17% of the variation in the dataset (Fig. 3).

The MCA revealed that feeding practices were the primary contributing factors to the variation among respondents, with all feeding variables, except for feeding roughages on pasture, showing relatively high discriminatory values (greater than 0.4). Demographic variables (country, region and herd size) contributed less to the dimensions.

Among the listed diseases, diarrhoea had the strongest alignment with the feeding-based patterns identified by MCA, as diarrhoea was positioned close to enclosure feeding and pelleted feed along the first dimension, indicating an association between these variables in the dataset (Fig. 3). Other diseases showed weaker or inconsistent patterns. Notably, all the outcome variables (including the observation of diarrhoea) were denoted by low values (below 0.2) in both dimensions, indicating that none of the disease variables had very strong correlation with the patterns modelled from the exposure variables, and were thus not strongly explained by feeding practice.

### Hierarchical clustering analysis

**Fig. 4:**
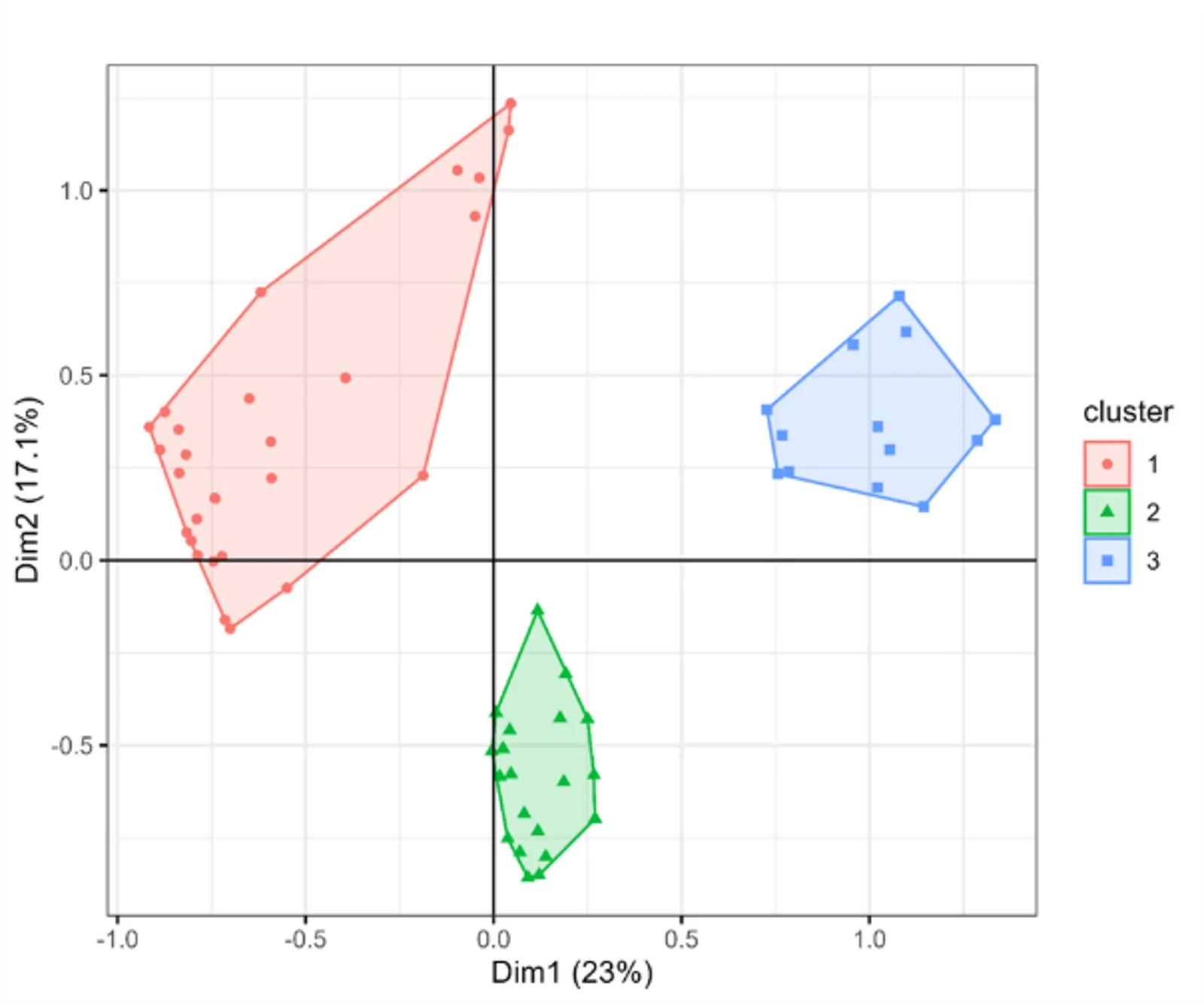
Clustered distribution of responses (*N* = 76) to a survey among reindeer Norway and Sweden. Three clusters were defined by hierarchical clustering analysis (HCA); 1 (red), 2 (green) and 3 (blue) for multiple correspondence analysis (MCA), modelling how exposure variables (demographics and feeding) related to outcome variables (disease observation and veterinary assistance). The percentages for dimensions 1 and 2 represent the proportion of variance in the model explained in the two dimensions

The HCA identified three main clusters (Fig. 4): Cluster 1, predominantly constituted respondents from Västerbotten, Sweden, that frequently reported use of enclosures and feeding a combination of roughage and pellets, higher proportion of reported diarrhoea and were more likely to have necropsies performed by veterinarians. Cluster 2 were predominantly respondents from Finnmark, Norway, that had been feeding mainly on pasture, often > 3 months, almost no reports of diarrhoea and where few necropsies had been conducted. Cluster 3 included respondents from the joint region of Hedmark and North- and South Trøndelag counties in Norway, who had not been feeding during 2015–2021, and exhibited no distinct disease pattern – neither presence nor absence of diseases (Fig. 4). Closer details of the clusters are provided in Table 6.

**Table 6:** Clusters defined by multivariate analysis of responses for the reindeer herding year 2019 − 2021

Cluster	Demographics and feeding (exposure variables)		Disease and necropsy (outcome variables)
**1**	Fed in the last 6 years: yes Fed in enclosure in 2019-20: yes Fed roughage in enclosure: yes Fed pellets in enclosure: yes	Feeding duration in enclosure: > 3 months Feeding duration in enclosure: 1–3 months Feeding duration in enclosure: < 1 months Region: Västerbotten (Sweden)	Diarrhoea: yes Necropsy by veterinarian: yes
**2**	Fed in enclosure in 2019-20: no Fed roughage in enclosure: no Fed pellets in enclosure: no Fed on pasture in 2019-20: yes Fed pellets on pasture: yes	Feeding duration in enclosure: 0 months Feeding duration on pasture: > 3 months Region: Finnmark (Norway)	Diarrhoea: no Necropsy by veterinarian: no
**3**	Fed in the last 5 years: no Fed on pasture in 2019-20: no Fed roughage on pasture: no Fed pellets on pasture: no Fed in enclosure in 2019-20: no Fed roughage in enclosure: no Fed pellets in enclosure: no	Feeding duration on pasture: 0 months Feeding duration in enclosure: 0 months Region: Trøndelag/Hedmark (Norway)	No noticeable relationship to presence or absence of disease

The exposure and outcome variables were arranged in three clusters defined by hierarchical clustering analysis (HCA) and multiple correspondence analysis (MCA) for the reindeer herding year 2019 − 2021 (Fig. 4)

**Fig. 5:**
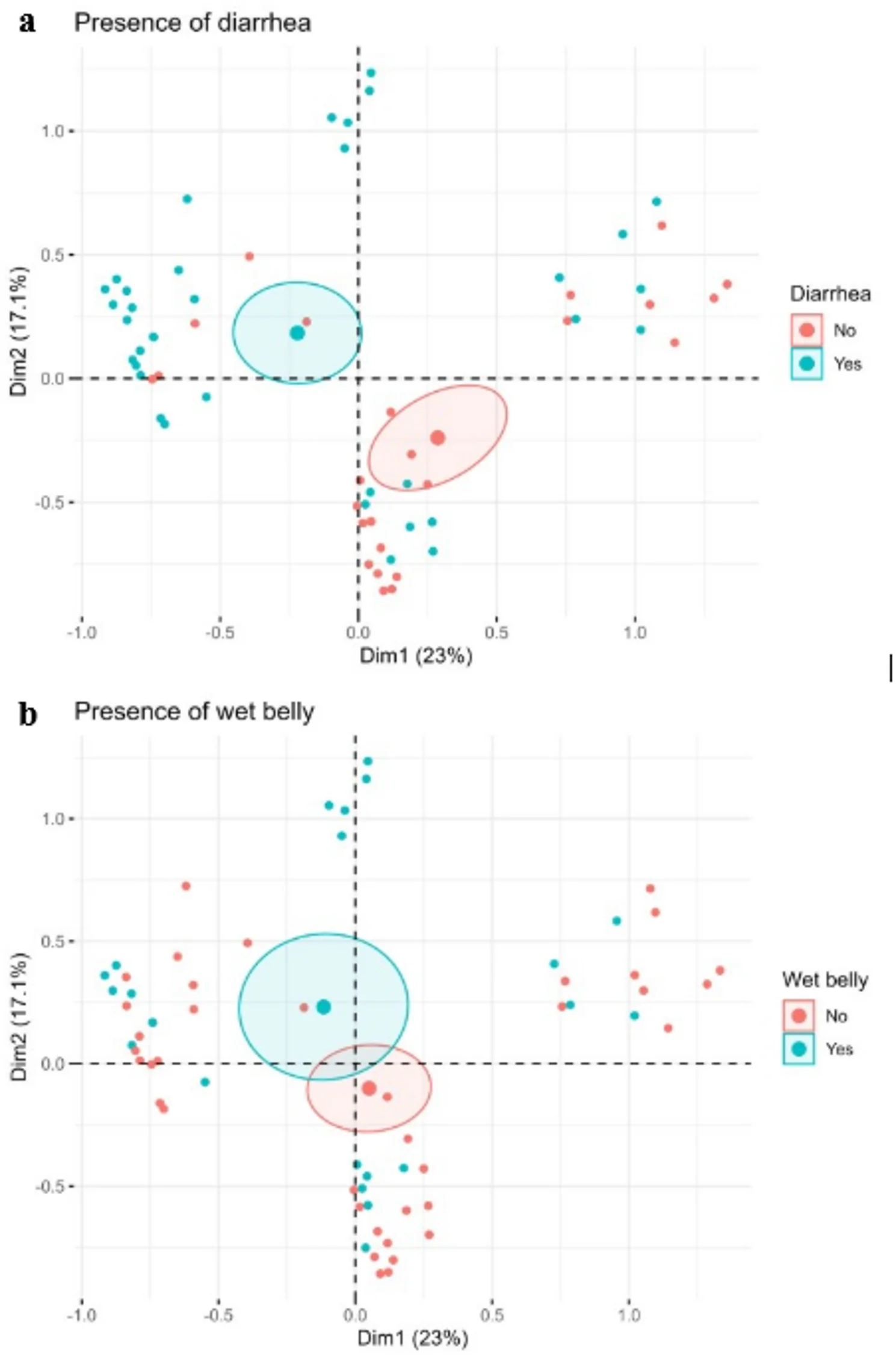
Ordination plot of observations of diarrhoea and wet belly in reindeer during the winter of 2019–2020. The presence (yes, blue) and absence (no, red) of diarrhoea (**a**) and wet belly (**b**) and their confidence ellipses are visualized in a plot defined by a multiple corresponding analysis (MCA) model, mapping the relationships between exposure variables (demography and feeding) and outcome variables (disease observation) in dimensions 1 and 2. The percentages for each dimension represent the proportion of variance in the model explained by the dimension. Points represent the distribution of all 76 herds in the survey in dimensions 1 and 2

Observation of diarrhoea was the only disease variable that showed to be distinctively associated with an exposure variable, having the highest discriminatory value in the MCA, also appearing in a cluster defined by the HCA, where reports of diarrhoea and feeding in an enclosure were located in the same cluster (Cluster 1), with fully non-overlapping confidence ellipses (Fig. 5a). In contrast, the absence of diarrhoea appeared more frequently in the lower right quadrant, together with feeding on pasture. The observation of wet belly followed a similar cluster pattern, but ellipses were overlapping (Fig. 5b), indicating that this condition does not separate as distinctly in the HCA model, with reports of wet belly more evenly distributed across clusters, rather than being clearly associated with one. Complementary MCA and HCA plots are included in additional files (see Additional file 4 and 5, respectively).

**Fig. 6:**
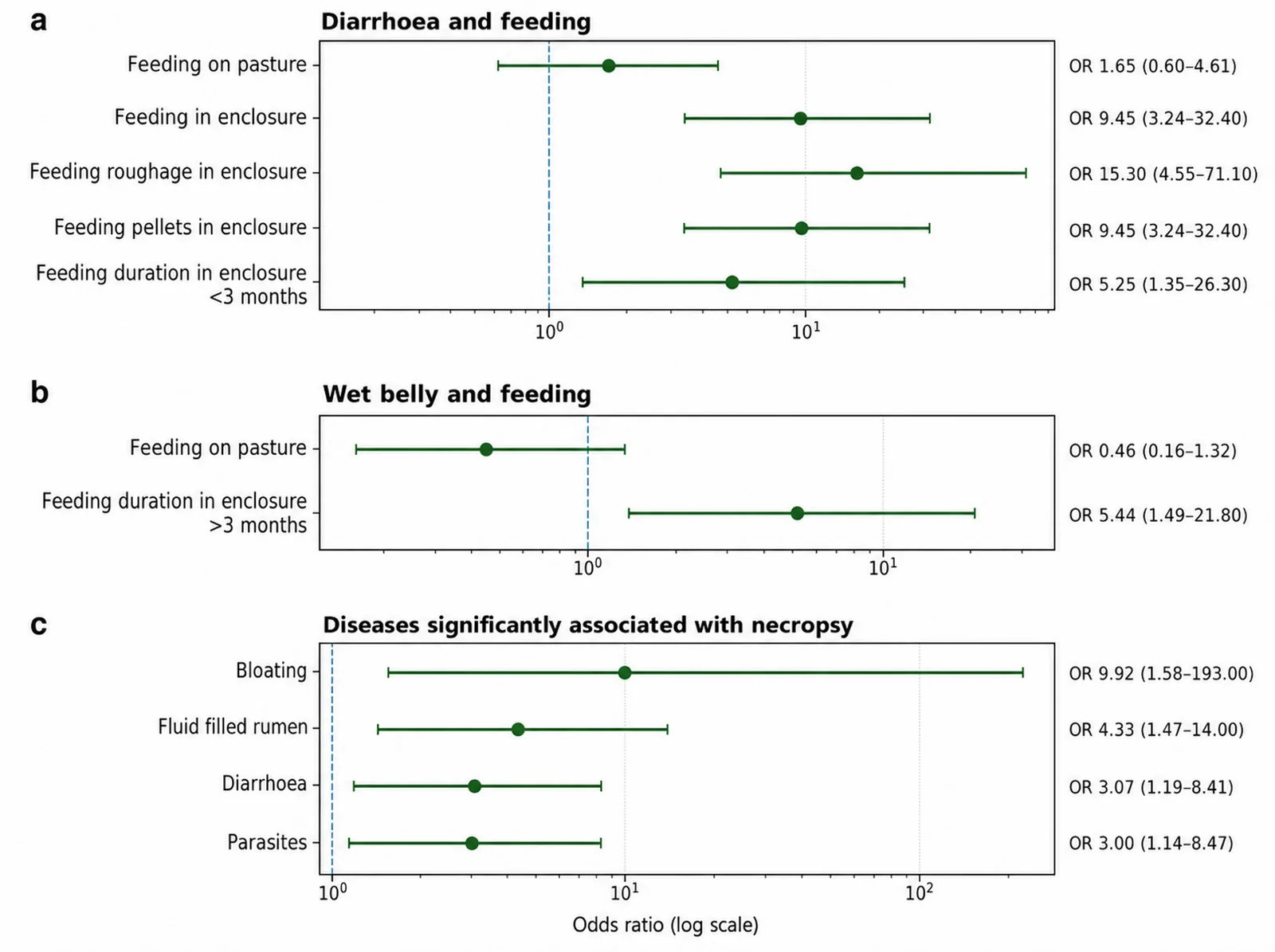
Associations between feeding strategy, disease and necropsy reported by reindeer herders in Norway and Sweden. Forest plots show odds ratios (ORs) with 95% confidence intervals from univariable logistic regression analyses. Panel **a** shows associations between feeding strategy and diarrhoea, including reference (no feeding), feeding on pasture, and enclosure-based feeding variables. Panel ** b** shows associations between feeding strategy and wet belly, including feeding on pasture and duration of enclosure feeding. Panel ** c** shows diseases significantly associated with necropsy. Points represent odds ratios and horizontal lines represent 95% confidence intervals, plotted on a logarithmic scale

### Logistic regression analysis

Logistic regression analysis showed that the observation of diarrhoea was significantly more common with feeding in enclosures (OR varied between 9 and 15 for categories including feeding in enclosure (*P* < 0.001), with the highest odds with feeding a combination of pellets and roughage (Fig. 6a). Reports of wet belly and unspecified mortality were more common with longer feeding duration (> 3 months) in enclosures (OR 5.44; *P* = 0.012 and OR 4.5; *P* = 0.02, respectively) (Fig. 6b). Wet belly and diarrhoea were both significantly more commonly observed in Sweden than in Norway (OR 7.92; *P* = 0.002 and OR 5.14; *P* = 0.005, respectively).

The conditions of fluid-filled rumen, bloating, brainworm and parasites were more often reported from herds where necropsies had been performed, either by herder or by a veterinarian (Fig 6c). For the observation of parasites, most feeding factors were not significant predictors, except for slightly lower odds with feeding roughage on pasture (OR 0.29; *P* = 0.017). No significant associations were identified between feeding and necrobacillosis, contagious ecthyma (parapoxvirus), pneumonia, or ataxia. Complete model output is provided in Additional file 6.

### Veterinary health services

Among respondents, 18 (24%) reported having done necropsies themselves, and 26 (34%) reported necropsy performed by a veterinarian. From a total of 69 responses (9 no-answers excluded) a total of 38 respondents (55%) had requested veterinary assistance for disease issues, whereas the rest (45%) had not. Rates were similar in Norway (50%) and Sweden (59%).

Reasons for *not* seeking veterinary help included the perceived lack of knowledge and expertise among veterinarians on reindeer health issues (33%); belief that herders could manage the issue themselves (20%) and cost (7%). Among all respondents, 15% noted that they had access to satisfactory veterinary assistance. When asked if the animals were medically treated against warble fly (*Hypoderma tarandi*) larvae, 58 (76%) answered yes (Norway; *n* = 23/33, 70%, Sweden; *n* = 35/43, 81%).

Fisher’s tests revealed no significant associations (*P* > 0.05) between seeking veterinary assistance and herd size, geographic location, feeding practices, treatment against warble fly larvae or necropsy performed.

## Discussion

This study provides new insight into supplementary feeding practices, disease observations, and veterinary service use among reindeer herders in Norway and Sweden. Across multiple analytical approaches, feeding in enclosures emerged as the management factor most consistently associated with reported digestive disturbances, particularly diarrhoea and wet belly. The findings in this study highlight how changing feeding practices may influence reindeer health under increasingly constrained grazing conditions. Furthermore, we evaluate the role of the veterinarian in supporting the health of semi-domesticated reindeer in a One Health context.

### Methodological considerations and limitations

This study provides one of very few datasets describing feeding practices and reindeer health in Norway and Sweden, in a time of rapid environmental and management change. However, it ought to be noted that the findings must be evaluated in the light of several limitations inherent to questionnaire-based research: Owing to the voluntary nature of the survey, the distribution of responses may not be fully representative of all herding districts or herd sizes. Heterogenous response rates restrain generalization but may still provide valuable descriptive insight. All disease observations were based on herders’ own assessments and reports. The questions in the survey focused on observable signs rather than clinical diagnoses, acknowledging that herders often rely on visual assessment and practical experience. Reports may therefore represent overlapping concepts – e.g. ataxia as the sign and brainworm as the assumed cause. Some internally observable conditions (e.g., ruminal acidosis (fluid-filled rumen), bloating and parasites) were more often reported when necropsies were performed, either by the herder or by a veterinarian, indicating improved diagnostic certainty for these conditions with a necropsy.

Because some questions required memory of events over several years (2015–2021), we also acknowledge the potential for recall bias. However, major feeding events and disease outbreaks are typically memorable, and similar recall-based approaches are widely used in studies of pastoral and Indigenous livestock systems [50]. A key consideration is that herders feeding in enclosures may observe disease signs more frequently simply because animals are closer and easier to monitor, representing a detection bias.

It should be noted that MCA and HCA merely provide exploratory pattern recognition. Furthermore, the inferential GLM results should be interpreted cautiously due to limited sample sizes and potential collinearity of variables. Despite these limitations, combining descriptive statistics, MCA/HCA, and logistic regression strengthens confidence in the observed associations.

### The extent of supplementary feeding of reindeer in Norway and Sweden

Supplementary feeding was widely practiced among respondents. The proportion of respondents feeding reindeer in enclosures among those who reported feeding showed a gradual increase over time. Although this trend was not statistically significant, the consistent upward tendency suggests a potential shift towards greater use of enclosure-based feeding practices. This pattern may reflect increasing reliance on more controlled feeding strategies, possibly in response to changing environmental conditions, such as reduced pasture availability, increased snow or ice cover, or other climatic and management pressures. While the magnitude of change is modest and variable between years, the direction of the trend is consistent with a broader transition towards more intensive or regulated feeding practices in reindeer husbandry. However, given the variability between years and the limited sample size, this interpretation should be considered indicative rather than conclusive.

Most herders used a combination of pellets and roughage, with feeding often lasting beyond one month. Previously, supplementary feeding has not been considered a long-term solution to compensate for inadequate pasture resources [18, 20], but has become more common in recent decades, because of the increased challenges of restricted flexibility and accessibility of natural pastures [20, 51].

The observed patterns in the present study align with the perception that feeding is no longer limited to short emergency periods but is becoming an integrated management strategy in some regions, particularly in northern Sweden. Although herders may view supplementary feeding as a “necessary evil” to be avoided, if possible [51], they acknowledged the importance of introducing supplementary food in time for the animals’ metabolic system to adapt before starvation sets in [16]. Supplementary feeding can help maintain body condition and prevent starvation during critical periods, but on the other hand, its application breaches traditional dynamics of reindeer herding, often increasing animal density on a feeding site, restricting movement (in enclosure), and create conditions that differ markedly from free-range grazing.

The regional patterns representing differences in feeding and feeding practices likely reflect geographic differences in pasture quality and availability as well as animal density. Furthermore, the different regions are situated in different ecological and climatic zones, all of which may affect the decision of whether to feed or not, and how. Between-year variation in feeding practices may reflect temporal fluctuations in grazing conditions.

### Observations of disease – patterns and associations

Parasite observations were the most reported. It is worth noting that observation of parasites does not necessarily indicate a disease or a pronounced health problem for the host. These observations included the presence of warble fly larvae, which is very common in semidomesticated reindeer and easily observable [52]. Observation of parasites showed very limited associations with feeding strategies, although logistic regression suggested slightly lower odds of parasite observations when roughage was fed on pasture.

Diarrhoea was the second most common condition reported. Overall, 57% of herders had observed cases of diarrhoea, with a significantly higher prevalence in Sweden than in Norway Across all analytical approaches used in this study, observation of diarrhoea showed the strongest association with feeding in enclosure. Diarrhoea in reindeer is rarely seen during free-range grazing, but may occur following abrupt changes in diet, such as acute transition from natural pastures to supplementary feed, as the rumen and intestinal microbiota may be disrupted by the dietary shift [16]. Similar mechanisms are described in other ruminants, where digestive disturbances may occur in association with conditions such as rumen acidosis [27].

Diarrhoea may also be caused by certain bacterial infections or intestinal parasites [29]. Gathering points for reindeer, such as salt licks, have shown to represent potential hotspots for parasite transmission [33]. Notably, a previous study in Finland and Norway did not conclude that feeding was a significant factor for infection with endoparasites in reindeer, although it was concluded that higher animal density was a risk factor for reindeer calves to shed *Eimeria* sp. oocysts [53]. This suggests that management factors related to aggregation of animals may be as important as feeding practices themselves. In the same dataset, examined in a previous study [43], feeding in enclosures was also associated with reported outbreaks of IKC.

Diarrhoea could potentially act as a stressor and predispose to infectious diseases like IKC [43, 44], and vice versa. Although the mechanisms remain uncertain, these findings may indicate that management-related stressors associated with feeding practices could increase disease susceptibility.

Wet belly was also more frequently reported in Sweden and was associated with prolonged feeding in enclosures (> 3 months). Although the aetiology of wet belly remains unclear, previous literature [28, 29] and herders’ experiences indicate a strong link to feeding contexts. Logistic regression suggests that longer enclosure feeding may increase risk, potentially reflecting cumulative effects from dietary change and prolonged confinement. Wet belly followed a similar pattern as diarrhoea in the HCA model but had overlapping confidence ellipses, indicating that the occurrence of wet belly may be influenced by multiple factors, reinforcing the idea that while feeding plays a role, the underlying mechanisms of the condition remain uncertain [16, 28].

Although abortion was among the most reported conditions, we did not observe a distinct clustering pattern in relation to feeding strategy. Reduced fecundity in ruminants may in general be attributed to a multitude of conditions, including stress, starvation, and a wide range of infectious agents that reindeer may be exposed to [29, 34]. One example that may be relevant in a feeding context is the bacterium *Listeria monocytogenes*, which may be found in soil and in poor quality silage [29] potentially causing disease and abortion in livestock and reindeer. The protozoan parasites *Toxoplasma gondii* and *Neospora caninum* may be transferred from mother to foetus during pregnancy and are well known causes of abortion and foetal abnormalities in sheep and cattle, and may cause similar conditions in reindeer [29, 34].

For observations of necrobacillosis, there were significant differences between Norway (0%) and Sweden (14%) (*P* = 0.03). However, due to the small sample size, there is a considerable uncertainty regarding how well this reflects the actual prevalence, and the reason for the higher level in Sweden than in Norway is not known.

Similar to observations of necrobacillosis, there were no apparent clustering patterns nor significant associations with feeding practices for the less frequently reported conditions, such as signs of ruminal acidosis (fluid-filled rumen), brainworm, ataxia, bloating, pneumonia, contagious ecthyma (parapoxvirus) or unspecified mortality. The lack of significant patterns in the models may reflect multifactorial aetiologies and suggests that occurrence is likely influenced by multiple factors beyond feeding practices, potentially including unparameterized environmental conditions, other management strategies, and overall herd health. This highlights the complexity of health issues in reindeer herds and underscores the need for further investigation into underlying causes and contributing factors.

Internal conditions such as ruminal acidosis, bloating, and brainworm were more likely to be reported when necropsies were performed, highlighting the role of necropsy in revealing otherwise less visible conditions.

### The role of the veterinarian

More than half of the respondents had contacted a veterinarian regarding disease issues, but many reported barriers, such as limited access, cost, and perceived lack of expertise in reindeer-specific issues. These findings indicate that veterinary involvement in reindeer health management still is constrained by structural and practical challenges. The finding that some diseases were more often detected in herds where necropsy was performed underscores the value of such examination and veterinary input. In some cases, necropsy was conducted without consulting a veterinarian, indicating initiative to investigate the disease and cause of death, but a lack of motivation to contact a veterinarian.

A previous questionnaire study in Norway and Sweden (2010 and preceding years) found that only 4 out of 57 respondents (7%) would consider consulting a veterinarian if one or a few of their reindeer showed symptoms of IKC [54]. That study also reported that 58% of respondents had access to a veterinarian, 31% had access only part of the time, and 10% had no access. In the present study, 55% of herders reported seeking veterinary assistance, which may indicate a gradual increase in veterinary service use in reindeer husbandry.

Access to veterinarians with expertise in reindeer health is crucial not only for animal welfare, but also for food safety, economic resilience, and the safeguarding of Sámi livelihoods and cultural integrity [55]. Given the close interconnections between livestock health, environmental conditions, and human well-being, strengthening veterinary capacity in this field aligns closely with One Health principles [56]. Veterinarians play a key role within this framework through their expertise in diagnostics, treatment, population medicine, epidemiology, zoonotic disease surveillance, and food safety, as well as being in position to communicate scientific knowledge and management practices to the public. Enhancing veterinary competence in reindeer health and welfare is becoming increasingly important in the face of climate change, emerging diseases [42, 57], and ongoing transitions in reindeer husbandry, including the growing reliance on supplementary feeding.

Despite the presence of approximately 465,000 semi-domesticated reindeer in Norway and Sweden, education on reindeer health and diseases is currently not thoroughly integrated in the veterinary curricula in either country. While reindeer share similarities with other ruminants, their predominantly free-ranging lifestyle, seasonal variations in forage availability and body condition, and vulnerability to environmental stressors require species-specific veterinary expertise. In addition to health conditions and diseases associated with feeding practices, reindeer may be affected by diseases that can spread rapidly within herds and potentially be transmitted to wildlife, livestock or humans. Examples include elaphostrongylosis (brainworm), pasteurellosis, IKC, contagious ecthyma (parapoxvirus) and necrobacillosis [29, 41, 44]. In addition, we know that semi-domesticated reindeer, like other wild and domesticated ruminants, are susceptible to foot and mouth disease, anthrax, chronic wasting disease and several emerging vector-borne diseases and may become important components in larger disease outbreaks.

### Future directions

A framework for monitoring reindeer health and disease ecology that includes first-hand information from herders is essential, building contact and trust between reindeer herders and veterinary professionals [57]. Initiatives to strengthen veterinary support for reindeer husbandry in Sweden [58] and the recently established Reindeer Health Advisory Service in Norway [59] represent important steps in this direction. However, further evaluation is needed to determine how these initiatives can best address the needs and challenges facing reindeer and their herders. Continued investment in reindeer research and health management will therefore be important to support both animal and human health in a rapidly changing environment [60].

## Conclusions

Supplementary feeding appeared widely practiced among reindeer herders in Norway and Sweden during 2015–2021, with an increase of feeding in enclosure in both countries. Feeding in enclosures was significantly associated with observations of diarrhoea, and wet belly and mortality of unknown causes were more likely seen when feeding in enclosures over a prolonged time. The transition in reindeer herding management practices and the potential influence of different feeding practices on the occurrence of disease in reindeer needs to be further elucidated.

Limited access to and confidence in veterinary services were evident. To maintain reindeer health and welfare and contribute to the support of herding communities and livelihoods, veterinarians need solid insight into reindeer husbandry systems and expertise in reindeer health, food safety, as well as disease prevention and preparedness.

Adopting an integrated One Health approach, linking traditional and local knowledge with interdisciplinary science and effective veterinary support, is crucial for resilience in reindeer husbandry and for a strengthened preparedness for emerging transmissible diseases affecting both reindeer, other livestock and wildlife populations.

## Supplementary Information

Below is the link to the electronic supplementary material.

Additional file 1. The questionnaire survey (in Norwegian) regarding health and supplementary feeding of semi-domesticated reindeer, distributed among reindeer herders in Norway and Sweden in 2021 (*.pdf).

Additional file 2. The questionnaire survey (in Swedish) regarding health and supplementary feeding of semi-domesticated reindeer, distributed among reindeer herders in Norway and Sweden in 2021 (*.pdf).

Additional file 3. The questionnaire survey (in Northern Sámi) regarding health and supplementary feeding of semi-domesticated reindeer, distributed among reindeer herders in Norway and Sweden in 2021 (*.pdf).

Additional file 4. Plots of exposure (active) variables in the survey. Plot of answers relating to active variables, mapped on the herd distribution defined in the MCA model in dimensions 1 and 2, and their confidence ellipses (*.png).

Additional file 5. Plots of outcome (passive) disease variables. Plot of answers relating to disease variables (other than diarrhoea and wet belly), mapped on the herd distribution defined in the MCA model in dimensions 1 and 2, and their confidence ellipses (*.png).

Additional file 6. Tables with overview over univariate and multivariate logistic regression model output.

## Data Availability

The datasets presented in this article are not readily available because the data encompass personal accounts from identifiable reindeer herders. Requests to access additional data can be directed to the corresponding author.
