# Supplementary material for: Reindeer health and supplementary feeding practices – a survey among herders in Norway and Sweden
Source: Acta Vet Scand. 2026 Sep 22;68:36. doi: 10.1186/s13028-026-00868-3 (PMC13595754; doi:10.1186/s13028-026-00868-3)
Supplement: Supplementary file 1 [file 13028_2026_868_MOESM1_ESM.pdf]

Additional file 1 The questionnaire survey (in Norwegian) regarding health and supplementary feeding of semi-domesticated reindeer, distributed among reindeer herders in Norway and Sweden in 2021.

Velkommen!

Denne spørreundersøkelsen er rettet mot deg som driver reindrift i Norge eller Sverige, uavhengig av om du fôrer eller ikke. Undersøkelsen forventes å ta opptil 30 minutter å besvare og er delt inn i flere avsnitt:

1. Spørsmål om tre smittsomme sykdommer og om øvrig reinsdyrhelse.

2. Spørsmål om fôring (effekter, rutiner og annet).

OBS: Svarene dine LAGRES IKKE hvis du stopper midt i undersøkelsen, og det er heller IKKE MULIGT Å GÅ TILBAKE i undersøkelsen, da forsvinner alle svarene og du blir nødt til å starte på nytt.

Spørgmål merket med * er obligatoriske å svare for å fortsette.

Med mindre annet er oppgitt, er bildene i undersøkelsen hentet fra SSR:s bildearkiv.

Identiteten din forblir skjult.

Med mindre annet er oppgitt, henviser spørsmålene i undersøkelsen til vinterværforhold i løpet av 2019-2020.

Spørgsmålene gjelder personen som svarer på undersøkelsen.

1) * I hvilket land driver du hovedsakelig med reindrift?

- ○ Norge
- ○ Sverige

Denne informasjonen vises kun i forhåndsvisningen

Følgende betingelser må være oppfylt for at spørsmålet skal vises for respondenten:

Dersom spørsmålet I vilket land bedriver du huvudsakligen renskötsel? inneholder noen av disse alternativene

- Sverige

2) * I hvilken region driver du hovedsakelig med reindrift?

- ○ Dalarna/Jämtland
- ○ Västerbotten

- Norrbotten

Denne informasjonen vises kun i forhåndsvisningen

Følgende betingelser må være oppfylt for at spørsmålet skal vises for respondenten:

Dersom spørsmålet I vilket land bedriver du huvudsakligen renskötsel? inneholder noen av disse alternativene

- Sverige

3) * Angi i hvilken type sameby du driver med reindrift?

- Fjällsameby
- Skogssameby
- Koncessionssameby

Denne informasjonen vises kun i forhåndsvisningen

Følgende betingelser må være oppfylt for at spørsmålet skal vises for respondenten:

Dersom spørsmålet I vilket land bedriver du huvudsakligen renskötsel? inneholder noen av disse alternativene

- Norge

4) * I hvilken region driver du hovedsakelig med reindrift?

- Øst Finnmark
- Vest Finnmark
- Troms
- Nordland
- Nord-Trøndelag
- Sør-Trøndelag
- Møre- og Romsdal
- Hedmark

Denne informasjonen vises kun i forhåndsvisningen

Følgende betingelser må være oppfylt for at spørsmålet skal vises for respondenten:

Dersom spørsmålet I vilket land bedriver du huvudsakligen renskötsel? inneholder noen av disse alternativene

- Norge

5) * Hvor lang erfaring har du med å arbeide med reindrift?

- Under 5 år
- 5-9 år

- 10-29 år
- Over 30 år

6) Oppgi kjønn?

- Kvinne
- Mann
- Vil ikke svare

7) Oppgi din alder?

- Yngre enn 20
- 20-39
- 40-59
- Over 60

8) * Hva er det omtrentlige antallet dyr i din reinflokk (vinterflokk) etter årlig slakting?

- Under 50
- 50-99
- 100-249
- 250-499
- 500-999
- 1000-1999
- 2000-2999
- Over 3000

Her følger spørsmål om tre infeksjonssykdommer som kan være relatert til fôring.

1. Smittsom øyebetennels/øyeforandring:

Smittsom øyebetennels/øyeforandring kan starte med tåreflod som gir fuktighet i pelsen under øynene. Sykdommen kan være forårsaket av både virus og bakterier og er derfor smittsom og kan gi opphav til ulike øyeforandringer, som f.eks. vist på Bilde 1 A-C.

Bilde 1. A-C:

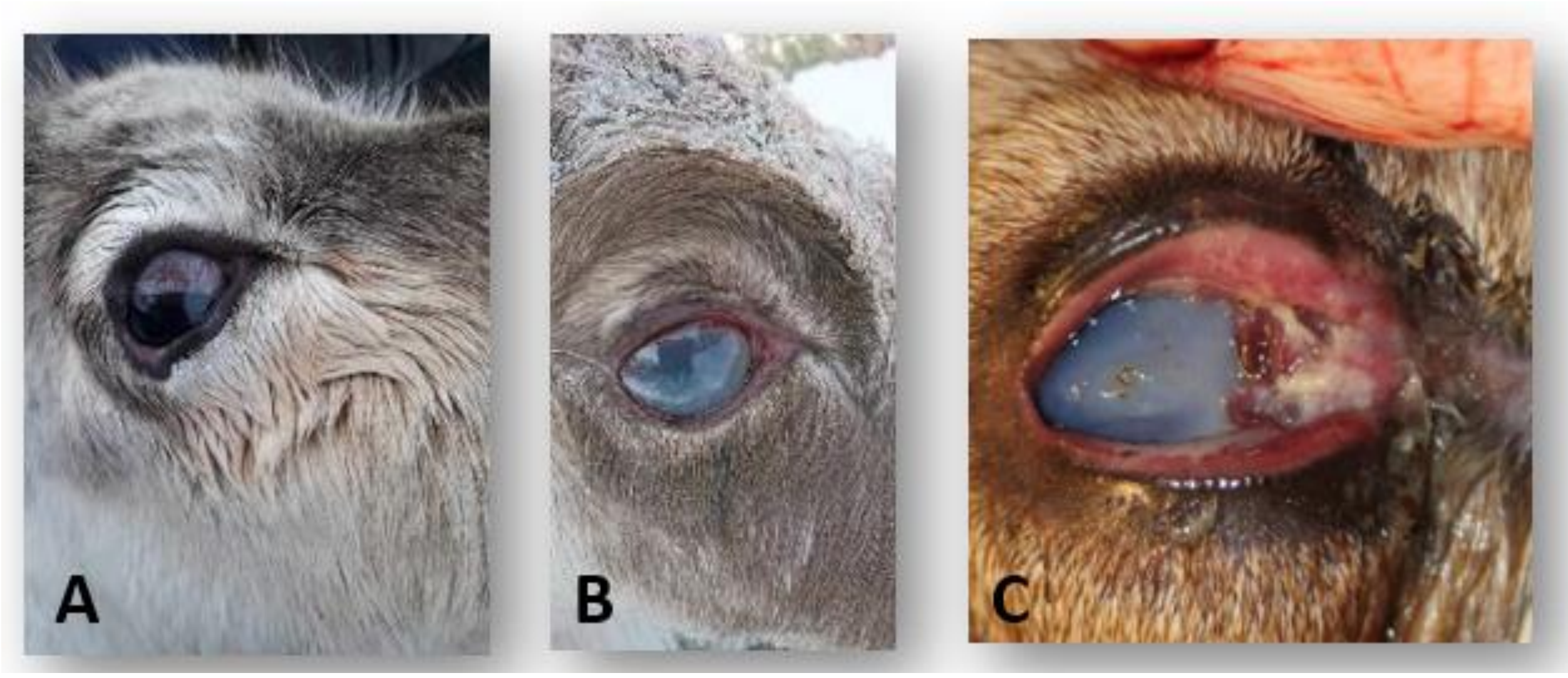

A: Tåreflod som gir fuktighet i pelsen under øynene.

B: Blågrå hornhinne og rød slimhinne, økende symptomer.

C: Rød slimhinne, hovent øye og vedvarende tåreflod, økende symptomer.

9) * Har du sett lignende øyeforandringer som bilde 1. A-C viser, på dine reinsdyr de siste 10 årene?

- Ja
- Nei
- Vet ikke / annen øyeforandring, beskriv under

10) Beskriv hvilken annen øyeforandring du har sett:

Denne informasjonen vises kun i forhåndsvisningen

Følgende betingelser må være oppfylt for at spørsmålet skal vises for respondenten:

Dersom spørsmålet Har du sett liknande ögonförändringar som Bild 1. A-C visar på dina renar under de senaste 10 åren? inneholder noen av disse alternativene

- Vet ikke / annen øyeforandring, beskriv under
- Ja

Smittsom øyebetennelse/øyeforandring

Bild 1. A-C:

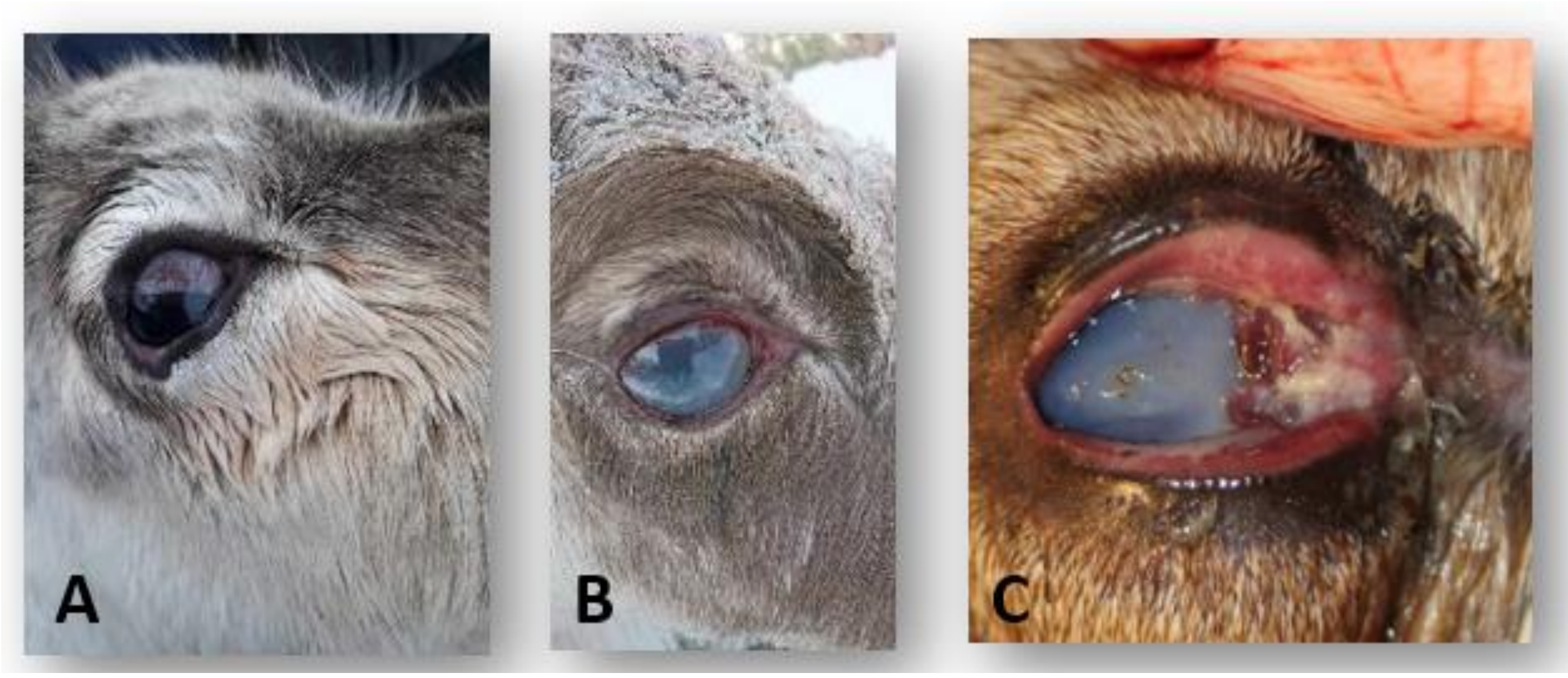

11) När så du sist lignende forandringer som vist på bilde 1. A-C? Om du har oppgitt en annen øyeforandring / symptom, svarer du på følgende spørsmål om øyebetennelse / øyeforandring basert på det.

- ○ Det siste året
- ○ Ikke det siste året men de siste 5 årene
- ○ Mer enn 5 år siden
- ○ Vet ikke

Denne informasjonen vises kun i forhåndsvisningen

Følgende betingelser må være oppfylt for at spørsmålet skal vises for respondenten:

Dersom spørsmålet Har du sett liknande ögonförändringar som Bild 1. A-C visar på dina renar under de senaste 10 åren? inneholder noen av disse alternativene

- Vet ikke / annen øyeforandring, beskriv under
- Ja

Smittsom øyebetennelse/øyeforandring

Bild 1. A-C:

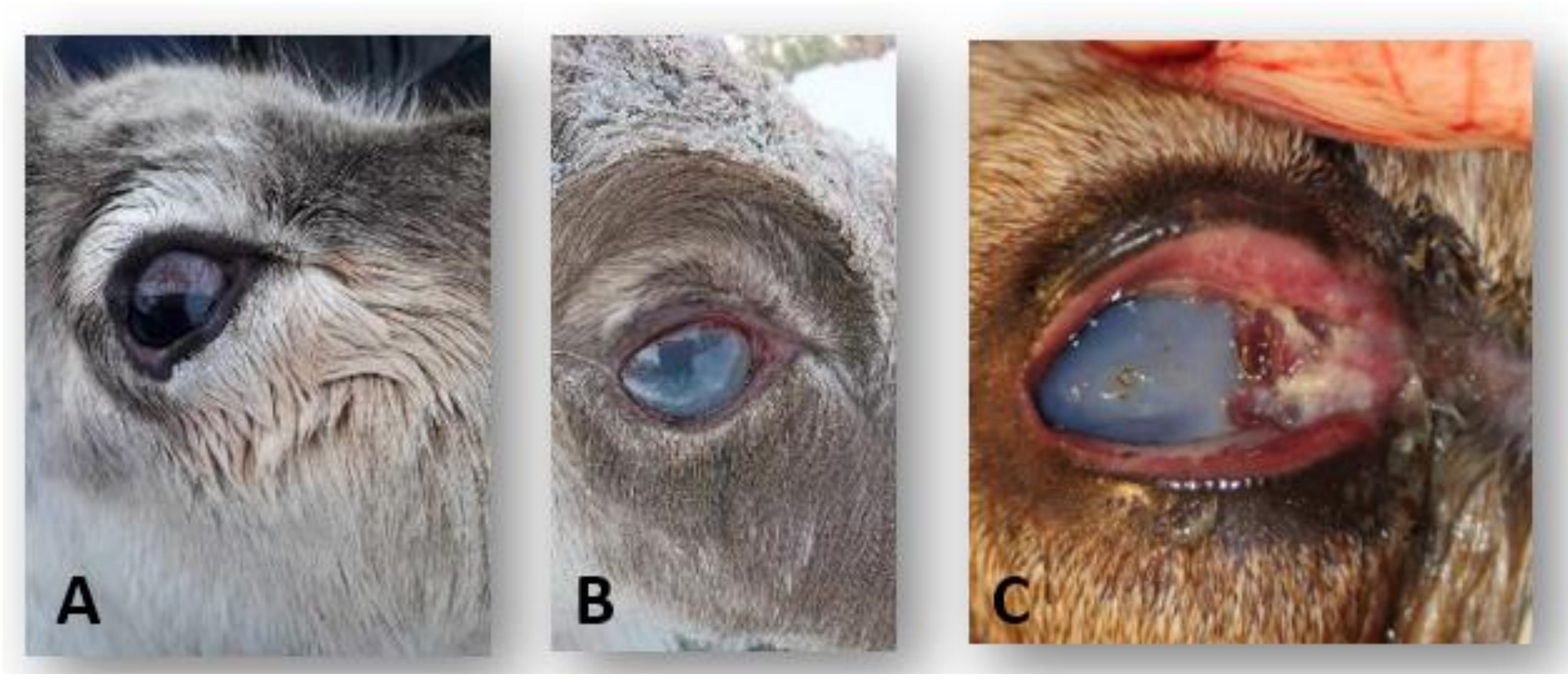

12) Hvilken av bildene A-C ser du som oftest på berørte reinsdyr?

	Aldri	Noen ganger	Ofte
A	○	○	○
B	○	○	○
C	○	○	○
Annet symptom, oppgis i kommentarfeltet nedenfor	○	○	○

Denne informasjonen vises kun i forhåndsvisningen

Følgende betingelser må være oppfylt for at spørsmålet skal vises for respondenten:

Dersom spørsmålet Har du sett liknande ögonförändringar som Bild 1. A-C visar på dina renar under de senaste 10 åren? inneholder noen av disse alternativene

- Vet ikke / annen øyeforandring, beskriv under
- Ja

13) Plass for kommentarer:

Denne informasjonen vises kun i forhåndsvisningen

Følgende betingelser må være oppfylt for at spørsmålet skal vises for respondenten:

Dersom spørsmålet Har du sett liknande ögonförändringar som Bild 1. A-C visar på dina renar under de senaste 10 åren? inneholder noen av disse alternativene

- Vet ikke / annen øyeforandring, beskriv under
- Ja

Smittsom øyebetennelse/øyeforandring

14) Når på året ser du flest antall reinsdyr med smittsom øyebetennelse/øyeforandring? Oppgi for kalv, ungdyr og voksne dyr. Det er mulig å velge flere sesonger.

	Ikke observert	Vår	Sommer	Høst	Vinter	Året rundt (oppgi som det eneste alternativet!)
Kalv (yngre enn 1 år)	☐	☐	☐	☐	☐	☐
Unge dyr (1-3 år)	☐	☐	☐	☐	☐	☐
Voksne djur (eldre enn 3 år)	☐	☐	☐	☐	☐	☐

Denne informasjonen vises kun i forhåndsvisningen

Følgende betingelser må være oppfylt for at spørsmålet skal vises for respondenten:

Dersom spørsmålet Har du sett liknande ögonförändringar som Bild 1. A-C visar på dina renar under de senaste 10 åren? inneholder noen av disse alternativene

- Vet ikke / annen øyeforandring, beskriv under
- Ja

15) Plass for kommentarer:

Denne informasjonen vises kun i forhåndsvisningen

Følgende betingelser må være oppfylt for at spørsmålet skal vises for respondenten:

Dersom spørsmålet Har du sett liknande ögonförändringar som Bild 1. A-C visar på dina renar under de senaste 10 åren? inneholder noen av disse alternativene

- Vet ikke / annen øyeforandring, beskriv under
- Ja

Smittsom øyebetennelse / øyeforandring

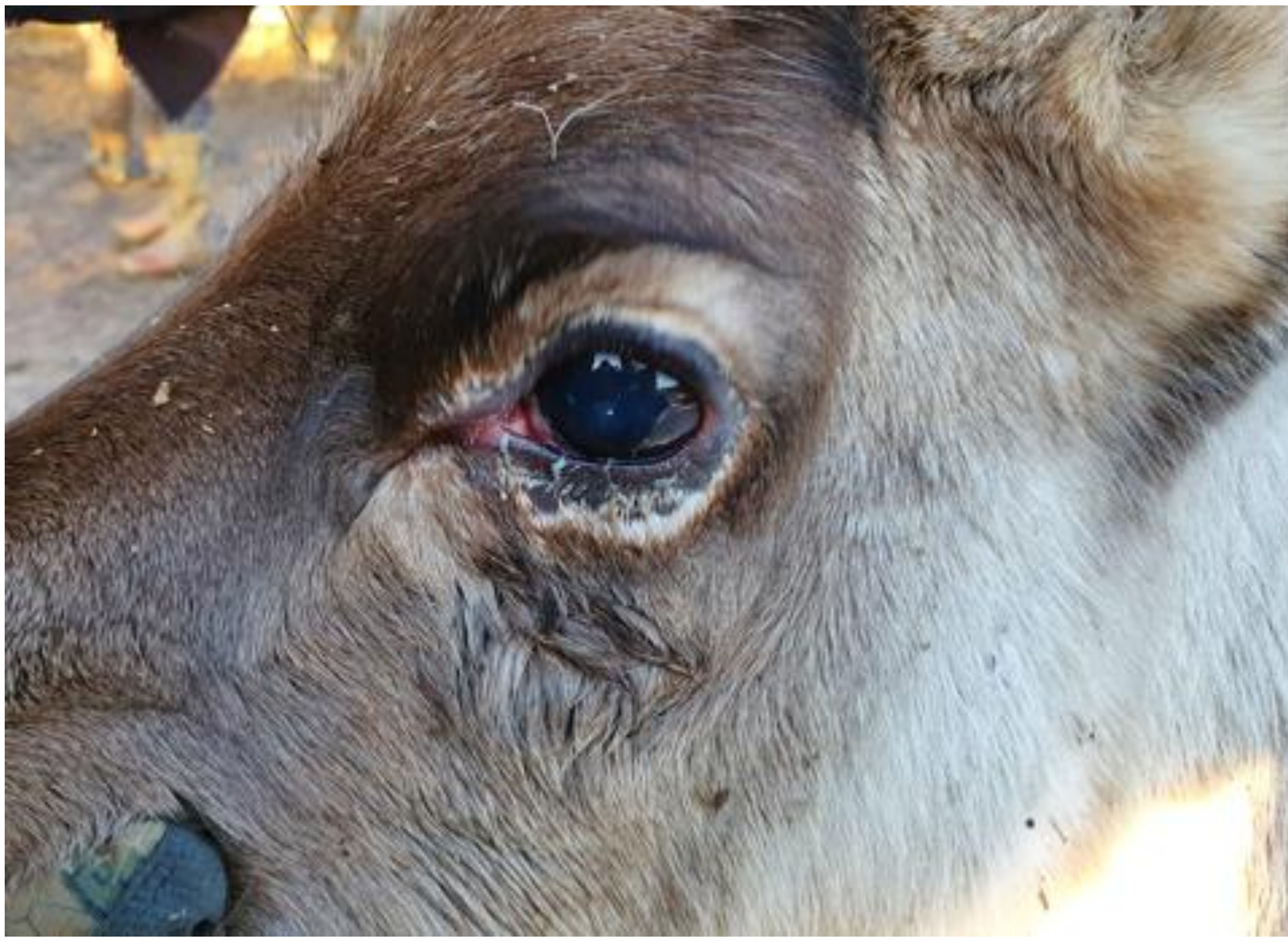

Rein med tåreflod.

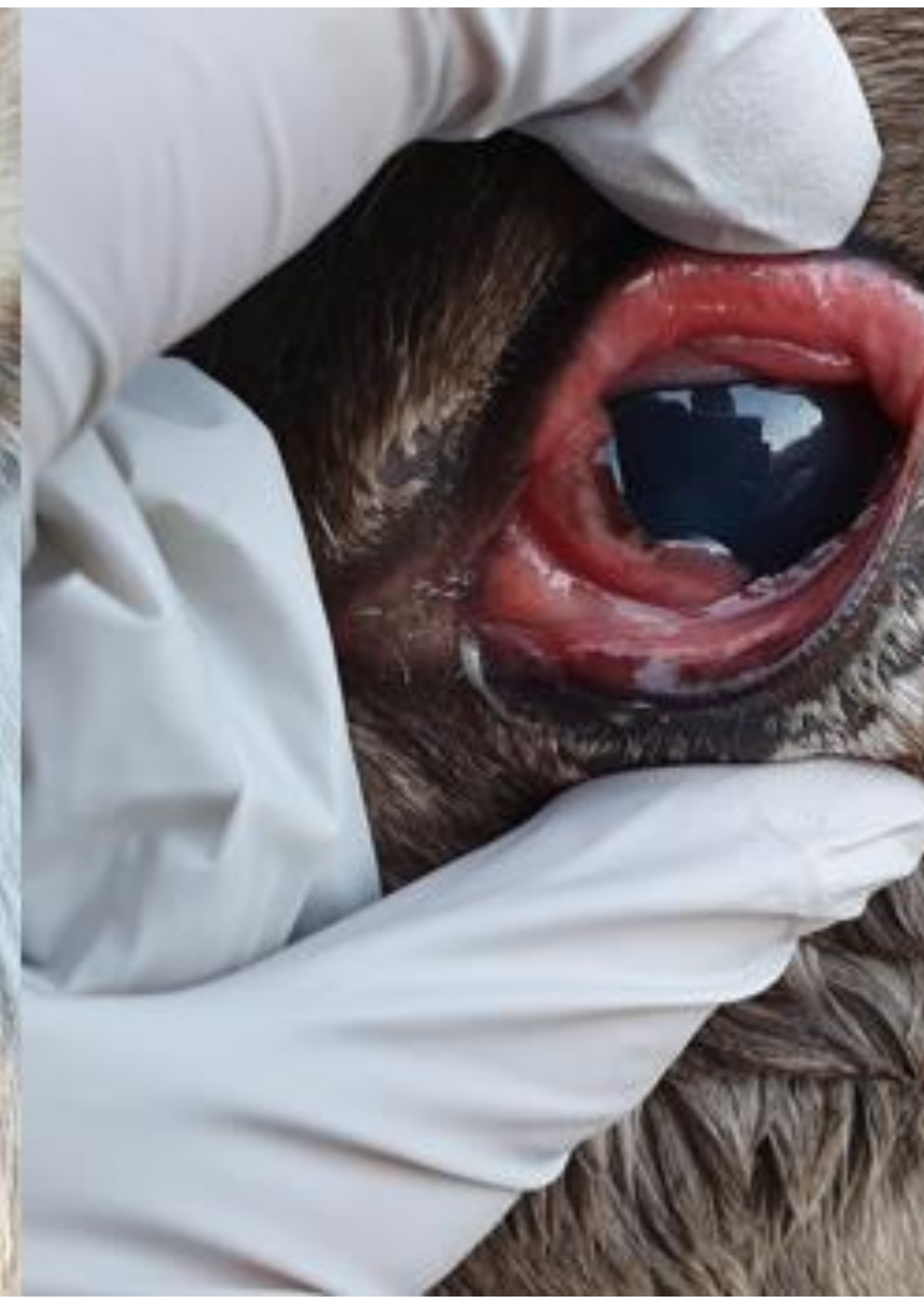

Ved nærmere undersøkelse har reinen et hovent og rødt øye.

16) Oppgi, for hver av sesongene nedenfor, hvor reinen befant seg under utbrudd av smittsom øyebetennelse / øyeforandring. Alternativt at ingen utbrudd fant sted. Utbrudd defineres som en betydelig økning i antall tilfeller over en viss tidsperiode, for eksempel flere berørte dyr enn vanlig i løpet av en sesong, eller over en kortere tidsperiode, for eksempel noen få uker.

	Ikke opplevd utbrudd	Utbrudd i innhegningen	Utbrudd på fribeite med fôring	Utbrudd på fribeite uten fôring
2015/2016	☐	☐	☐	☐
2016/2017	☐	☐	☐	☐
2017/2018	☐	☐	☐	☐
2018/2019	☐	☐	☐	☐
2019/2020	☐	☐	☐	☐
2020/2021	☐	☐	☐	☐

Denne informasjonen vises kun i forhåndsvisningen

Følgende betingelser må være oppfylt for at spørsmålet skal vises for respondenten:

Dersom spørsmålet Har du sett liknande ögonförändringar som Bild 1. A-C visar på dina renar under de senaste 10 åren? inneholder noen av disse alternativene

- Vet ikke / annen øyeforandring, beskriv under
- Ja

17) Plass for kommentarer:

Denne informasjonen vises kun i forhåndsvisningen

Følgende betingelser må være oppfylt for at spørsmålet skal vises for respondenten:

Dersom spørsmålet Har du sett liknande ögonförändringar som Bild 1. A-C visar på dina renar under de senaste 10 åren? inneholder noen av disse alternativene

- Vet ikke / annen øyeforandring, beskriv under
- Ja

Smittsom øyebetennelse / øyeforandring

18) Omtrent hvor mange reinsdyr ble berørt i gruppen der det sist var utbrudd, alternativt tilfeller av smittsom øyebetennelse / øyeforandring? Oppgi i første omgang antallet ved utbrudd om du har opplevd det.

Antall kalv (yngre enn 1 år)

Antall unge dyr (1-3 år)

Antall voksne (eldre enn 3 år)

Totalt antall reinsdyr i den berørte gruppen på tidspunktet for utbruddet (både friske og syke)

Denne informasjonen vises kun i forhåndsvisningen

Følgende betingelser må være oppfylt for at spørsmålet skal vises for respondenten:

Dersom spørsmålet Har du sett liknande ögonförändringar som Bild 1. A-C visar på dina renar under de senaste 10 åren? inneholder noen av disse alternativene

- Vet ikke / annen øyeforandring, beskriv under
- Ja

19) Plass for kommentarer:

Denne informasjonen vises kun i forhåndsvisningen

Følgende betingelser må være oppfylt for at spørsmålet skal vises for respondenten:

Dersom spørsmålet Har du sett liknande ögonförändringar som Bild 1. A-C visar på dina renar under de senaste 10 åren? inneholder noen av disse alternativene

- Vet ikke / annen øyeforandring, beskriv under
- Ja

Smittsom øyebetennelse / øyeforandring

20) * Opplever du at antallet reinsdyr som berøres av smittsom øyebetennelse / øyeforandring har endret seg de siste 5 årene?

- ○ Ja, forekomsten har økt
- ○ Ja, avtagende forekomst
- ○ Nei, sykdomsfrekvensen er uendret
- ○ Vet ikke

Denne informasjonen vises kun i forhåndsvisningen

Følgende betingelser må være oppfylt for at spørsmålet skal vises for respondenten:

Dersom spørsmålet Har du sett liknande ögonförändringar som Bild 1. A-C visar på dina renar under de senaste 10 åren? inneholder noen av disse alternativene

- Vet ikke / annen øyeforandring, beskriv under
- Ja

og

Dersom spørsmålet Upplever du att antalet renar som drabbats av smittsam ögoninflammation/ögonförändring har förändrats över de senaste fem åren? inneholder noen av disse alternativene

- Ja, forekomsten har økt

Smittsom øyebetennelse / øyeforandring

21) Hva tror du denne økningen kan skyldes?

Denne informasjonen vises kun i forhåndsvisningen

Følgende betingelser må være oppfylt for at spørsmålet skal vises for respondenten:

Dersom spørsmålet Har du sett liknande ögonförändringar som Bild 1. A-C visar på dina renar under de senaste 10 åren? inneholder noen av disse alternativene

- Vet ikke / annen øyeforandring, beskriv under
- Ja

og

Dersom spørsmålet Upplever du att antalet renar som drabbats av smittsam ögoninflammation/ögonförändring har förändrats över de senaste fem åren? inneholder noen av disse alternativene

- Ja, avtagende forekomst

Smittsom øyebetennelse / øyeforandring

22) Hva tror du denne avtagende forekomst kan skyldes?

Denne informasjonen vises kun i forhåndsvisningen

Følgende betingelser må være oppfylt for at spørsmålet skal vises for respondenten:

Dersom spørsmålet Har du sett liknande ögonförändringar som Bild 1. A-C visar på dina renar under de senaste 10 åren? inneholder noen av disse alternativene

- Vet ikke / annen øyeforandring, beskriv under
- Ja

Smittsom øyebetennelse / øyeforandring

23) * Gjøres det vanligvis tiltak (f.eks. gruppering, slakting eller annen håndtering) når du ser reinsdyr berørt av smittsom øyebetennelse / øyeforandring?

- ○ Ja
- ○ Nei
- ○ Vet ikke

Denne informasjonen vises kun i forhåndsvisningen

Følgende betingelser må være oppfylt for at spørsmålet skal vises for respondenten:

Dersom spørsmålet Har du sett liknande ögonförändringar som Bild 1. A-C visar på dina renar under de senaste 10 åren? inneholder noen av disse alternativene

- Vet ikke / annen øyeforandring, beskriv under
- Ja

24) Hvilke tiltak ble iverksatt da du sist så reinsdyr berørt av smittsom øyebetennelse / øyeforandring? Oppgi alle tiltak som ble satt i verk.

- ☐ Ingen tiltak
- ☐ Gruppering av berørte dyr i eget gjerde for syke dyr
- ☐ Veterinær ble kontaktet
- ☐ Antibiotika som gis med sprøyte i muskel
- ☐ Antibiotika i øyet
- ☐ Slakt
- ☐ Avliving/kassering
- ☐ Behandling med andre legemidler, oppgi i kommentarfeltet under
- ☐ Annet, forklar i kommentarfeltet under

Denne informasjonen vises kun i forhåndsvisningen

Følgende betingelser må være oppfylt for at spørsmålet skal vises for respondenten:

Dersom spørsmålet Har du sett liknande ögonförändringar som Bild 1. A-C visar på dina renar under de senaste 10 åren? inneholder noen av disse alternativene

- Vet ikke / annen øyeforandring, beskriv under
- Ja

25) Plass for kommentarer:

Denne informasjonen vises kun i forhåndsvisningen

Følgende betingelser må være oppfylt for at spørsmålet skal vises for respondenten:

Dersom spørsmålet Har du sett liknande ögonförändringar som Bild 1. A-C visar på dina renar under de senaste 10 åren? inneholder noen av disse alternativene

- Vet ikke / annen øyeforandring, beskriv under
- Ja

Smittsom øyebetennelse / øyeforandring

26) Har utbrudd eller tilfeller av smittsom øyebetennelse / øyeforandring ført til økonomiske konsekvenser?

- Ja
- Nei
- Vet ikke

Denne informasjonen vises kun i forhåndsvisningen

Følgende betingelser må være oppfylt for at spørsmålet skal vises for respondenten:

Dersom spørsmålet Har du sett liknande ögonförändringar som Bild 1. A-C visar på dina renar under de senaste 10 åren? inneholder noen av disse alternativene

- Vet ikke / annen øyeforandring, beskriv under
- Ja

27) Plass for kommentarer:

Denne informasjonen vises kun i forhåndsvisningen

Følgende betingelser må være oppfylt for at spørsmålet skal vises for respondenten:

Dersom spørsmålet Har du sett liknande ögonförändringar som Bild 1. A-C visar på dina renar under de senaste 10 åren? inneholder noen av disse alternativene

- Vet ikke / annen øyeforandring, beskriv under
- Ja

Smittsom øyebetennelse / øyeforandring

Bild 1. A-C:

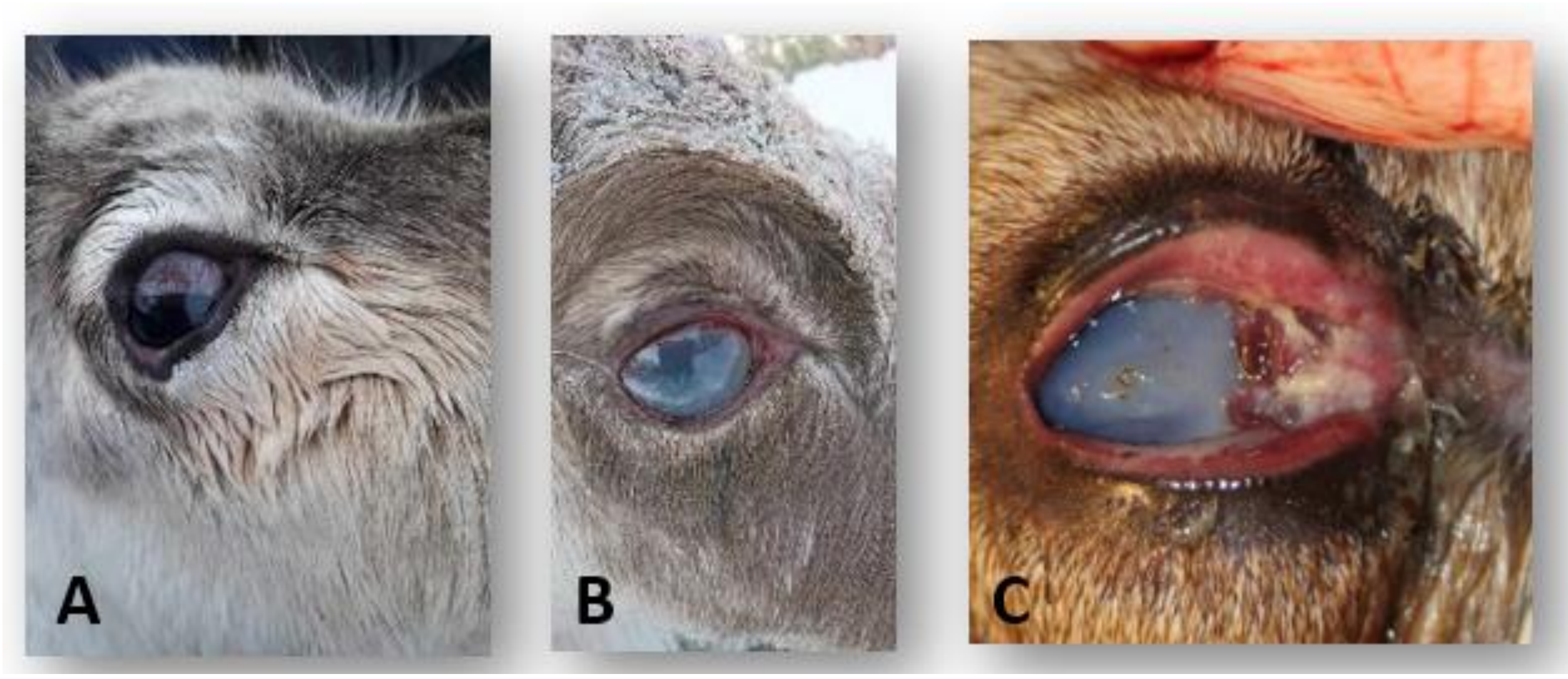

28) Har noen av forandringene på bildene et tradisjonelt navn for deg eller for Siida enheten?

- ○ Ja, oppgi i kommentarfeltet under
- ○ Nei
- ○ Vet ikke

Denne informasjonen vises kun i forhåndsvisningen

Følgende betingelser må være oppfylt for at spørsmålet skal vises for respondenten:

Dersom spørsmålet Har du sett liknande ögonförändringar som Bild 1. A-C visar på dina renar under de senaste 10 åren? inneholder noen av disse alternativene

- Vet ikke / annen øyeforandring, beskriv under
- Ja

29) Plass for kommentarer/oppgi tradisjonelt navn:

A:

B:

C:

Annen øyeforandring:

Denne informasjonen vises kun i forhåndsvisningen

Følgende betingelser må være oppfylt for at spørsmålet skal vises for respondenten:

Dersom spørsmålet Har du sett liknande ögonförändringar som Bild 1. A-C visar på dina renar under de senaste 10 åren? inneholder noen av disse alternativene

- Vet ikke / annen øyeforandring, beskriv under

- Ja

30) Kjenner du til noen tradisjonelle behandlinger av smittsom øyebetennelse / øyeforandring?

- Ja, beskriv i kommentarfeltet under
- Nei
- Vet ikke

Denne informasjonen vises kun i forhåndsvisningen

Følgende betingelser må være oppfylt for at spørsmålet skal vises for respondenten:

Dersom spørsmålet Har du sett liknande ögonförändringar som Bild 1. A-C visar på dina renar under de senaste 10 åren? inneholder noen av disse alternativene

- Vet ikke / annen øyeforandring, beskriv under
- Ja

31) Plass for kommentarer:

Denne informasjonen vises kun i forhåndsvisningen

Følgende betingelser må være oppfylt for at spørsmålet skal vises for respondenten:

Dersom spørsmålet Har du sett liknande ögonförändringar som Bild 1. A-C visar på dina renar under de senaste 10 åren? inneholder noen av disse alternativene

- Vet ikke / annen øyeforandring, beskriv under
- Nei
- Ja

Straks følger spørsmål om smittsom munnskurv (orf)

32) Legg gjerne igjen andre kommentarer relatert til smittsom øyebetennelse / øyeforandring her:

2. Her følger spørsmål om smittsom munnskurv (orf):

Bilde 2. A-B:

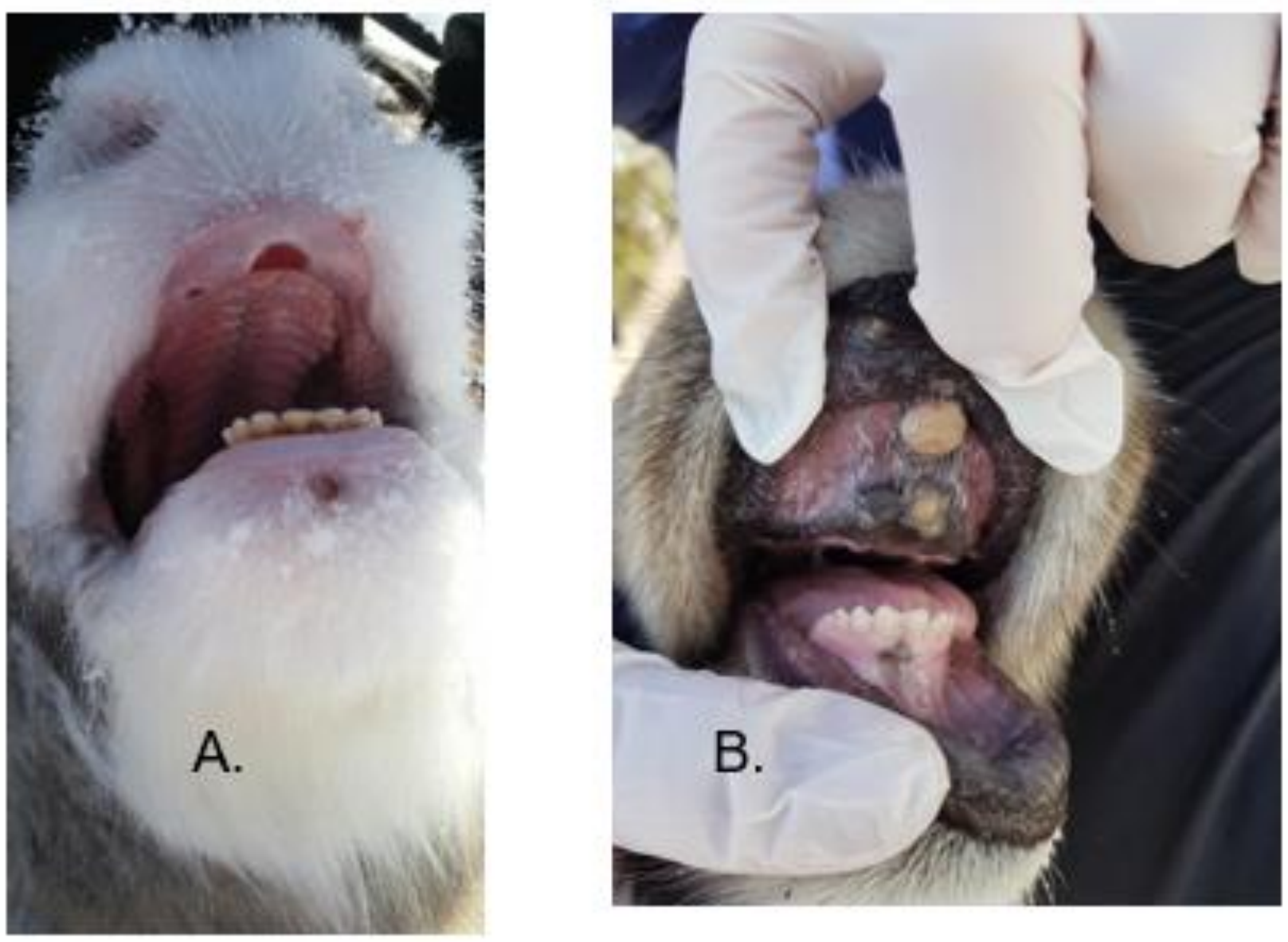

A: Sprukket blemme i munnhulen og leppen forårsaket av orfvirus, tidlig stadium.

B: Blemmer i munnhulen og tannkjøttet, senere stadium.

33) * Har du sett lignende forandringer som bilde 2. A-B viser, på dine reinsdyr de siste 10 årene?

- ○ Ja
- ○ Nei
- ○ Vet ikke

Denne informasjonen vises kun i forhåndsvisningen

Følgende betingelser må være oppfylt for at spørsmålet skal vises for respondenten:

Dersom spørsmålet Har du sett liknande förändringar som Bild 2. A-B visar på dina renar under de senaste 10 åren? inneholder noen av disse alternativene

- Vet ikke
- Ja

Smittsom munskurv (orf)

Bilde 2. A-B:

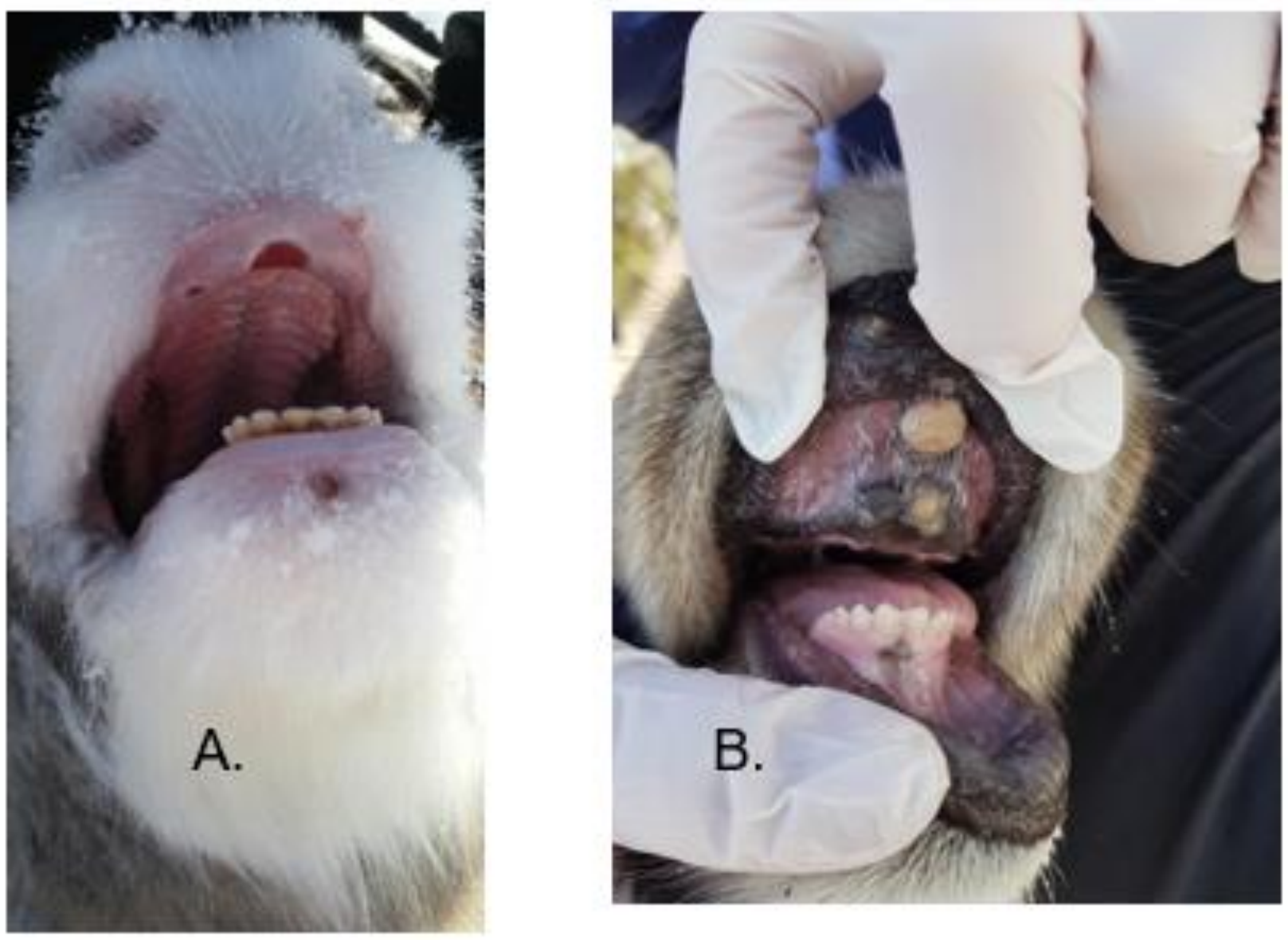

34) Når så du sist lignende forandringer som vist på bilde 2. A-B?

- Siste året
- Ikke det siste året, men for mindre enn 5 år siden
- Mer enn 5 år siden
- Vet ikke

Denne informasjonen vises kun i forhåndsvisningen

Følgende betingelser må være oppfylt for at spørsmålet skal vises for respondenten:

Dersom spørsmålet Har du sett liknande förändringar som Bild 2. A-B visar på dina renar under de senaste 10 åren? inneholder noen av disse alternativene

- Vet ikke
- Ja

Smittsom munnskurv (orf)

35) Når på året ser du flest antall reinsdyr med smittsom munnskurv (orf)? Oppgi for kalv, unge dyr og voksne dyr. Det er mulig å velge flere sesonger.

	Ikke observert	Vår	Sommer	Høst	Vinter	Året rundt (oppgi som eneste svarsalternativ!)
Kalv (yngre enn 1 år)	☐	☐	☐	☐	☐	☐
Unge dyr (1-3 år)	☐	☐	☐	☐	☐	☐
Voksen (over 3 år)	☐	☐	☐	☐	☐	☐

Denne informasjonen vises kun i forhåndsvisningen

Følgende betingelser må være oppfylt for at spørsmålet skal vises for respondenten:

Dersom spørsmålet Har du sett liknande förändringar som Bild 2. A-B visar på dina renar under de senaste 10 åren? inneholder noen av disse alternativene

- Vet ikke
- Ja

36) Plass for kommentarer:

Denne informasjonen vises kun i forhåndsvisningen

Følgende betingelser må være oppfylt for at spørsmålet skal vises for respondenten:

Dersom spørsmålet Har du sett liknande förändringar som Bild 2. A-B visar på dina renar under de senaste 10 åren? inneholder noen av disse alternativene

- Vet ikke
- Ja

Smittsom munnskurv (orf)

37) Oppgi, for hver av sesongene nedenfor, hvor reinen befant seg under utbrudd av smittsom munnskurv (orf). Alternativt at ingen utbrudd fant sted. Utbrudd defineres som

en betydelig økning i antall tilfeller over en viss tidsperiode, for eksempel flere berørte dyr enn vanlig i løpet av en sesong, eller over en kortere tidsperiode, for eksempel noen få uker.

	Ikke opplevd utbrudd	Utbrudd i innhegningen	Utbrudd på fribeite med fôring	Utbrudd på fribeite uten fôring
2015/2016	☐	☐	☐	☐
2016/2017	☐	☐	☐	☐
2017/2018	☐	☐	☐	☐
2018/2019	☐	☐	☐	☐
2019/2020	☐	☐	☐	☐
2020/2021	☐	☐	☐	☐

Denne informasjonen vises kun i forhåndsvisningen

Følgende betingelser må være oppfylt for at spørsmålet skal vises for respondenten:

Dersom spørsmålet Har du sett liknande förändringar som Bild 2. A-B visar på dina renar under de senaste 10 åren? inneholder noen av disse alternativene

- Vet ikke
- Ja

38) Plass for kommentarer:

Denne informasjonen vises kun i forhåndsvisningen

Følgende betingelser må være oppfylt for at spørsmålet skal vises for respondenten:

Dersom spørsmålet Har du sett liknande förändringar som Bild 2. A-B visar på dina renar under de senaste 10 åren? inneholder noen av disse alternativene

- Vet ikke
- Ja

Smittsom munnskurv (orf)

39) Omtrent hvor mange reinsdyr ble berørt i gruppen der det sist var utbrudd, alternativt tilfeller av smittsom munnskurv (orf)? Oppgi i første omgang antallet ved utbrudd om du har opplevd det.

Antall kalv (yngre enn 1 år)

Antall unge dyr (1-3 år)

Antall voksne (eldre enn 3 år)

Totalt antall reinsdyr i den berørte gruppen på tidspunktet for utbruddet (både friske og syke)

Denne informasjonen vises kun i forhåndsvisningen

Følgende betingelser må være oppfylt for at spørsmålet skal vises for respondenten:

Dersom spørsmålet Har du sett liknande förändringar som Bild 2. A-B visar på dina renar under de senaste 10 åren? inneholder noen av disse alternativene

- Vet ikke
- Ja

40) Plass for kommentarer:

Denne informasjonen vises kun i forhåndsvisningen

Følgende betingelser må være oppfylt for at spørsmålet skal vises for respondenten:

Dersom spørsmålet Har du sett liknande förändringar som Bild 2. A-B visar på dina renar under de senaste 10 åren? inneholder noen av disse alternativene

- Vet ikke
- Ja

Smittsom munnskurv (orf)

41) * Opplever du at antallet reinsdyr som berøres av smittsom munnskurv (orf) har endret seg de siste 5 årene?

- ○ Ja, forekomsten har økt
- ○ Ja, avtagende forekomst
- ○ Nei, sykdomsfrekvensen er uendret

◯ Vet ikke

Denne informasjonen vises kun i forhåndsvisningen

Følgende betingelser må være oppfylt for at spørsmålet skal vises for respondenten:

Dersom spørsmålet Har du sett liknande förändringar som Bild 2. A-B visar på dina renar under de senaste 10 åren? inneholder noen av disse alternativene

- Vet ikke
- Ja

og

Dersom spørsmålet Upplever du att antalet renar som drabbats av munvårtsjuka/orf har förändrats över de senaste 5 åren? inneholder noen av disse alternativene

- Ja, forekomsten har økt

Smittsom munnskurv (orf)

42) Hva tror du denne økningen i antall berørte reinsdyr kan skyldes?

Denne informasjonen vises kun i forhåndsvisningen

Følgende betingelser må være oppfylt for at spørsmålet skal vises for respondenten:

Dersom spørsmålet Har du sett liknande förändringar som Bild 2. A-B visar på dina renar under de senaste 10 åren? inneholder noen av disse alternativene

- Vet ikke
- Ja

og

Dersom spørsmålet Upplever du att antalet renar som drabbats av munvårtsjuka/orf har förändrats över de senaste 5 åren? inneholder noen av disse alternativene

- Ja, avtagende forekomst

Smittsom munnskurv (orf)

43) Hva tror du denne avtagende forekomst kan skyldes?

Denne informasjonen vises kun i forhåndsvisningen

Følgende betingelser må være oppfylt for at spørsmålet skal vises for respondenten:

Dersom spørsmålet Har du sett liknande förändringar som Bild 2. A-B visar på dina renar under de senaste 10 åren? inneholder noen av disse alternativene

- Vet ikke
- Ja

Smittsom munnskurv (orf)

44) * Gjøres det vanligvis tiltak (f.eks. gruppering, slakting eller annen håndtering) når du ser reinsdyr berørt av smittsom munnskurv (orf)?

- ○ Ja
- ○ Nei
- ○ Vet ikke

Denne informasjonen vises kun i forhåndsvisningen

Følgende betingelser må være oppfylt for at spørsmålet skal vises for respondenten:

Dersom spørsmålet Har du sett liknande förändringar som Bild 2. A-B visar på dina renar under de senaste 10 åren? inneholder noen av disse alternativene

- Vet ikke
- Ja

45) Hvilke tiltak ble iverksatt da du sist så reinsdyr berørt av smittsom munnskurv (orf)? Oppgi alle tiltak som ble satt i verk.

- ☐ Ingen tiltak
- ☐ Gruppering av berørte dyr i eget gjerde for syke dyr
- ☐ Veterinær ble kontaktet
- ☐ Antibiotikabehandling
- ☐ Slakt
- ☐ Avliving/kassering
- ☐ Behandling med andre legemidler, oppgi i kommentarfeltet under
- ☐ Annet, forklar i kommentarfeltet under

Denne informasjonen vises kun i forhåndsvisningen

Følgende betingelser må være oppfylt for at spørsmålet skal vises for respondenten:

Dersom spørsmålet Har du sett liknande förändringar som Bild 2. A-B visar på dina renar under de senaste 10 åren? inneholder noen av disse alternativene

- Vet ikke
- Ja

46) Plass for kommentarer:

Denne informasjonen vises kun i forhåndsvisningen

Følgende betingelser må være oppfylt for at spørsmålet skal vises for respondenten:

Dersom spørsmålet Har du sett liknande förändringar som Bild 2. A-B visar på dina renar under de senaste 10 åren? inneholder noen av disse alternativene

- Vet ikke
- Ja

Smittsom munnskurv (orf)

47) Har utbrudd eller tilfeller av smittsom munnskurv (orf) ført til økonomiske konsekvenser?

- ○ Ja
- ○ Nei
- ○ Vet ikke

Denne informasjonen vises kun i forhåndsvisningen

Følgende betingelser må være oppfylt for at spørsmålet skal vises for respondenten:

Dersom spørsmålet Har du sett liknande förändringar som Bild 2. A-B visar på dina renar under de senaste 10 åren? inneholder noen av disse alternativene

- Vet ikke
- Ja

48) Plass for kommentarer:

Denne informasjonen vises kun i forhåndsvisningen

Følgende betingelser må være oppfylt for at spørsmålet skal vises for respondenten:

Dersom spørsmålet Har du sett liknande förändringar som Bild 2. A-B visar på dina renar under de senaste 10 åren? inneholder noen av disse alternativene

- Vet ikke
- Ja

Smittsom munnskurv (orf)

Bilde 2. A-B:

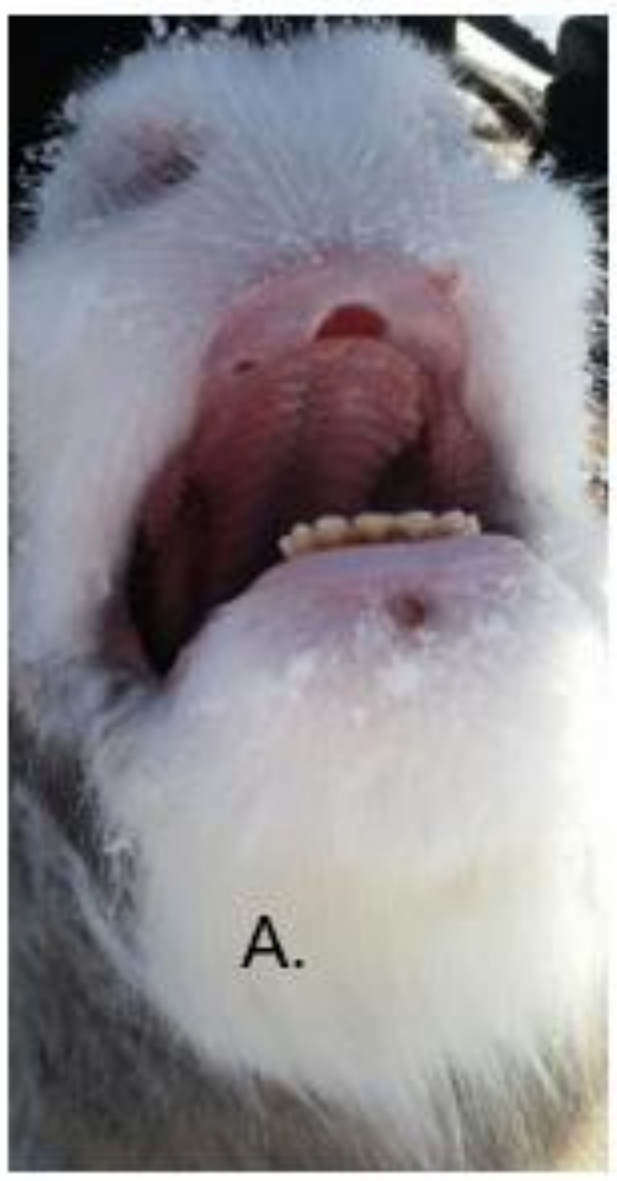

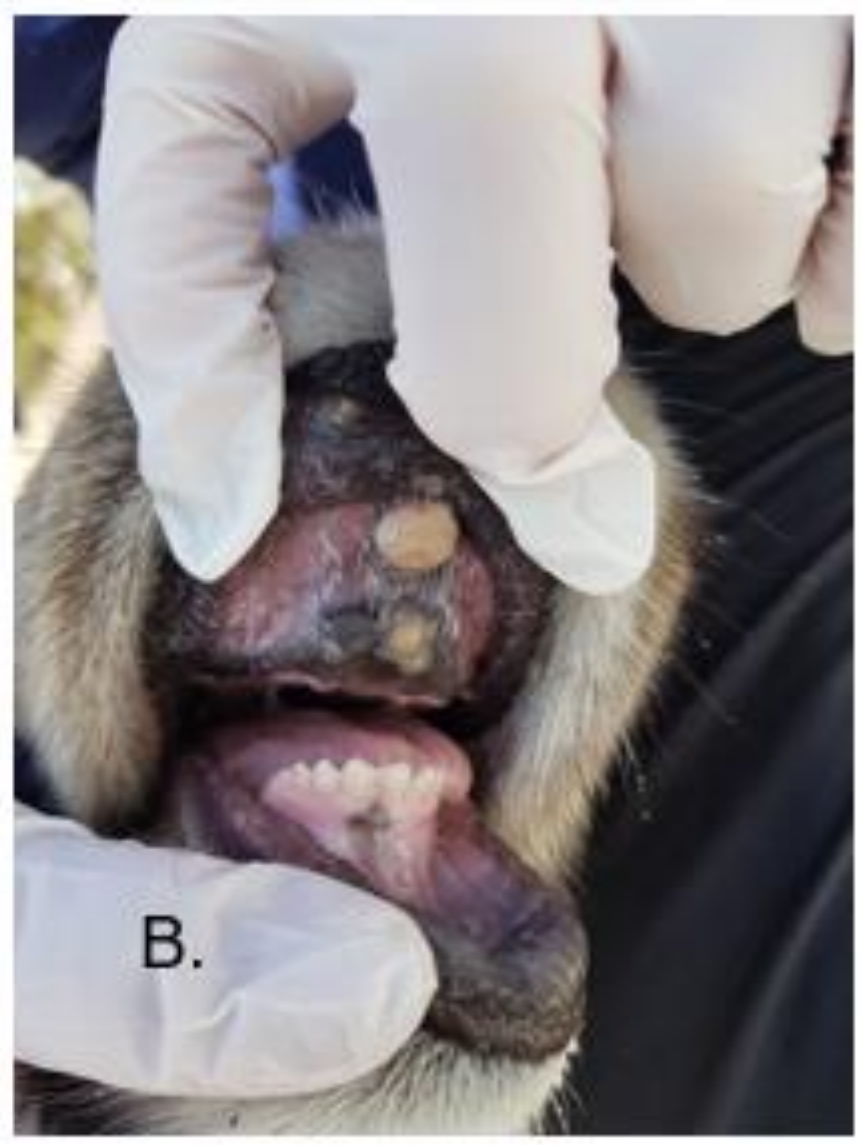

49) Har noen av forandringene på bildene et tradisjonelt navn for deg eller for Siida enheten?

- ○ Ja, oppgi i kommentarfeltet under
- ○ Nei
- ○ Vet ikke

Denne informasjonen vises kun i forhåndsvisningen

Følgende betingelser må være oppfylt for at spørsmålet skal vises for respondenten:

Dersom spørsmålet Har du sett liknande förändringar som Bild 2. A-B visar på dina renar under de senaste 10 åren? inneholder noen av disse alternativene

- Vet ikke
- Ja

50) Plass for kommentarer/oppgi tradisjonelt navn:

Denne informasjonen vises kun i forhåndsvisningen

Følgende betingelser må være oppfylt for at spørsmålet skal vises for respondenten:

Dersom spørsmålet Har du sett liknande förändringar som Bild 2. A-B visar på dina renar under de senaste 10 åren? inneholder noen av disse alternativene

- Vet ikke
- Ja

51) Kjenner du til noen tradisjonelle behandlinger av smittsom munnskurv (orf)?

- Ja, beskriv i kommentarfeltet under
- Nei
- Vet ikke

Denne informasjonen vises kun i forhåndsvisningen

Følgende betingelser må være oppfylt for at spørsmålet skal vises for respondenten:

Dersom spørsmålet Har du sett liknande förändringar som Bild 2. A-B visar på dina renar under de senaste 10 åren? inneholder noen av disse alternativene

- Vet ikke
- Ja

52) Plass for kommentarer:

Denne informasjonen vises kun i forhåndsvisningen

Følgende betingelser må være oppfylt for at spørsmålet skal vises for respondenten:

Dersom spørsmålet Har du sett liknande förändringar som Bild 2. A-B visar på dina renar under de senaste 10 åren? inneholder noen av disse alternativene

- Vet ikke
- Nei
- Ja

Straks følger spørsmål om munnråte / oral nekrobacillose / njunnevikke

53) Legg gjerne igjen andre kommentarer relatert til smittsom munnskurv (orf) her:

3. Spørsmål om munnråte/oral nekrobacillose/njunnevikke:

Bilde 3. A-C:

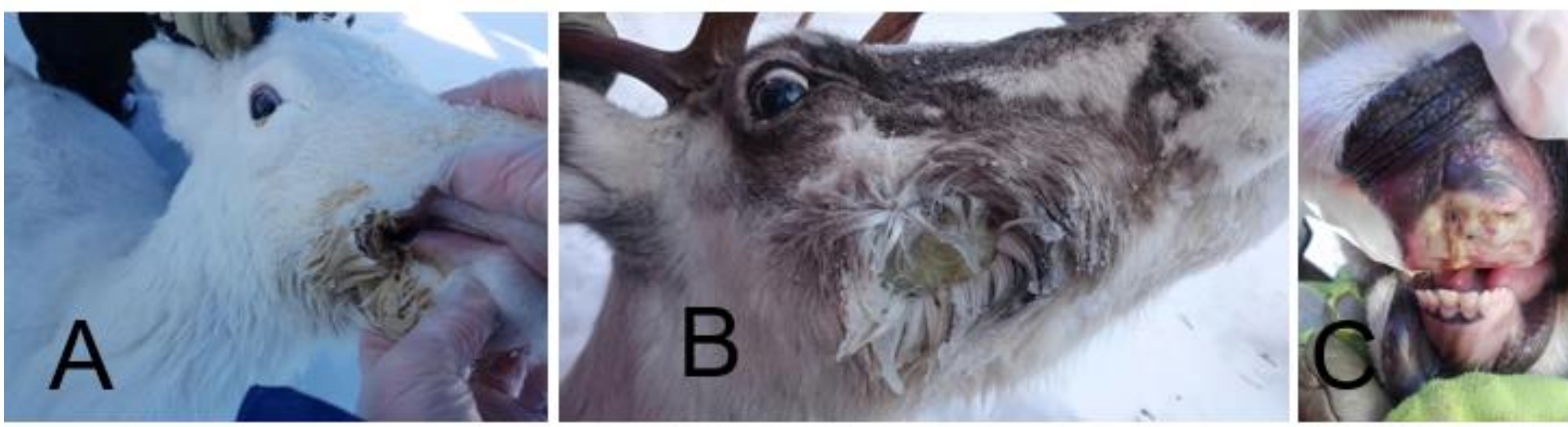

A: Sår og tilgriset munnvik med pussdannelse.

B: Infeksjon i kinnet der det har blitt et permanent hull i kinnet.

C: Infeksjon i tannkjøttet med pussdannelse.

54) * Har du sett lignende forandringer som bilde 3. A-C viser, på dine reinsdyr de siste 10 årene?

- ○ Ja
- ○ Nei
- ○ Vet ikke

Denne informasjonen vises kun i forhåndsvisningen

Følgende betingelser må være oppfylt for at spørsmålet skal vises for respondenten:

Dersom spørsmålet Har du sett liknande förändringar som Bild 3. A-C visar på dina renar under de senaste 10 åren? inneholder noen av disse alternativene

- Vet ikke
- Ja

Munnråte/oral nekrobacillose/njunnevikke

Bilde 3. A-C:

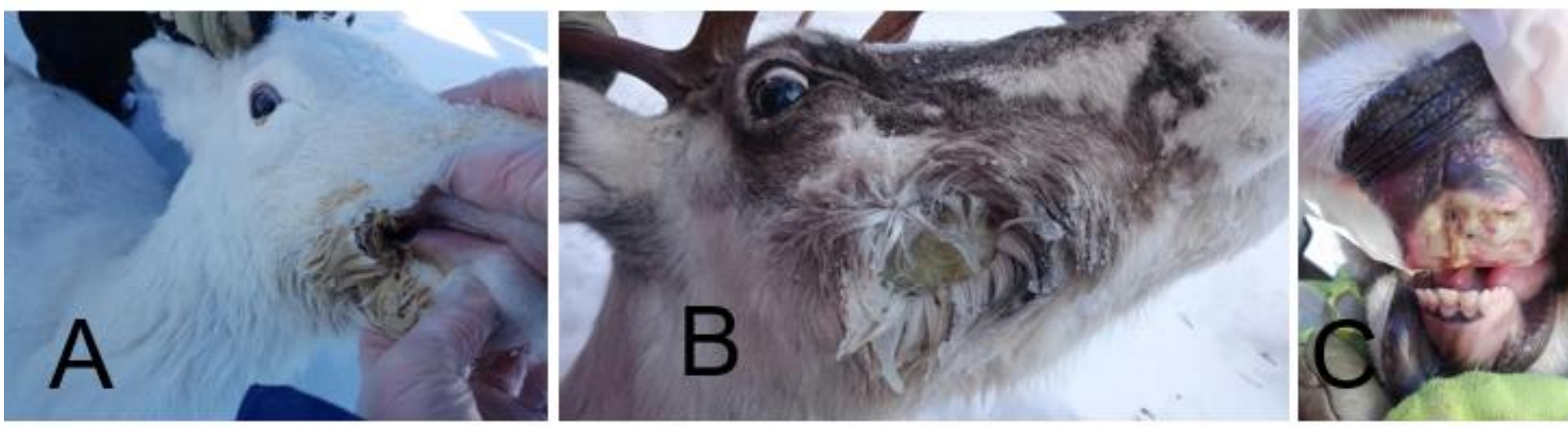

55) Når så du sist lignende forandringer som vist på bilde 3. A-C?

- ○ Siste året
- ○ Ikke det siste året, men for mindre enn 5 år siden
- ○ Mer enn 5 år siden
- ○ Vet ikke

Denne informasjonen vises kun i forhåndsvisningen

Følgende betingelser må være oppfylt for at spørsmålet skal vises for respondenten:

Dersom spørsmålet Har du sett liknande förändringar som Bild 3. A-C visar på dina renar under de senaste 10 åren? inneholder noen av disse alternativene

- Vet ikke
- Ja

Munnråte/oral nekrobacillose/njunnevikke

56) Når på året ser du flest antall reinsdyr med munnråte/oral nekrobacillose/njunnevikke? Oppgi for kalv, unge dyr og voksne dyr. Det er mulig å velge flere sesonger.

	Ikke observert	Vår	Sommer	Høst	Vinter	Året rundt (oppgi som eneste svarsalternativ!)
Kalv (yngre enn 1 år)	☐	☐	☐	☐	☐	☐
Unge dyr (1-3 år)	☐	☐	☐	☐	☐	☐
Voksen (over 3 år)	☐	☐	☐	☐	☐	☐

Denne informasjonen vises kun i forhåndsvisningen

Følgende betingelser må være oppfylt for at spørsmålet skal vises for respondenten:

Dersom spørsmålet Har du sett liknande förändringar som Bild 3. A-C visar på dina renar under de senaste 10 åren? inneholder noen av disse alternativene

- Vet ikke
- Ja

57) Plass for kommentarer:

Denne informasjonen vises kun i forhåndsvisningen

Følgende betingelser må være oppfylt for at spørsmålet skal vises for respondenten:

Dersom spørsmålet Har du sett liknande förändringar som Bild 3. A-C visar på dina renar under de senaste 10 åren? inneholder noen av disse alternativene

- Vet ikke
- Ja

Munnråte/oral nekrobacillose/njunnevikke

58) Oppgi, for hver av sesongene nedenfor, hvor reinen befant seg under utbrudd av munnråte/oral nekrobacillose/njunnevikke. Alternativt at ingen utbrudd fant sted. Utbrudd defineres som en betydelig økning i antall tilfeller over en viss tidsperiode, for eksempel flere berørte dyr enn vanlig i løpet av en sesong, eller over en kortere tidsperiode, for eksempel noen få uker.

	Ikke opplevd utbrudd	Utbrudd i innhegningen	Utbrudd på fribeite med fôring	Utbrudd på fribeite uten fôring
2015/2016	☐	☐	☐	☐
2016/2017	☐	☐	☐	☐
2017/2018	☐	☐	☐	☐
2018/2019	☐	☐	☐	☐
2019/2020	☐	☐	☐	☐
2020/2021	☐	☐	☐	☐

Denne informasjonen vises kun i forhåndsvisningen

Følgende betingelser må være oppfylt for at spørsmålet skal vises for respondenten:

Dersom spørsmålet Har du sett liknande förändringar som Bild 3. A-C visar på dina renar under de senaste 10 åren? inneholder noen av disse alternativene

- Vet ikke
- Ja

59) Plass for kommentarer:

Denne informasjonen vises kun i forhåndsvisningen

Følgende betingelser må være oppfylt for at spørsmålet skal vises for respondenten:

Dersom spørsmålet Har du sett liknande förändringar som Bild 3. A-C visar på dina renar under de senaste 10 åren? inneholder noen av disse alternativene

- Vet ikke
- Ja

Munnråte/oral nekrobacillose/njunnevikke

60) Omtrent hvor mange reinsdyr ble berørt i gruppen der det sist var utbrudd, alternativt tilfeller av munnråte/oral nekrobacillose/njunnevikke? Oppgi i første omgang antallet ved utbrudd om du har opplevd det.

Antall kalv (yngre enn 1 år)

Antall unge dyr (1-3 år)

Antall voksne (eldre enn 3 år)

Totalt antall reinsdyr i den berørte gruppen på tidspunktet for utbruddet (både friske og syke)

Denne informasjonen vises kun i forhåndsvisningen

Følgende betingelser må være oppfylt for at spørsmålet skal vises for respondenten:

Dersom spørsmålet Har du sett liknande förändringar som Bild 3. A-C visar på dina renar under de senaste 10 åren? inneholder noen av disse alternativene

- Vet ikke
- Ja

61) Plass for kommentarer:

Denne informasjonen vises kun i forhåndsvisningen

Følgende betingelser må være oppfylt for at spørsmålet skal vises for respondenten:

Dersom spørsmålet Har du sett liknande förändringar som Bild 3. A-C visar på dina renar under de senaste 10 åren? inneholder noen av disse alternativene

- Vet ikke
- Ja

Munnråte/oral nekrobacillose/njunnevikke

62) * Opplever du at antallet reinsdyr som berøres av munnråte/oral nekrobacillose/njunnevikke har endret seg de siste 5 årene?

- ○ Ja, forekomsten har økt
- ○ Ja, avtagende forekomst
- ○ Nei, sykdomsfrekvensen er uendret

○ Vet ikke

Denne informasjonen vises kun i forhåndsvisningen

Følgende betingelser må være oppfylt for at spørsmålet skal vises for respondenten:

Dersom spørsmålet Har du sett liknande förändringar som Bild 3. A-C visar på dina renar under de senaste 10 åren? inneholder noen av disse alternativene

- Vet ikke
- Ja

og

Dersom spørsmålet Upplever du att antalet renar som drabbats av munröta/oral nekrobacillos/njunnevikke har förändrats över de senaste fem åren? inneholder noen av disse alternativene

- Ja, forekomsten har økt

Munnråte/oral nekrobacillose/njunnevikke

63) Hva tror du denne økningen i antall berørte reinsdyr kan skyldes?

Denne informasjonen vises kun i forhåndsvisningen

Følgende betingelser må være oppfylt for at spørsmålet skal vises for respondenten:

Dersom spørsmålet Har du sett liknande förändringar som Bild 3. A-C visar på dina renar under de senaste 10 åren? inneholder noen av disse alternativene

- Vet ikke
- Ja

og

Dersom spørsmålet Upplever du att antalet renar som drabbats av munröta/oral nekrobacillos/njunnevikke har förändrats över de senaste fem åren? inneholder noen av disse alternativene

- Ja, avtagende forekomst

Munnråte/oral nekrobacillose/njunnevikke

64) Hva tror du denne avtagende forekomst kan skyldes?

Denne informasjonen vises kun i forhåndsvisningen

Følgende betingelser må være oppfylt for at spørsmålet skal vises for respondenten:

Dersom spørsmålet Har du sett liknande förändringar som Bild 3. A-C visar på dina renar under de senaste 10 åren? inneholder noen av disse alternativene

- Vet ikke
- Ja

Munnråte/oral nekrobacillose/njunnevikke

65) * Gjøres det vanligvis tiltak (f.eks. gruppering, slakting eller annen håndtering) når du ser reinsdyr berørt av munnråte/oral nekrobacillose/njunnevikke?

- ○ Ja
- ○ Nei
- ○ Vet ikke

Denne informasjonen vises kun i forhåndsvisningen

Følgende betingelser må være oppfylt for at spørsmålet skal vises for respondenten:

Dersom spørsmålet Har du sett liknande förändringar som Bild 3. A-C visar på dina renar under de senaste 10 åren? inneholder noen av disse alternativene

- Vet ikke
- Ja

66) Hvilke tiltak ble iverksatt da du sist så reinsdyr berørt av munnråte/oral nekrobacillose/njunnevikke? Oppgi alle tiltak som ble satt i verk.

- ☐ Ingen tiltak
- ☐ Gruppering av berørte dyr i eget gjerde for syke dyr
- ☐ Veterinær ble kontaktet
- ☐ Antibiotikabehandling
- ☐ Slakt
- ☐ Avliving/kassering
- ☐ Behandling med andre legemidler, oppgi i kommentarfeltet under
- ☐ Annet, forklar i kommentarfeltet under

Denne informasjonen vises kun i forhåndsvisningen

Følgende betingelser må være oppfylt for at spørsmålet skal vises for respondenten:

Dersom spørsmålet Har du sett liknande förändringar som Bild 3. A-C visar på dina renar under de senaste 10 åren? inneholder noen av disse alternativene

- Vet ikke
- Ja

67) Plass for kommentarer:

Denne informasjonen vises kun i forhåndsvisningen

Følgende betingelser må være oppfylt for at spørsmålet skal vises for respondenten:

Dersom spørsmålet Har du sett liknande förändringar som Bild 3. A-C visar på dina renar under de senaste 10 åren? inneholder noen av disse alternativene

- Vet ikke
- Ja

Munnråte/oral nekrobacillose/njunnevikke

68) Har utbrudd eller tilfeller av munnråte/oral nekrobacillose/njunnevikke ført til økonomiske konsekvenser?

- ○ Ja
- ○ Nei
- ○ Vet ikke

Denne informasjonen vises kun i forhåndsvisningen

Følgende betingelser må være oppfylt for at spørsmålet skal vises for respondenten:

Dersom spørsmålet Har du sett liknande förändringar som Bild 3. A-C visar på dina renar under de senaste 10 åren? inneholder noen av disse alternativene

- Vet ikke
- Ja

69) Plass for kommentarer:

Denne informasjonen vises kun i forhåndsvisningen

Følgende betingelser må være oppfylt for at spørsmålet skal vises for respondenten:

Dersom spørsmålet Har du sett liknande förändringar som Bild 3. A-C visar på dina renar under de senaste 10 åren? inneholder noen av disse alternativene

- Vet ikke
- Ja

Munnråte/oral nekrobacillose/njunnevikke

Bilde 3. A-C:

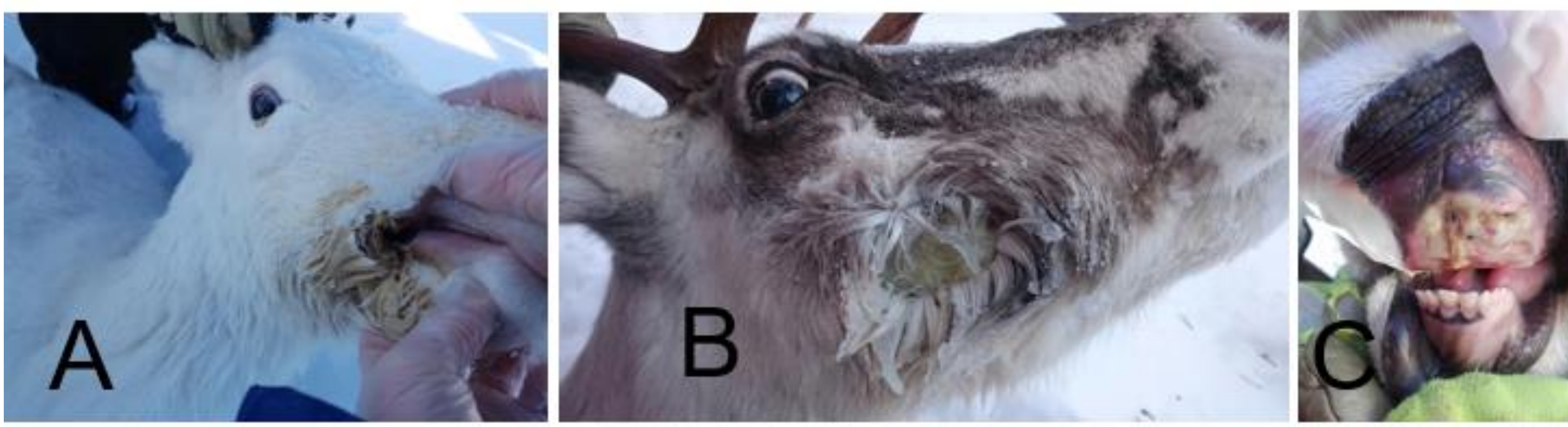

70) Har noen av forandringene, som vist på bildene over, et tradisjonelt navn for deg eller for Siida enheten?

- Ja, oppgi i kommentarfeltet under
- Nei
- Vet ikke

Denne informasjonen vises kun i forhåndsvisningen

Følgende betingelser må være oppfylt for at spørsmålet skal vises for respondenten:

Dersom spørsmålet Har du sett liknande förändringar som Bild 3. A-C visar på dina renar under de senaste 10 åren? inneholder noen av disse alternativene

- Vet ikke
- Ja

71) Plass for kommentarer/oppgi tradisjonelt navn:

Denne informasjonen vises kun i forhåndsvisningen

Følgende betingelser må være oppfylt for at spørsmålet skal vises for respondenten:

Dersom spørsmålet Har du sett liknande förändringar som Bild 3. A-C visar på dina renar under de senaste 10 åren? inneholder noen av disse alternativene

- Vet ikke
- Ja

72) Kjenner du til noen tradisjonelle behandlinger av munnråte/oral nekrobacillose/njunnevikke?

- ○ Ja, beskriv i kommentarfeltet under
- ○ Nei
- ○ Vet ikke

Denne informasjonen vises kun i forhåndsvisningen

Følgende betingelser må være oppfylt for at spørsmålet skal vises for respondenten:

Dersom spørsmålet Har du sett liknande förändringar som Bild 3. A-C visar på dina renar under de senaste 10 åren? inneholder noen av disse alternativene

- Vet ikke
- Ja

73) Plass for kommentarer:

Denne informasjonen vises kun i forhåndsvisningen

Følgende betingelser må være oppfylt for at spørsmålet skal vises for respondenten:

Dersom spørsmålet Har du sett liknande förändringar som Bild 3. A-C visar på dina renar under de senaste 10 åren? inneholder noen av disse alternativene

- Vet ikke
- Nei
- Ja

Nekrobacillose i magene på rein:

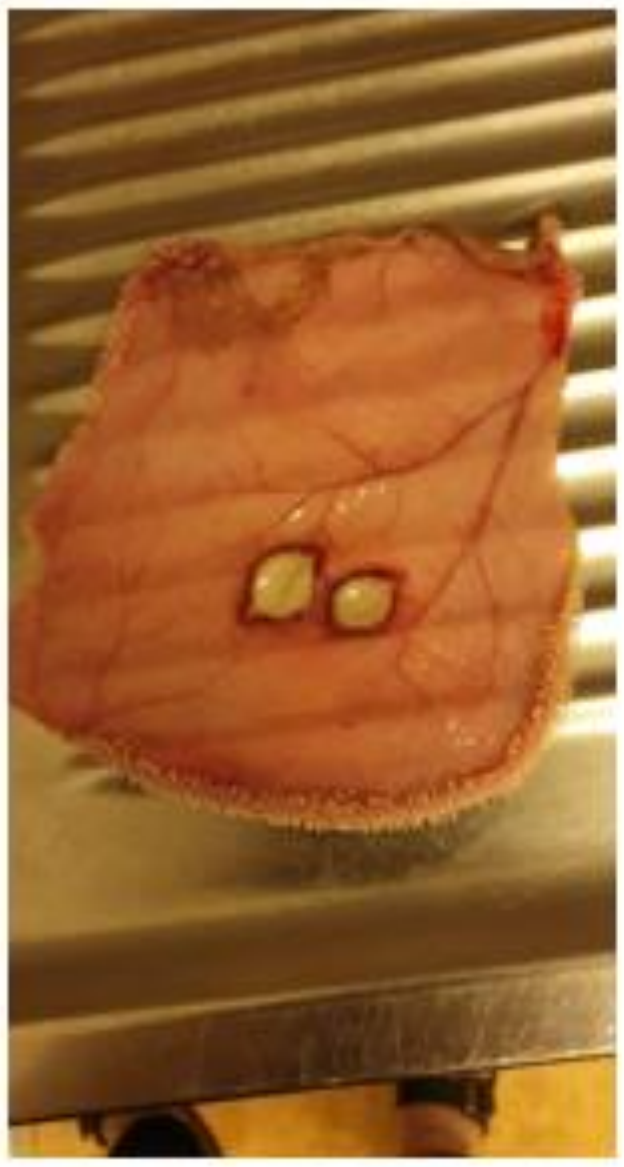 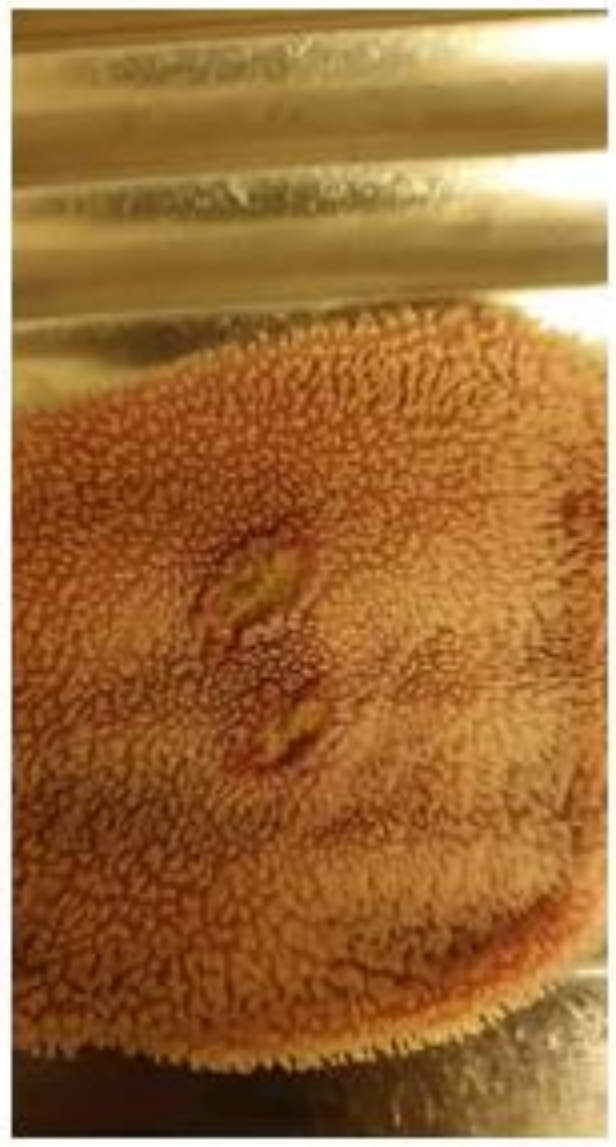

74) Har du sett lignende forandringer, som vist på bildene over, i magen på dine reinsdyr (ved slakt / obduksjon)?

- Ja
- Nei
- Vet ikke

Denne informasjonen vises kun i forhåndsvisningen

Følgende betingelser må være oppfylt for at spørsmålet skal vises for respondenten:

Dersom spørsmålet Har du sett liknande förändringar som Bild 3. A-C visar på dina renar under de senaste 10 åren? inneholder noen av disse alternativene

- Vet ikke
- Nei
- Ja

Straks følger spørsmål om andre sykdommer hos rein

75) Legg gjerne igjen andre kommentarer relatert til munnråte/oral nekrobacillose/njunnevikke her:

4. Spørsmål om andre sykdommer hos reinsdyr:

76) * Hvilke sykdommer har du observert fra vintersesongen 2019/2020 frem til idag? Oppgi alle alternativ som stemmer.

- ☐ Kasting av foster
- ☐ Livmorprolaps (livmorframfall - at livmor vrenges ut av dyret)
- ☐ Ustøhet/halthet
- ☐ Avmagring
- ☐ Bløt buk
- ☐ Diaré
- ☐ Skvalpmage (løst, vandig innhold i mageavsnitt)
- ☐ Trommesjuke (oppblåst buk)
- ☐ Hjernemark
- ☐ Parasitter (hudbrems, svelgbrems, innvollsparasitter, hud/pels-parasitter)
- ☐ Selvdøde dyr uten kjent dødsårsak
- ☐ Annet, oppgi i kommentarfeltet under
- ☐ Ingen av sykdommene over

77) Plass for kommentarer:

Denne informasjonen vises kun i forhåndsvisningen

Følgende betingelser må være oppfylt for at spørsmålet skal vises for respondenten:

Dersom spørsmålet Vilka av följande sjukdomar har du observerat från vintersäsongen 2019/2020 fram till idag? Ange alla som observerats. inneholder noen av disse alternativene

- Bløt buk

78) Hva ble berørte dyr fôret med før sykdommen 'bløt buk' brøt ut?

- Kun pellets
- Kun silofôr/surfôr
- Kombinasjon pellets og silofôr/surfôr
- Ikke fôret
- Annet: oppgi i kommentarfeltet under

Denne informasjonen vises kun i forhåndsvisningen

Følgende betingelser må være oppfylt for at spørsmålet skal vises for respondenten:

Dersom spørsmålet Vilka av följande sjukdomar har du observerat från vintersäsongen 2019/2020 fram till idag? Ange alla som observerats. inneholder noen av disse alternativene

- Bløt buk

79) Plass for kommentarer:

Denne informasjonen vises kun i forhåndsvisningen

Følgende betingelser må være oppfylt for at spørsmålet skal vises for respondenten:

Dersom spørsmålet Vilka av följande sjukdomar har du observerat från vintersäsongen 2019/2020 fram till idag? Ange alla som observerats. inneholder noen av disse alternativene

- Bløt buk

80) Har du observert noen andre/flere symptomer på reinsdyr som er berørt av bløt buk? Beskriv.

Denne informasjonen vises kun i forhåndsvisningen

Følgende betingelser må være oppfylt for at spørsmålet skal vises for respondenten:

81) Får du den hjelpen du har behov av fra veterinær?

- Ja
- Nei, oppgi årsaken i fritekst:

Denne informasjonen vises kun i forhåndsvisningen

Følgende betingelser må være oppfylt for at spørsmålet skal vises for respondenten:

82) Plass for kommentarer:

83) Har du sendt noen reinsdyr til obduksjon?

- Ja
- Nei

84) Har du selv obdusert noen reinsdyr med veterinærhjelp?

- Ja
- Nei

85) Plass for kommentarer:

86) * Behandles reinsdyrene regelmessig mot hud- og/eller svelgbrems?

- Ja
- Nei

Denne informasjonen vises kun i forhåndsvisningen

Følgende betingelser må være oppfylt for at spørsmålet skal vises for respondenten:

Dersom spørsmålet Behandlas renarna regelbundet mot Korm? inneholder noen av disse alternativene

- Ja

87) Under hvilken/hvilke sesonger utføres vanligvis behandlingen mot hud- og/eller svelgbrems?

- ☐ Vår
- ☐ Sommer
- ☐ Høst
- ☐ Vinter

Denne informasjonen vises kun i forhåndsvisningen

Følgende betingelser må være oppfylt for at spørsmålet skal vises for respondenten:

Dersom spørsmålet Behandlas renarna regelbundet mot Korm? inneholder noen av disse alternativene

- Ja

88) Hvilken gruppe av dyr behandles vanligvis mot hud- og/eller svelgbrems?

- Kun livkalver (1 år eller yngre)
- Kun simler (over 1 år)
- Kun bukker (over 1 år)
- Kombinasjon, livkalver, bukker eller simler
- Annet, oppgi i kommentarfeltet under.

Denne informasjonen vises kun i forhåndsvisningen

Følgende betingelser må være oppfylt for at spørsmålet skal vises for respondenten:

Dersom spørsmålet Behandlas renarna regelbundet mot Korm? inneholder noen av disse alternativene

- Ja

89) Plass for kommentarer:

Bild 4. A-B. Flått

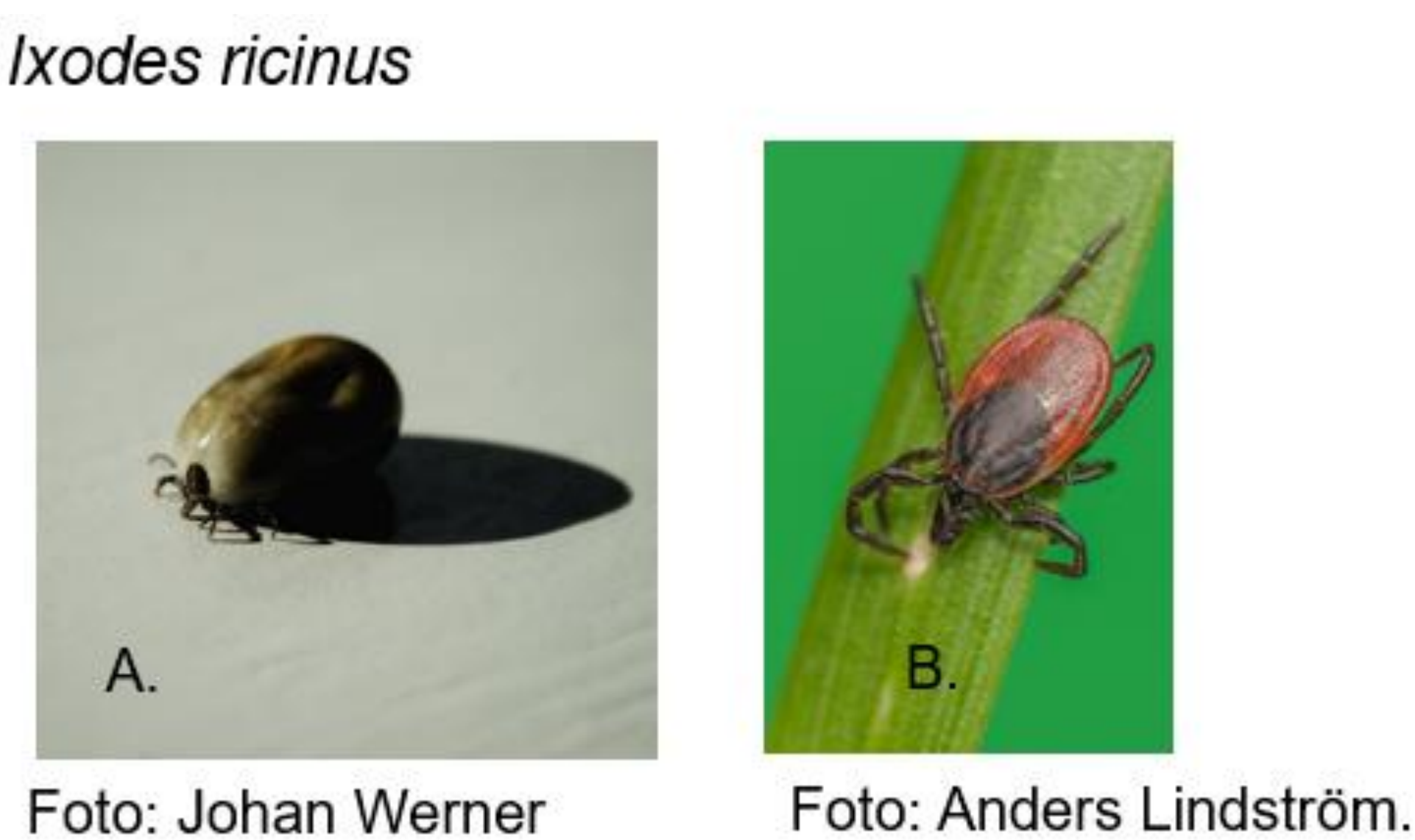

A: Blodfylt hunnflått.

B: Hunnflått på et gresstrå.

90) Flått kan spre smittsomme sykdommer og på grunn av mildere klima, sprer flåtten seg nordover. Har du sett flått på reinsdyrene dine (se bilde 4. A-B)?

- ○ Ja
- ○ Nei
- ○ Vet ikke

Straks følger spørsmål om fôring

91) Legg gjerne igjen andre kommentarer relatert til andre sykdommer hos rein her:

5. Nå har du bare spørsmål om fôring igjen før undersøkelsen er fullført!

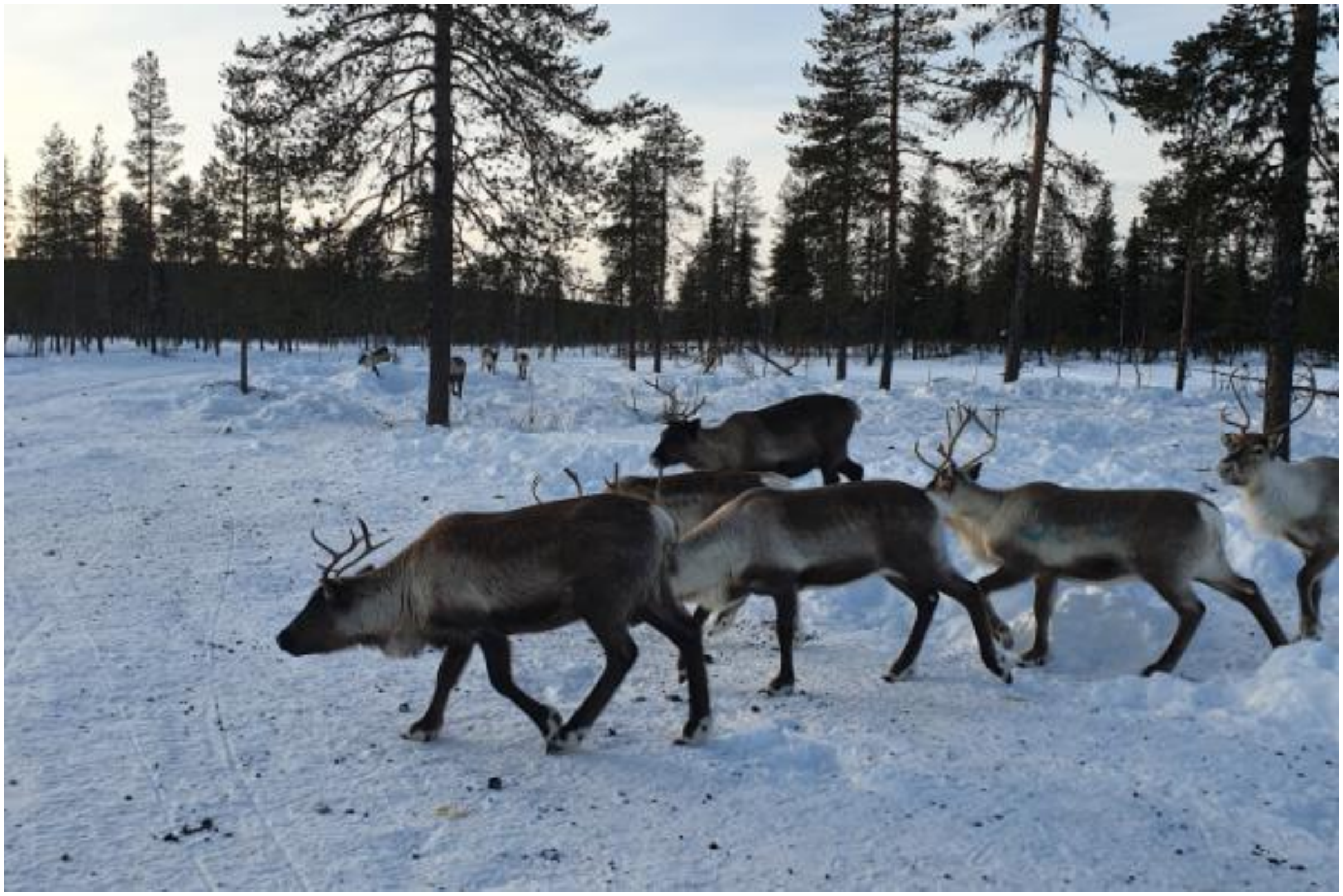

Spørsmål om fôring er delt inn i følgende:

1. Generell del

2. Effekter av fôring

3. Fôringsrutiner (gjelder 2019/2020)

4. Annet (gjelder de siste fem årene)

92) * Har du fôret (nød- eller tilleggsfôret) reinsdyrene dine i din vintergruppe ved noen tilfeller de siste 5 årene? Hvis Siida enheten ikke er delt inn i flere vintergrupper, svarer du for hele Siida enheten. Obs: dette refererer til fôring av vintergruppe i innhegning og / eller på fribeite i over to uker, men ikke under flytting og samling som varer under en kortere periode enn to uker.

- Ja
- Nei

Denne informasjonen vises kun i forhåndsvisningen

Følgende betingelser må være oppfylt for at spørsmålet skal vises for respondenten:

Dersom spørsmålet Har du utfodrat (nöd- eller stödutfodrat) dina renar i din vintergruppen vid något tillfälle under de senaste fem åren? Om samebyn inte är uppdelad i olika vintergrupper svarar du för hela samebyn. Obs: här avses utfodring av vintergrupp (slakt- och livren) i hägn och/eller på fribete i över två veckor, men ej under flytt och samling som pågår under en kortare period än två veckor. inneholder noen av disse alternativene

- Ja

FÔRING

1. Generell del

Dette refererer til fôring (nød- eller tilleggsfôring) av reinsdyrene dine (slakterein og livrein) i vintergruppen i over to uker, i innhenging eller på fribeite, men ikke under flytting og samling som varer en kortere periode enn to uker. Om vintergruppen ikke er delt, svarer du for hele Siida enheten.

93) * Oppgi under hver av følgende sesonger, hvor reinsdyrene ble fôret, alternativt at de ikke ble fôret i det hele tatt. Svar for hver sesong, ett / flere alternativ.

	Ikke fôret	Fôret i innhegningen	Fôret på fribeite	Vet ikke
2015/2016	☐	☐	☐	☐
2016/2017	☐	☐	☐	☐
2017/2018	☐	☐	☐	☐
2018/2019	☐	☐	☐	☐
2019/2020	☐	☐	☐	☐
2020/2021	☐	☐	☐	☐

Denne informasjonen vises kun i forhåndsvisningen

Følgende betingelser må være oppfylt for at spørsmålet skal vises for respondenten:

Dersom spørsmålet Har du utfodrat (nöd- eller stödutfodrat) dina renar i din vintergruppen vid något tillfälle under de senaste fem åren? Om samebyn inte är uppdelad i olika vintergrupper svarar du för hela samebyn. Obs: här avses utfodring av vintergrupp (slakt- och livren) i hägn och/eller på fribete i över två veckor, men ej under flytt och samling som pågår under en kortare period än två veckor. inneholder noen av disse alternativene

- Ja

94) Plass for kommentarer:

Denne informasjonen vises kun i forhåndsvisningen

Følgende betingelser må være oppfylt for at spørsmålet skal vises for respondenten:

Dersom spørsmålet Har du utfodrat (nöd- eller stödutfodrat) dina renar i din vintergruppen vid något tillfälle under de senaste fem åren? Om samebyn inte är uppdelad i olika vintergrupper svarar du för hela samebyn. Obs: här avses utfodring av vintergrupp (slakt- och livren) i hägn och/eller på fribete i över två veckor, men ej under flytt och samling som pågår under en kortare period än två veckor. inneholder noen av disse alternativene

- Ja

FÔRING

1. Generell del

Dette refererer til fôring (nød- eller tilleggsfôring) av reinsdyrene dine (slakterein og livrein) i vintergruppen i over to uker, i innhenging eller på fribeite, men ikke under flytting og samling som varer en kortere periode enn to uker. Om vintergruppen ikke er delt, svarer du for hele Siida enheten.

95) Hvorfor ble reinsdyrene vinterfôret de siste fem årene? Oppgi alle alternativ som stemmer for livrein og for slaktrein. Kommenter eventuelle forskjeller mellom årene i kommentarfeltet nedenfor.

	Livrein	Slakterein
Dårlige vinterbeiteforhold	☐	☐
Overlevelse	☐	☐
Bedre vekst	☐	☐
Rovdyr	☐	☐
Sykdom	☐	☐
Redusere cesiuminnhold (Tsjernobylulykken)	☐	☐

	Livrein	Slakterein
Konkurrerende arealbruk. Spesifiser, hvis mulig i kommentarfeltet under	☐	☐
Annet, oppgi i kommentarfeltet under	☐	☐

Denne informasjonen vises kun i forhåndsvisningen

Følgende betingelser må være oppfylt for at spørsmålet skal vises for respondenten:

Dersom spørsmålet Har du utfodrat (nöd- eller stödutfodrat) dina renar i din vintergruppen vid något tillfälle under de senaste fem åren? Om samebyn inte är uppdelad i olika vintergrupper svarar du för hela samebyn. Obs: här avses utfodring av vintergrupp (slakt- och livren) i hägn och/eller på fribete i över två veckor, men ej under flytt och samling som pågår under en kortare period än två veckor. inneholder noen av disse alternativene

- Ja

96) Plass for kommentarer, oppgi om det gjelder for slakterein eller livrein:

Denne informasjonen vises kun i forhåndsvisningen

Følgende betingelser må være oppfylt for at spørsmålet skal vises for respondenten:

Dersom spørsmålet Har du utfodrat (nöd- eller stödutfodrat) dina renar i din vintergruppen vid något tillfälle under de senaste fem åren? Om samebyn inte är uppdelad i olika vintergrupper svarar du för hela samebyn. Obs: här avses utfodring av vintergrupp (slakt- och livren) i hägn och/eller på fribete i över två veckor, men ej under flytt och samling som pågår under en kortare period än två veckor. inneholder noen av disse alternativene

- Ja

2. Effekter av fôring

97) Opplever du en forandring i oppførselen til reinsdyr som fôres under en sammenhengende periode på minst to uker når de slippes igjen på fribeite? (for eksempel grad av tamhet, lettere eller vanskeligere å samle, obs: refererer ikke til atferdsendring under fôring).

	Ja	Nei	Vet ikke
Blant reinsdyr fôret i innhegning	○	○	○
Blant reinsdyr fôret på fribeite	○	○	○

Denne informasjonen vises kun i forhåndsvisningen

Følgende betingelser må være oppfylt for at spørsmålet skal vises for respondenten:

Dersom spørsmålet Har du utfodrat (nöd- eller stödutfodrat) dina renar i din vintergruppen vid något tillfälle under de senaste fem åren? Om samebyn inte är uppdelad i olika vintergrupper svarar du för hela samebyn. Obs: här avses utfodring av vintergrupp (slakt- och livren) i hägn och/eller på fribete i över två veckor, men ej under flytt och samling som pågår under en kortare period än två veckor. inneholder noen av disse alternativene

- Ja

98) Plass for kommentarer:

Denne informasjonen vises kun i forhåndsvisningen

Følgende betingelser må være oppfylt for at spørsmålet skal vises for respondenten:

Dersom spørsmålet Har du utfodrat (nöd- eller stödutfodrat) dina renar i din vintergruppen vid något tillfälle under de senaste fem åren? Om samebyn inte är uppdelad i olika vintergrupper svarar du för hela samebyn. Obs: här avses utfodring av vintergrupp (slakt- och livren) i hägn och/eller på fribete i över två veckor, men ej under flytt och samling som pågår under en kortare period än två veckor. inneholder noen av disse alternativene

- Ja

2. Effekter av fôring

99) Opplever du at kalver som fôres under vinteren har høyere slaktevekt påfølgende høst sammenlignet med kalver som ikke fôres?

○ Ja
○ Nei

- Vet ikke

Denne informasjonen vises kun i forhåndsvisningen

Følgende betingelser må være oppfylt for at spørsmålet skal vises for respondenten:

Dersom spørsmålet Har du utfodrat (nöd- eller stödutfodrat) dina renar i din vintergruppen vid något tillfälle under de senaste fem åren? Om samebyn inte är uppdelad i olika vintergrupper svarar du för hela samebyn. Obs: här avses utfodring av vintergrupp (slakt- och livren) i hägn och/eller på fribete i över två veckor, men ej under flytt och samling som pågår under en kortare period än två veckor. inneholder noen av disse alternativene

- Ja

100) Opplever du at kalvefrekvensen (andel simler med kalv) under kalvemerking og/eller om høsten blir positivt påvirket av fôringen av simler vinteren før?

- Ja
- Nei
- Vet ikke

Denne informasjonen vises kun i forhåndsvisningen

Følgende betingelser må være oppfylt for at spørsmålet skal vises for respondenten:

Dersom spørsmålet Har du utfodrat (nöd- eller stödutfodrat) dina renar i din vintergruppen vid något tillfälle under de senaste fem åren? Om samebyn inte är uppdelad i olika vintergrupper svarar du för hela samebyn. Obs: här avses utfodring av vintergrupp (slakt- och livren) i hägn och/eller på fribete i över två veckor, men ej under flytt och samling som pågår under en kortare period än två veckor. inneholder noen av disse alternativene

- Ja

101) Plass for kommentarer:

Denne informasjonen vises kun i forhåndsvisningen

Følgende betingelser må være oppfylt for at spørsmålet skal vises for respondenten:

Dersom spørsmålet Har du utfodrat (nöd- eller stödutfodrat) dina renar i din vintergruppen vid något tillfälle under de senaste fem åren? Om samebyn inte är uppdelad i olika vintergrupper svarar du för hela samebyn. Obs: här avses utfodring av

vintergrupp (slakt- och livren) i hägn och/eller på fribete i över två veckor, men ej under flytt och samling som pågår under en kortare period än två veckor. inneholder noen av disse alternativene

- Ja

2. Effekter av fôring

102) Opplever du at fôring påvirker kalvenes evne til å søke og finne beite etterfølgende vinter?

- ○ Ja
- ○ Nei
- ○ Vet ikke

Denne informasjonen vises kun i forhåndsvisningen

Følgende betingelser må være oppfylt for at spørsmålet skal vises for respondenten:

Dersom spørsmålet Har du utfodrat (nöd- eller stödutfodrat) dina renar i din vintergruppen vid något tillfälle under de senaste fem åren? Om samebyn inte är uppdelad i olika vintergrupper svarar du för hela samebyn. Obs: här avses utfodring av vintergrupp (slakt- och livren) i hägn och/eller på fribete i över två veckor, men ej under flytt och samling som pågår under en kortare period än två veckor. inneholder noen av disse alternativene

- Ja

103) Plass for kommentarer:

Denne informasjonen vises kun i forhåndsvisningen

Følgende betingelser må være oppfylt for at spørsmålet skal vises for respondenten:

Dersom spørsmålet Har du utfodrat (nöd- eller stödutfodrat) dina renar i din vintergruppen vid något tillfälle under de senaste fem åren? Om samebyn inte är uppdelad i olika vintergrupper svarar du för hela samebyn. Obs: här avses utfodring av vintergrupp (slakt- och livren) i hägn och/eller på fribete i över två veckor, men ej under flytt och samling som pågår under en kortare period än två veckor. inneholder noen av disse alternativene

- Ja

og

Dersom spørsmålet 2019/2020 inneholder noen av disse alternativene

- Fôret på fribeite
- Fôret i innhegningen

3. Spørsmål relatert til fôringsrutiner vinter- og/eller vårsesongen i din vintergruppe, 2019/2020.

Dette refererer til fôring (nød- eller tilleggsfôring) av reinsdyrene dine (slakterein og livrein) i vintergruppen i over to uker, i innhegning eller på fribeite, men ikke under flytting og samling som varer en kortere periode enn to uker. Om vintergruppen ikke er delt, svarer du for hele Siida enheten. Spørsmålene er ment for å kartlegge rutiner ved fôring i dagens situasjon.

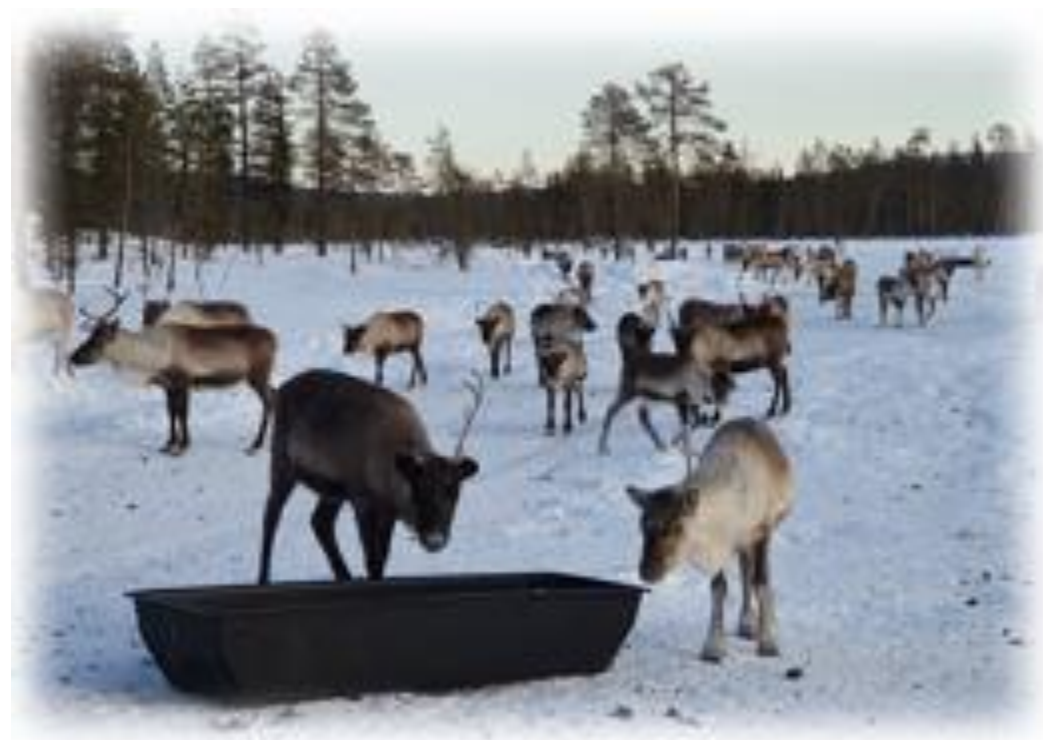

104) Følgende spørsmål gjelder sesongen 2019/2020. Omtrent hvor mange reinsdyr ble fôret totalt i din vintergruppe?

	I innhegning	På fribeite
Under 100	☐	☐
100-299	☐	☐
300-499	☐	☐
500-999	☐	☐
Over 1000	☐	☐

Denne informasjonen vises kun i forhåndsvisningen

Følgende betingelser må være oppfylt for at spørsmålet skal vises for respondenten:

Dersom spørsmålet Har du utfodrat (nöd- eller stödutfodrat) dina renar i din vintergruppen vid något tillfälle under de senaste fem åren? Om samebyn inte är uppdelad i olika vintergrupper svarar du för hela samebyn. Obs: här avses utfodring av vintergrupp (slakt- och livren) i hägn och/eller på fribete i över två veckor, men ej under flytt och samling som pågår under en kortare period än två veckor. inneholder noen av disse alternativene

- Ja

og

Dersom spørsmålet 2019/2020 inneholder noen av disse alternativene

- Fôret på fribeite
- Fôret i innhegningen

105) Hvilken/hvilke grupper av dyr ble hovedsakelig fôret av følgende tre kategorier, og hvor befant reinen seg da? Velg ett/flere alternativ.

	I innhegning	På fribeite
Hele vintergruppen	☐	☐
I hovedsak kalver (1 år eller yngre)	☐	☐
I hovedsak voksne dyr (over 1 år)	☐	☐

Denne informasjonen vises kun i forhåndsvisningen

Følgende betingelser må være oppfylt for at spørsmålet skal vises for respondenten:

Dersom spørsmålet Har du utfodrat (nöd- eller stödutfodrat) dina renar i din vintergruppen vid något tillfälle under de senaste fem åren? Om samebyn inte är uppdelad i olika vintergrupper svarar du för hela samebyn. Obs: här avses utfodring av vintergrupp (slakt- och livren) i hägn och/eller på fribete i över två veckor, men ej under flytt och samling som pågår under en kortare period än två veckor. inneholder noen av disse alternativene

- Ja

106) Plass for kommentarer:

Denne informasjonen vises kun i forhåndsvisningen

Følgende betingelser må være oppfylt for at spørsmålet skal vises for respondenten:

Dersom spørsmålet Har du utfodrat (nöd- eller stödutfodrat) dina renar i din vintergruppen vid något tillfälle under de senaste fem åren? Om samebyn inte är uppdelad i olika vintergrupper svarar du för hela samebyn. Obs: här avses utfodring av vintergrupp (slakt- och livren) i hägn och/eller på fribete i över två veckor, men ej under flytt och samling som pågår under en kortare period än två veckor. inneholder noen av disse alternativene

- Ja

og

Dersom spørsmålet 2019/2020 inneholder noen av disse alternativene

- Fôret på fribeite
- Fôret i innhegningen

3. Spørsmål relatert til fôringsrutiner vinter- og/eller vårsesongen i din vintergruppe, 2019/2020.

107) Omtrent hvor lenge fôret du reinsdyrene dine sammenhengende (oppgi fra den første reinen som ble fôret)? Henviser til nød- eller tilleggsfôring over en to ukers periode i løpet av 2019/2020.

	Mindre enn 1 måned	1-3 måneder	Over 3 måneder
I innhegning	○	○	○
På fribeite	○	○	○

Denne informasjonen vises kun i forhåndsvisningen

Følgende betingelser må være oppfylt for at spørsmålet skal vises for respondenten:

Dersom spørsmålet Har du utfodrat (nöd- eller stödutfodrat) dina renar i din vintergruppen vid något tillfälle under de senaste fem åren? Om samebyn inte är uppdelad i olika vintergrupper svarar du för hela samebyn. Obs: här avses utfodring av vintergrupp (slakt- och livren) i hägn och/eller på fribete i över två veckor, men ej under flytt och samling som pågår under en kortare period än två veckor. inneholder noen av disse alternativene

- Ja

og

Dersom spørsmålet 2019/2020 inneholder noen av disse alternativene

- Fôret på fribeite
- Fôret i innhegningen

108) Plass for kommentarer:

Denne informasjonen vises kun i forhåndsvisningen

Følgende betingelser må være oppfylt for at spørsmålet skal vises for respondenten:

Dersom spørsmålet Har du utfodrat (nöd- eller stödutfodrat) dina renar i din vintergruppen vid något tillfälle under de senaste fem åren? Om samebyn inte är uppdelad i olika vintergrupper svarar du för hela samebyn. Obs: här avses utfodring av vintergrupp (slakt- och livren) i hägn och/eller på fribete i över två veckor, men ej under flytt och samling som pågår under en kortare period än två veckor. inneholder noen av disse alternativene

- Ja

og

Dersom spørsmålet 2019/2020 inneholder noen av disse alternativene

- Fôret i innhegningen

3. Spørsmål relatert til fôringsrutiner vinter- og/eller vårsesongen i din vintergruppe, 2019/2020.

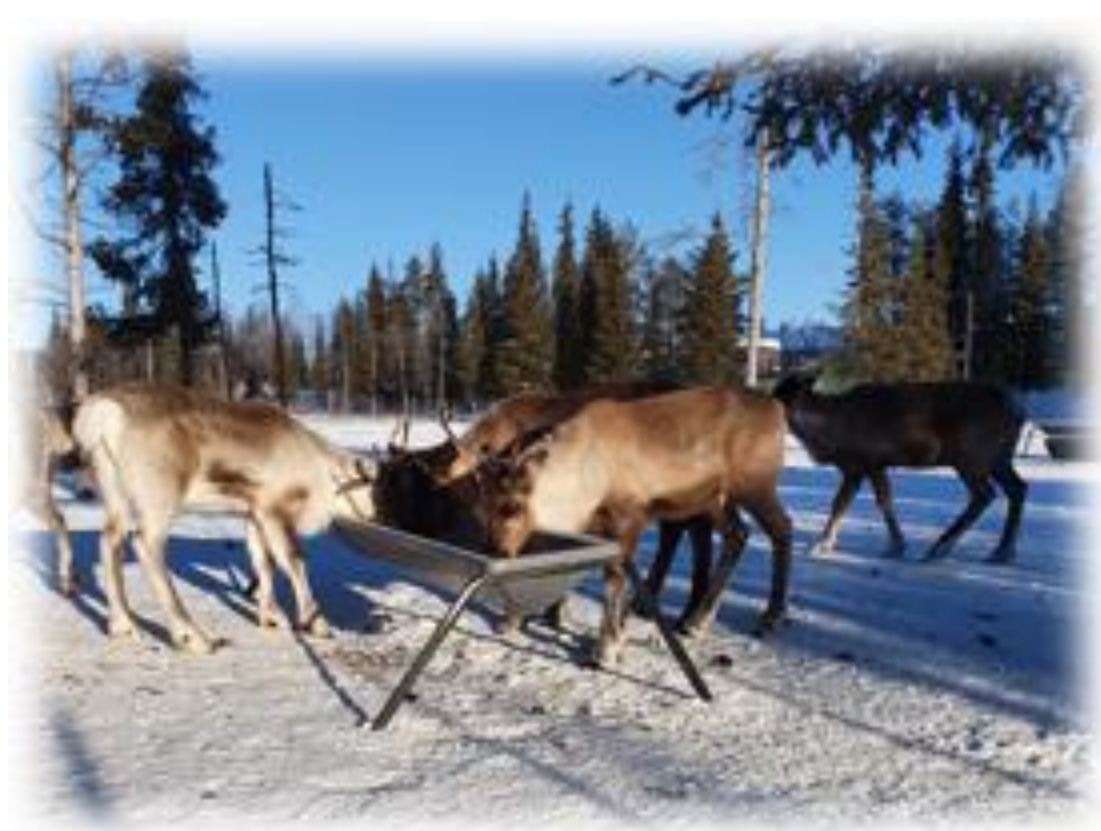

Spørsmålene er ment for å kartlegge rutiner ved fôring i dagens situasjon.

109) * For å kartlegge rutiner og forhold innen reindrift, følger spørsmål om forskjellige fôr. Hvilke av følgende fôr, fôret du reinsdyrene dine med i innhegning 2019/2020?

- Kun grovfôr (silofôr/surfôr/hösilage/høy)
- Kun pellets
- Kombination av grovfoder och pellets

Denne informasjonen vises kun i forhåndsvisningen

Følgende betingelser må være oppfylt for at spørsmålet skal vises for respondenten:

Dersom spørsmålet Har du utfodrat (nöd- eller stödutfodrat) dina renar i din vintergruppen vid något tillfälle under de senaste fem åren? Om samebyn inte är uppdelad i olika vintergrupper svarar du för hela samebyn. Obs: här avses utfodring av vintergrupp (slakt- och livren) i hägn och/eller på fribete i över två veckor, men ej under flytt och samling som pågår under en kortare period än två veckor. inneholder noen av disse alternativene

- Ja

og

Dersom spørsmålet 2019/2020 inneholder noen av disse alternativene

- Fôret på fribeite

110) * Hvilke av følgende fôr, fôret du reinsdyrene dine med på fribeite 2019/2020?

- Kun grovfôr (silofôr/surfôr/hösilage/høy)
- Kun pellets
- Kombination av grovfoder och pellets

Denne informasjonen vises kun i forhåndsvisningen

Følgende betingelser må være oppfylt for at spørsmålet skal vises for respondenten:

Dersom spørsmålet Har du utfodrat (nöd- eller stödutfodrat) dina renar i din vintergruppen vid något tillfälle under de senaste fem åren? Om samebyn inte är uppdelad i olika vintergrupper svarar du för hela samebyn. Obs: här avses utfodring av vintergrupp (slakt- och livren) i hägn och/eller på fribete i över två veckor, men ej under flytt och samling som pågår under en kortare period än två veckor. inneholder noen av disse alternativene

- Ja

og

Dersom spørsmålet 2019/2020 inneholder noen av disse alternativene

- Fôret på fribeite
- Fôret i innhegningen

111) Plass for kommentarer:

Denne informasjonen vises kun i forhåndsvisningen

Følgende betingelser må være oppfylt for at spørsmålet skal vises for respondenten:

Dersom spørsmålet Har du utfodrat (nöd- eller stödutfodrat) dina renar i din vintergruppen vid något tillfälle under de senaste fem åren? Om samebyn inte är uppdelad i olika vintergrupper svarar du för hela samebyn. Obs: här avses utfodring av vintergrupp (slakt- och livren) i hägn och/eller på fribete i över två veckor, men ej under flytt och samling som pågår under en kortare period än två veckor. inneholder noen av disse alternativene

- Ja

og

Dersom spørsmålet 2019/2020 inneholder noen av disse alternativene

- Fôret på fribeite
- Fôret i innhegningen

3.Spørsmål relatert til fôringsrutiner vinter- og/eller vårsesongen i din vintergruppe, 2019/2020.

112) Når fôret du med rein- og/eller hengelav utover det som fantes tilgjengelig i naturen 2019/2020? Oppgi ett / flere valg.

	I innhegning	På fribeite
Som tilskudd til fôring	☐	☐
Til syke/svake reinsdyr	☐	☐
Ved tilvenning av fôr	☐	☐
Har ikke forutsetning / tilgang til lav	☐	☐
Annet, oppgi i kommentarfeltet under	☐	☐

Denne informasjonen vises kun i forhåndsvisningen

Følgende betingelser må være oppfylt for at spørsmålet skal vises for respondenten:

Dersom spørsmålet Har du utfodrat (nöd- eller stödutfodrat) dina renar i din vintergruppen vid något tillfälle under de senaste fem åren? Om samebyn inte är uppdelad i olika vintergrupper svarar du för hela samebyn. Obs: här avses utfodring av vintergrupp (slakt- och livren) i hägn och/eller på fribete i över två veckor, men ej under flytt och samling som pågår under en kortare period än två veckor. inneholder noen av disse alternativene

- Ja

og

Dersom spørsmålet 2019/2020 inneholder noen av disse alternativene

- Fôret på fribeite
- Fôret i innhegningen

og

Dersom spørsmålet För att kartlägga rutiner och förutsättningar inom renskötseln följer frågor om olika fodermedel. Vilka av följande fodermedel utfodrade du dina renar med i hägn 2019/2020? inneholder noen av disse alternativene

- Kombination av grovfoder och pellets
- Kun grovfôr (silofôr/surfôr/hösilage/høy)

og

Dersom spørsmålet Vilka av följande fodermedel utfodrade du dina renar med på fribete 2019/2020? inneholder noen av disse alternativene

- Kombination av grovfoder och pellets
- Kun grovfôr (silofôr/surfôr/hösilage/høy)

113) Om mulig, estimer antall grovfôr- / silofôrballer og oppgi typen (f.eks. rund-, eller finkantball) som ble brukt under fôringsperioden 2019/2020?

I innhegning

På fribeite

Denne informasjonen vises kun i forhåndsvisningen

Følgende betingelser må være oppfylt for at spørsmålet skal vises for respondenten:

Dersom spørsmålet Har du utfodrat (nöd- eller stödutfodrat) dina renar i din vintergruppen vid något tillfälle under de senaste fem åren? Om samebyn inte är uppdelad i olika vintergrupper svarar du för hela samebyn. Obs: här avses utfodring av vintergrupp (slakt- och livren) i hägn och/eller på fribete i över två veckor, men ej under flytt och samling som pågår under en kortare period än två veckor. inneholder noen av disse alternativene

- Ja

og

Dersom spørsmålet 2019/2020 inneholder noen av disse alternativene

- Fôret på fribeite
- Fôret i innhegningen

114) Plass for kommentarer:

Denne informasjonen vises kun i forhåndsvisningen

Følgende betingelser må være oppfylt for at spørsmålet skal vises for respondenten:

Dersom spørsmålet Har du utfodrat (nöd- eller stödutfodrat) dina renar i din vintergruppen vid något tillfälle under de senaste fem åren? Om samebyn inte är uppdelad i olika vintergrupper svarar du för hela samebyn. Obs: här avses utfodring av vintergrupp (slakt- och livren) i hägn och/eller på fribete i över två veckor, men ej under flytt och samling som pågår under en kortare period än två veckor. inneholder noen av disse alternativene

- Ja

og

Dersom spørsmålet 2019/2020 inneholder noen av disse alternativene

- Fôret på fribeite
- Fôret i innhegningen

og

Dersom spørsmålet För att kartlägga rutiner och förutsättningar inom renskötseln följer frågor om olika fodermedel. Vilka av följande fodermedel utfodrade du dina renar med i hägn 2019/2020? inneholder noen av disse alternativene

- Kombination av grovfoder och pellets
- Kun pellets

og

Dersom spørsmålet Vilka av följande fodermedel utfodrade du dina renar med på fribete 2019/2020? inneholder noen av disse alternativene

- Kombination av grovfoder och pellets
- Kun pellets

3. Spørsmål relatert til fôringsrutiner vinter- og/eller vårsesongen i din vintergruppe, 2019/2020.

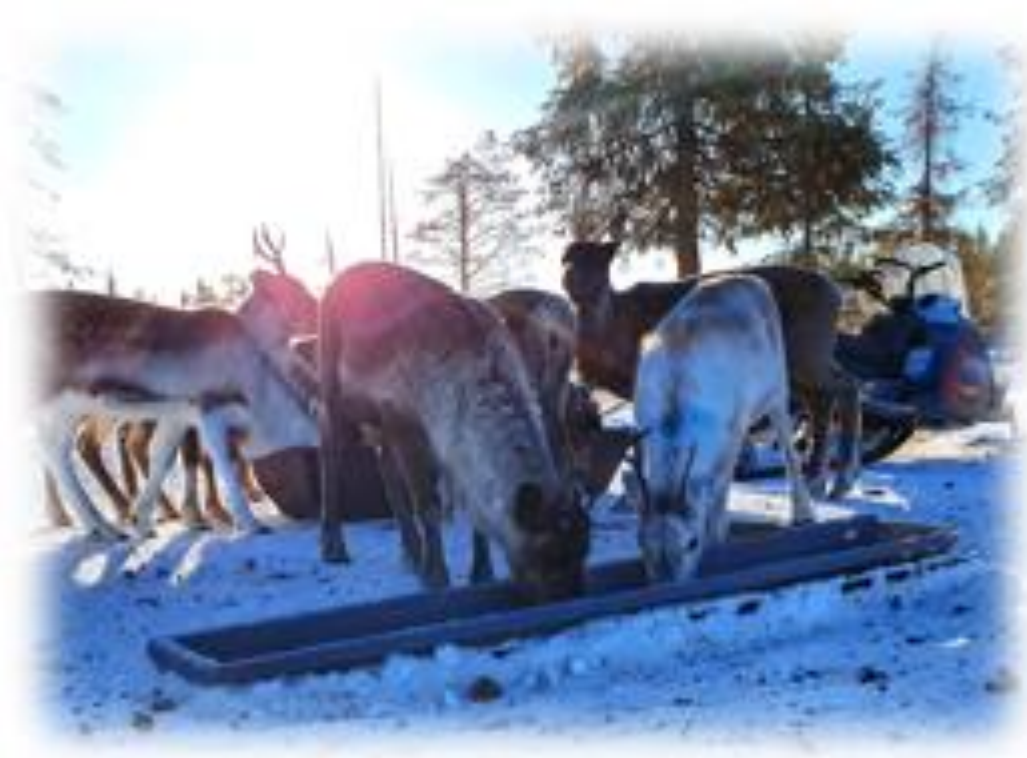

115) Oppgi antall fôringsøkter per dag med pellets (2019/2020).

	1	2 eller flere
I innhegning	◯	◯
På fribeite	◯	◯

Denne informasjonen vises kun i forhåndsvisningen

Følgende betingelser må være oppfylt for at spørsmålet skal vises for respondenten:

Dersom spørsmålet Har du utfodrat (nöd- eller stödutfodrat) dina renar i din vintergruppen vid något tillfälle under de senaste fem åren? Om samebyn inte är uppdelad i olika vintergrupper svarar du för hela samebyn. Obs: här avses utfodring av vintergrupp (slakt- och livren) i hägn och/eller på fribete i över två veckor, men ej under flytt och samling som pågår under en kortare period än två veckor. inneholder noen av disse alternativene

- Ja

og

Dersom spørsmålet 2019/2020 inneholder noen av disse alternativene

- Fôret på fribeite
- Fôret i innhegningen

og

Dersom spørsmålet För att kartlägga rutiner och förutsättningar inom renskötseln följer frågor om olika fodermedel. Vilka av följande fodermedel utfodrade du dina renar med i hägn 2019/2020? inneholder noen av disse alternativene

- Kombination av grovfoder och pellets
- Kun pellets

og

Dersom spørsmålet Vilka av följande fodermedel utfodrade du dina renar med på fribete 2019/2020? inneholder noen av disse alternativene

- Kombination av grovfoder och pellets
- Kun pellets

116) Om mulig, estimer antall kilo pellets per dyr og dag i gjennomsnitt for sesongen 2019/2020 og oppgi om det gjelder for nød- og / eller tilleggsfôring..

	Nødfôring	Tilleggsfôring
I innhegning		
På fribeite		

Denne informasjonen vises kun i forhåndsvisningen

Følgende betingelser må være oppfylt for at spørsmålet skal vises for respondenten:

Dersom spørsmålet Har du utfodrat (nöd- eller stödutfodrat) dina renar i din vintergruppen vid något tillfälle under de senaste fem åren? Om samebyn inte är uppdelad i olika vintergrupper svarar du för hela samebyn. Obs: här avses utfodring av vintergrupp (slakt- och livren) i hägn och/eller på fribete i över två veckor, men ej under flytt och samling som pågår under en kortare period än två veckor. inneholder noen av disse alternativene

- Ja

og

Dersom spørsmålet 2019/2020 inneholder noen av disse alternativene

- Fôret på fribeite
- Fôret i innhegningen

og

Dersom spørsmålet För att kartlägga rutiner och förutsättningar inom renskötseln följer frågor om olika fodermedel. Vilka av följande fodermedel utfodrade du dina renar med i hägn 2019/2020? inneholder noen av disse alternativene

- Kombination av grovfoder och pellets
- Kun pellets

og

Dersom spørsmålet Vilka av följande fodermedel utfodrade du dina renar med på fribete 2019/2020? inneholder noen av disse alternativene

- Kombination av grovfoder och pellets
- Kun pellets

117) Plass for kommentarer:

Denne informasjonen vises kun i forhåndsvisningen

Følgende betingelser må være oppfylt for at spørsmålet skal vises for respondenten:

Dersom spørsmålet Har du utfodrat (nöd- eller stödutfodrat) dina renar i din vintergruppen vid något tillfälle under de senaste fem åren? Om samebyn inte är uppdelad i olika vintergrupper svarar du för hela samebyn. Obs: här avses utfodring av vintergrupp (slakt- och livren) i hägn och/eller på fribete i över två veckor, men ej under flytt och samling som pågår under en kortare period än två veckor. inneholder noen av disse alternativene

- Ja

og

Dersom spørsmålet 2019/2020 inneholder noen av disse alternativene

- Fôret på fribeite
- Fôret i innhegningen

og

Dersom spørsmålet För att kartlägga rutiner och förutsättningar inom renskötseln följer frågor om olika fodermedel. Vilka av följande fodermedel utfodrade du dina renar med i hägn 2019/2020? inneholder noen av disse alternativene

- Kombination av grovfoder och pellets
- Kun pellets

og

Dersom spørsmålet Vilka av följande fodermedel utfodrade du dina renar med på fribete 2019/2020? inneholder noen av disse alternativene

- Kombination av grovfoder och pellets
- Kun pellets

3. Spørsmål relatert til fôringsrutiner vinter- og/eller vårsesongen i din vintergruppe, 2019/2020.

118) Om mulig, oppgi fôrleverandør av pellets under 2019/2020.

I innhegning

På fribeite

Denne informasjonen vises kun i forhåndsvisningen

Følgende betingelser må være oppfylt for at spørsmålet skal vises for respondenten:

Dersom spørsmålet Har du utfodrat (nöd- eller stödutfodrat) dina renar i din vintergruppen vid något tillfälle under de senaste fem åren? Om samebyn inte är uppdelad i olika vintergrupper svarar du för hela samebyn. Obs: här avses utfodring av vintergrupp (slakt- och livren) i hägn och/eller på fribete i över två veckor, men ej under flytt och samling som pågår under en kortare period än två veckor. inneholder noen av disse alternativene

- Ja

og

Dersom spørsmålet 2019/2020 inneholder noen av disse alternativene

- Fôret på fribeite
- Fôret i innhegningen

og

Dersom spørsmålet För att kartlägga rutiner och förutsättningar inom renskötseln följer frågor om olika fodermedel. Vilka av följande fodermedel utfodrade du dina renar med i hägn 2019/2020? inneholder noen av disse alternativene

- Kombination av grovfoder och pellets
- Kun pellets

og

Dersom spørsmålet Vilka av följande fodermedel utfodrade du dina renar med på fribete 2019/2020? inneholder noen av disse alternativene

- Kombination av grovfoder och pellets
- Kun pellets

119) Hvordan utførtes fôring med pellets vanligvis under forrige sesong (2019/2020)? Kryss av i ett/flere alternativ.

	I innhegning	På fribeite
Direkte på bakken	☐	☐
Fôringshekk på ben	☐	☐
Fôringshekk uten ben	☐	☐
Annet, oppgi i kommentarfeltet under.	☐	☐

Denne informasjonen vises kun i forhåndsvisningen

Følgende betingelser må være oppfylt for at spørsmålet skal vises for respondenten:

Dersom spørsmålet Har du utfodrat (nöd- eller stödutfodrat) dina renar i din vintergruppen vid något tillfälle under de senaste fem åren? Om samebyn inte är uppdelad i olika vintergrupper svarar du för hela samebyn. Obs: här avses utfodring av vintergrupp (slakt- och livren) i hägn och/eller på fribete i över två veckor, men ej under flytt och samling som pågår under en kortare period än två veckor. inneholder noen av disse alternativene

- Ja

og

Dersom spørsmålet 2019/2020 inneholder noen av disse alternativene

- Fôret på fribeite
- Fôret i innhegningen

og

Dersom spørsmålet För att kartlägga rutiner och förutsättningar inom renskötseln följer frågor om olika fodermedel. Vilka av följande fodermedel utfodrade du dina renar med i hägn 2019/2020? inneholder noen av disse alternativene

- Kombination av grovfoder och pellets
- Kun pellets

og

Dersom spørsmålet Vilka av följande fodermedel utfodrade du dina renar med på fribete 2019/2020? inneholder noen av disse alternativene

- Kombination av grovfoder och pellets
- Kun pellets

120) Plass for kommentarer:

Denne informasjonen vises kun i forhåndsvisningen

Følgende betingelser må være oppfylt for at spørsmålet skal vises for respondenten:

Dersom spørsmålet Har du utfodrat (nöd- eller stödutfodrat) dina renar i din vintergruppen vid något tillfälle under de senaste fem åren? Om samebyn inte är uppdelad i olika vintergrupper svarar du för hela samebyn. Obs: här avses utfodring av vintergrupp (slakt- och livren) i hägn och/eller på fribete i över två veckor, men ej under flytt och samling som pågår under en kortare period än två veckor. inneholder noen av disse alternativene

- Ja

4. Spørsmål relatert til andre fôrtyper og fôringsrutiner under de siste fem årene.

Dette refererer fortsatt til fôring (nød- eller tilleggsfôring) av reinsdyrene dine (slakterein og livrein) i vintergruppen i over to uker, i innhegning eller på fribeite, men ikke under flytting og samling som varer en kortere periode enn to uker. Om vintergruppen ikke er delt, svarer du for hele Siida enheten.

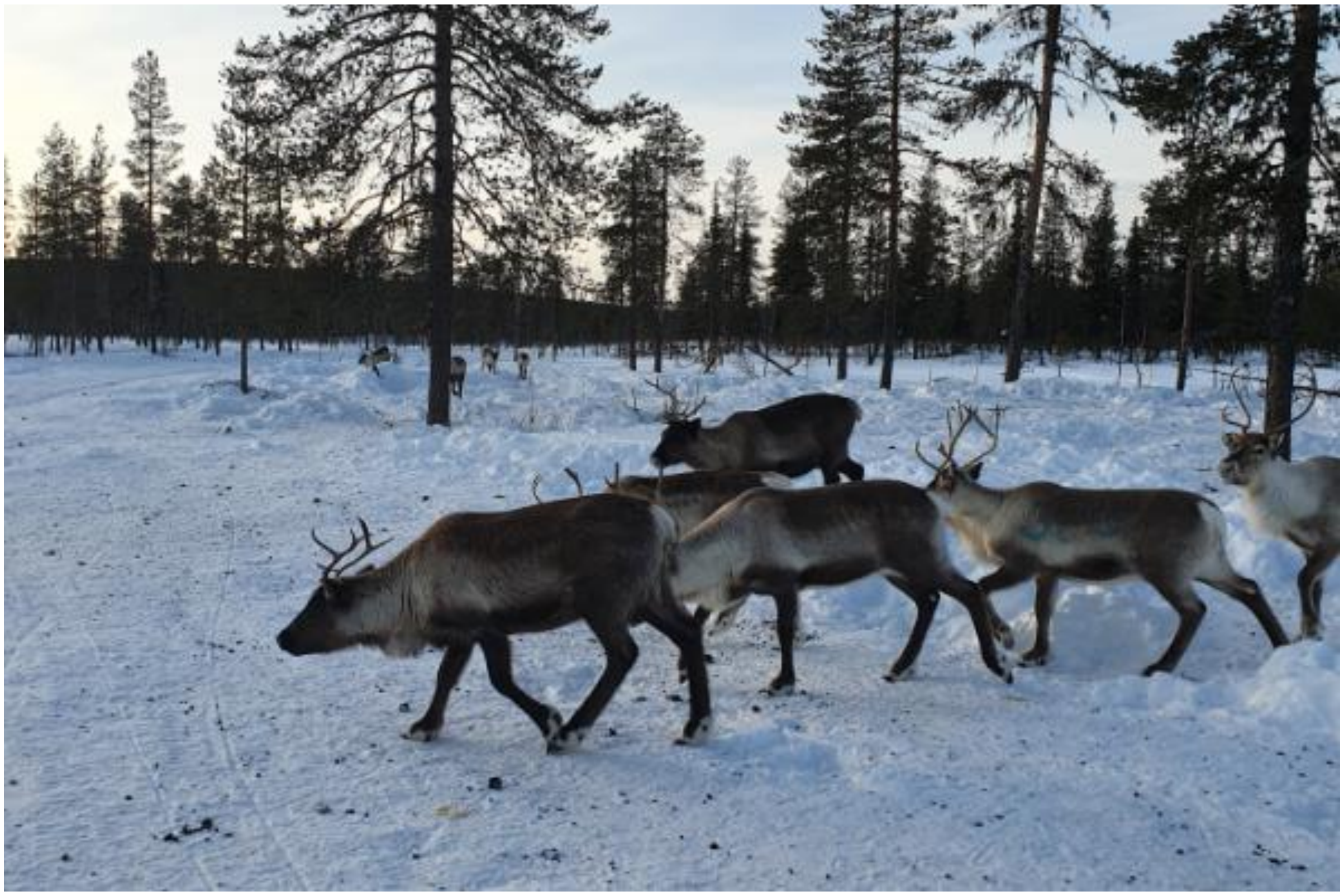

Spørsmålene er ment for å kartlegge rutiner ved fôring i dagens situasjon.

121) Hvor vanlig er det at reinen gis tilgang til mineraltilskudd (f.eks. mineralsaltstein, mineralbalje, fôrgjær, injeksjon) under fôring de siste fem årene? Velg ett / flere alternativ.

	I innhegning	På fribeite
Alltid under fôring (angi som eneste svarsalternativ)	☐	☐
Periodevis under fôring	☐	☐
Enkelte sesonger	☐	☐
Aldri (angi som eneste svarsalternativ)	☐	☐
Under spesielle omstendigheter (f.eks. til reinsdyr i dårlig tilstand og i syke innhegning)	☐	☐
Annet, oppgi i kommentarfeltet under.	☐	☐

Denne informasjonen vises kun i forhåndsvisningen

Følgende betingelser må være oppfylt for at spørsmålet skal vises for respondenten:

Dersom spørsmålet Har du utfodrat (nöd- eller stödutfodrat) dina renar i din vintergruppen vid något tillfälle under de senaste fem åren? Om samebyn inte är uppdelad i olika vintergrupper svarar du för hela samebyn. Obs: här avses utfodring av vintergrupp (slakt- och livren) i hägn och/eller på fribete i över två veckor, men ej under flytt och samling som pågår under en kortare period än två veckor. inneholder noen av disse alternativene

- Ja

122) Plass for kommentarer:

Denne informasjonen vises kun i forhåndsvisningen

Følgende betingelser må være oppfylt for at spørsmålet skal vises for respondenten:

Dersom spørsmålet Har du utfodrat (nöd- eller stödutfodrat) dina renar i din vintergruppen vid något tillfälle under de senaste fem åren? Om samebyn inte är uppdelad i olika vintergrupper svarar du för hela samebyn. Obs: här avses utfodring av vintergrupp (slakt- och livren) i hägn och/eller på fribete i över två veckor, men ej under

flytt och samling som pågår under en kortare period än två veckor. inneholder noen av disse alternativene

- Ja

123) Hvis du benyttet mineraler, oppgi merket og typen du sist benyttet.

I innhegning

På fribeite

Denne informasjonen vises kun i forhåndsvisningen

Følgende betingelser må være oppfylt for at spørsmålet skal vises for respondenten:

Dersom spørsmålet Har du utfodrat (nöd- eller stödutfodrat) dina renar i din vintergruppen vid något tillfälle under de senaste fem åren? Om samebyn inte är uppdelad i olika vintergrupper svarar du för hela samebyn. Obs: här avses utfodring av vintergrupp (slakt- och livren) i hägn och/eller på fribete i över två veckor, men ej under flytt och samling som pågår under en kortare period än två veckor. inneholder noen av disse alternativene

- Ja

4. Spørsmål relatert til andre fôrtyper og fôringsrutiner under de siste fem årene.

124) Hvor vanlig er det at reinsdyrene får tilgang til saltstein ved fôring (tenk på de siste fem årene)? Velg ett/flere alternativ.

	I innhegning	På fribeite
Alltid under fôring (angi som eneste svarsalternativ)	☐	☐
Periodevis under fôring	☐	☐
Enkelte sesonger	☐	☐
Aldri (angi som eneste svarsalternativ)	☐	☐
Under spesielle omstendigheter (f.eks. til reinsdyr i dårlig tilstand og i syke innhegning)	☐	☐
Annet, oppgi i kommentarfeltet under.	☐	☐

Denne informasjonen vises kun i forhåndsvisningen

Følgende betingelser må være oppfylt for at spørsmålet skal vises for respondenten:

Dersom spørsmålet Har du utfodrat (nöd- eller stödutfodrat) dina renar i din vintergruppen vid något tillfälle under de senaste fem åren? Om samebyn inte är uppdelad i olika vintergrupper svarar du för hela samebyn. Obs: här avses utfodring av vintergrupp (slakt- och livren) i hägn och/eller på fribete i över två veckor, men ej under flytt och samling som pågår under en kortare period än två veckor. inneholder noen av disse alternativene

- Ja

125) Oppgi andre fôrmidler som anvendtes de siste fem årene (for eksempel fôrgjær) .

I innhegning

På fribeite

Denne informasjonen vises kun i forhåndsvisningen

Følgende betingelser må være oppfylt for at spørsmålet skal vises for respondenten:

Dersom spørsmålet Har du utfodrat (nöd- eller stödutfodrat) dina renar i din vintergruppen vid något tillfälle under de senaste fem åren? Om samebyn inte är uppdelad i olika vintergrupper svarar du för hela samebyn. Obs: här avses utfodring av vintergrupp (slakt- och livren) i hägn och/eller på fribete i över två veckor, men ej under flytt och samling som pågår under en kortare period än två veckor. inneholder noen av disse alternativene

- Ja

126) Plass for kommentarer:

Denne informasjonen vises kun i forhåndsvisningen

Følgende betingelser må være oppfylt for at spørsmålet skal vises for respondenten:

Dersom spørsmålet Har du utfodrat (nöd- eller stödutfodrat) dina renar i din vintergruppen vid något tillfälle under de senaste fem åren? Om samebyn inte är uppdelad i olika vintergrupper svarar du för hela samebyn. Obs: här avses utfodring av vintergrupp (slakt- och livren) i hägn och/eller på fribete i över två veckor, men ej under flytt och samling som pågår under en kortare period än två veckor. inneholder noen av disse alternativene

- Ja

eller

Dersom spørsmålet 2015/2016 inneholder noen av disse alternativene

- Fôret i innhegningen

eller

Dersom spørsmålet 2016/2017 inneholder noen av disse alternativene

- Fôret i innhegningen

eller

Dersom spørsmålet 2017/2018 inneholder noen av disse alternativene

- Fôret i innhegningen

eller

Dersom spørsmålet 2018/2019 inneholder noen av disse alternativene

- Fôret i innhegningen

eller

Dersom spørsmålet 2019/2020 inneholder noen av disse alternativene

- Fôret i innhegningen

eller

Dersom spørsmålet 2020/2021 inneholder noen av disse alternativene

- Fôret i innhegningen

4. Spørsmål relatert til andre fôrtyper og fôringsrutiner under de siste fem årene.

127) Oppgi vanntilgang ved fôring i innhegning de siste fem årene. Velg ett / flere alternativ.

☐Fri tilgang til snø
☐Snø i balje
☐Vann i balje
☐Bekk
☐Kald kilde
☐Annet, oppgi i kommentarfeltet under.

Denne informasjonen vises kun i forhåndsvisningen

Følgende betingelser må være oppfylt for at spørsmålet skal vises for respondenten:

Dersom spørsmålet Har du utfodrat (nöd- eller stödutfodrat) dina renar i din vintergruppen vid något tillfälle under de senaste fem åren? Om samebyn inte är uppdelad i olika vintergrupper svarar du för hela samebyn. Obs: här avses utfodring av vintergrupp (slakt- och livren) i hägn och/eller på fribete i över två veckor, men ej under flytt och samling som pågår under en kortare period än två veckor. inneholder noen av disse alternativene

- Ja

eller

Dersom spørsmålet 2015/2016 inneholder noen av disse alternativene

- Fôret i innhegningen

eller

Dersom spørsmålet 2016/2017 inneholder noen av disse alternativene

- Fôret i innhegningen

eller

Dersom spørsmålet 2017/2018 inneholder noen av disse alternativene

- Fôret i innhegningen

eller

Dersom spørsmålet 2018/2019 inneholder noen av disse alternativene

- Fôret i innhegningen

eller

Dersom spørsmålet 2019/2020 inneholder noen av disse alternativene

- Fôret i innhegningen

128) Plass for kommentarer:

Denne informasjonen vises kun i forhåndsvisningen

Følgende betingelser må være oppfylt for at spørsmålet skal vises for respondenten:

Dersom spørsmålet Har du utfodrat (nöd- eller stödutfodrat) dina renar i din vintergruppen vid något tillfälle under de senaste fem åren? Om samebyn inte är uppdelad i olika vintergrupper svarar du för hela samebyn. Obs: här avses utfodring av vintergrupp (slakt- och livren) i hägn och/eller på fribete i över två veckor, men ej under

flytt och samling som pågår under en kortare period än två veckor. inneholder noen av disse alternativene

- Ja

4. Spørsmål relatert til andre fôrtyper og fôringsrutiner under de siste fem årene.

129) Finnes det rutiner for tilvenning av fôret?

- ○ Ja
- ○ Nei

Denne informasjonen vises kun i forhåndsvisningen

Følgende betingelser må være oppfylt for at spørsmålet skal vises for respondenten:

Dersom spørsmålet Har du utfodrat (nöd- eller stödutfodrat) dina renar i din vintergruppen vid något tillfälle under de senaste fem åren? Om samebyn inte är uppdelad i olika vintergrupper svarar du för hela samebyn. Obs: här avses utfodring av vintergrupp (slakt- och livren) i hägn och/eller på fribete i över två veckor, men ej under flytt och samling som pågår under en kortare period än två veckor. inneholder noen av disse alternativene

- Ja

130) Plass for kommentarer:

Denne informasjonen vises kun i forhåndsvisningen

Følgende betingelser må være oppfylt for at spørsmålet skal vises for respondenten:

Dersom spørsmålet Har du utfodrat (nöd- eller stödutfodrat) dina renar i din vintergruppen vid något tillfälle under de senaste fem åren? Om samebyn inte är uppdelad i olika vintergrupper svarar du för hela samebyn. Obs: här avses utfodring av vintergrupp (slakt- och livren) i hägn och/eller på fribete i över två veckor, men ej under flytt och samling som pågår under en kortare period än två veckor. inneholder noen av disse alternativene

- Ja

4. Spørsmål relatert til andre fôrtyper og fôringsrutiner under de siste fem årene.

131) * Tar du vanligvis bort gammelt fôr før du fôrer med nytt?

- Ja
- Nei

Denne informasjonen vises kun i forhåndsvisningen

Følgende betingelser må være oppfylt for at spørsmålet skal vises for respondenten:

Dersom spørsmålet Har du utfodrat (nöd- eller stödutfodrat) dina renar i din vintergruppen vid något tillfälle under de senaste fem åren? Om samebyn inte är uppdelad i olika vintergrupper svarar du för hela samebyn. Obs: här avses utfodring av vintergrupp (slakt- och livren) i hägn och/eller på fribete i över två veckor, men ej under flytt och samling som pågår under en kortare period än två veckor. inneholder noen av disse alternativene

- Ja

132) Plass for kommentarer:

Denne informasjonen vises kun i forhåndsvisningen

Følgende betingelser må være oppfylt for at spørsmålet skal vises for respondenten:

Dersom spørsmålet Har du utfodrat (nöd- eller stödutfodrat) dina renar i din vintergruppen vid något tillfälle under de senaste fem åren? Om samebyn inte är uppdelad i olika vintergrupper svarar du för hela samebyn. Obs: här avses utfodring av vintergrupp (slakt- och livren) i hägn och/eller på fribete i över två veckor, men ej under flytt och samling som pågår under en kortare period än två veckor. inneholder noen av disse alternativene

- Ja

og

Dersom spørsmålet Tar du vanligtvis bort överblivet foder innan du utfodrar med nytt? inneholder noen av disse alternativene

- Ja

4. Spørsmål relatert til andre fôrtyper og fôringsrutiner under de siste fem årene.

133) Hvor tømmer du vanligvis gammelt fôr?

- I innhegningen
- Utenfor innhegningen
- Annet, oppgi i kommentarfeltet under

Denne informasjonen vises kun i forhåndsvisningen

Følgende betingelser må være oppfylt for at spørsmålet skal vises for respondenten:

Dersom spørsmålet Har du utfodrat (nöd- eller stödutfodrat) dina renar i din vintergruppen vid något tillfälle under de senaste fem åren? Om samebyn inte är uppdelad i olika vintergrupper svarar du för hela samebyn. Obs: här avses utfodring av vintergrupp (slakt- och livren) i hägn och/eller på fribete i över två veckor, men ej under flytt och samling som pågår under en kortare period än två veckor. inneholder noen av disse alternativene

- Ja

og

Dersom spørsmålet Tar du vanligtvis bort överblivet foder innan du utfodrar med nytt? inneholder noen av disse alternativene

- Ja

134) Plass for kommentarer:

Denne informasjonen vises kun i forhåndsvisningen

Følgende betingelser må være oppfylt for at spørsmålet skal vises for respondenten:

Dersom spørsmålet Har du utfodrat (nöd- eller stödutfodrat) dina renar i din vintergruppen vid något tillfälle under de senaste fem åren? Om samebyn inte är uppdelad i olika vintergrupper svarar du för hela samebyn. Obs: här avses utfodring av vintergrupp (slakt- och livren) i hägn och/eller på fribete i över två veckor, men ej under flytt och samling som pågår under en kortare period än två veckor. inneholder noen av disse alternativene

- Ja

Her er de siste spørsmålene relatert til fôring:

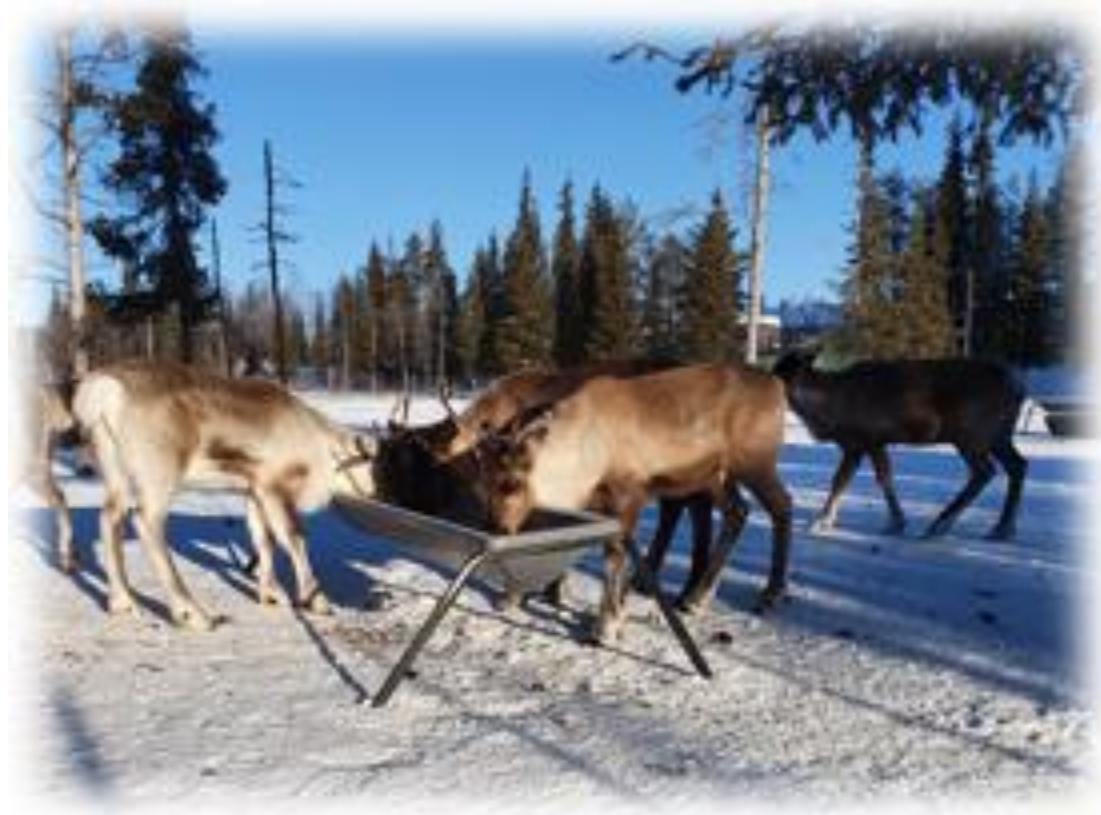

135) Påvirker tilgangen av ulike fôrmidler (silofôr/surfôr /pellets/lav) ditt valg av fôr? Forklar gjerne i kommentarfeltet under.

- Ja, oppgi hvordan i kommentarfeltet under
- Nei

Denne informasjonen vises kun i forhåndsvisningen

Følgende betingelser må være oppfylt for at spørsmålet skal vises for respondenten:

Dersom spørsmålet Har du utfodrat (nöd- eller stödutfodrat) dina renar i din vintergruppen vid något tillfälle under de senaste fem åren? Om samebyn inte är uppdelad i olika vintergrupper svarar du för hela samebyn. Obs: här avses utfodring av vintergrupp (slakt- och livren) i hägn och/eller på fribete i över två veckor, men ej under flytt och samling som pågår under en kortare period än två veckor. inneholder noen av disse alternativene

- Ja

136) Etterspør du analyse av grovfôret med hensyn til næringsinnhold og/eller hygiene?

- Ja, næringsinnhold
- Ja, hygiene
- Ja, både næringsinnhold og hygiene
- Nei

Denne informasjonen vises kun i forhåndsvisningen

Følgende betingelser må være oppfylt for at spørsmålet skal vises for respondenten:

Dersom spørsmålet Har du utfodrat (nöd- eller stödutfodrat) dina renar i din vintergruppen vid något tillfälle under de senaste fem åren? Om samebyn inte är uppdelad i olika vintergrupper svarar du för hela samebyn. Obs: här avses utfodring av

vintergrupp (slakt- och livren) i hägn och/eller på fribete i över två veckor, men ej under flytt och samling som pågår under en kortare period än två veckor. inneholder noen av disse alternativene

- Ja

137) Plass for kommentarer:

Denne informasjonen vises kun i forhåndsvisningen

Følgende betingelser må være oppfylt for at spørsmålet skal vises for respondenten:

Dersom spørsmålet Har du utfodrat (nöd- eller stödutfodrat) dina renar i din vintergruppen vid något tillfälle under de senaste fem åren? Om samebyn inte är uppdelad i olika vintergrupper svarar du för hela samebyn. Obs: här avses utfodring av vintergrupp (slakt- och livren) i hägn och/eller på fribete i över två veckor, men ej under flytt och samling som pågår under en kortare period än två veckor. inneholder noen av disse alternativene

- Ja

138) Legg gjerne igjen andre kommentarer relatert til fôring og / eller andre refleksjoner på undersøkelsen her:

Denne informasjonen vises kun i forhåndsvisningen

Actions vil skje for følgende alternativer:

Ja, jeg vil vite mer : Omdiriger til en ekstern URL (https://response.questback.com/statensveterinrmedicinskaansta/erfarenheteravsjukdomigaochmunhosren)

Bli med og bidra til mer kunnskap om reinens helse, fôring og ta del i det siste innen forskning på reinsdyr!

Vi søker deltakere som ønsker å delta i et planlagt dybdeintervju angående reinens helse og effekter av fôring. Intervjuet vil i hovedsak bli gjennomført av Karin Wallin Philippot i Sverige og av Alfa Josteinsdottir i Norge, der vi sammen blir enige om en tid og sted for gjennomføringen.

139) * Vil du være med og bidra til mer kunnskap og ta del i det siste innen forskning om smittsomme sykdommer hos reinsdyr?

- Nei takk
- Ja, jeg vil vite mer

© Copyright www.questback.com. All Rights Reserved.
Trial Essentials for free - Click here to create your survey today.
