# Supplementary material for: Reindeer health and supplementary feeding practices – a survey among herders in Norway and Sweden
Source: Acta Vet Scand. 2026 Sep 22;68:36. doi: 10.1186/s13028-026-00868-3 (PMC13595754; doi:10.1186/s13028-026-00868-3)
Supplement: Supplementary file 3 [file 13028_2026_868_MOESM3_ESM.pdf]

Additional file 3 The questionnaire survey (in Northern Sámi) regarding health and supplementary feeding of semi-domesticated reindeer, distributed among reindeer herders in Norway and Sweden in 2021.

Bures boahtin!

Dát guorahallan guoská dutnje gii barggat bohccuiguin Norggas, beroškeahttá biepmat go bohccuid vai it. Guorahallama ádjána vástidit sullii 30 minuhtta ja dat leat juhkkojuvvon máŋgga oassái:

1. Gažaldagat golmma njoammudávdda birra ja muđui bohccuid dearvvašvuođa birra.
2. Gažaldagat biebmama birra (váikkuhusat, rutiinnat ja eará).

FUOMÁŠ: Du vástádusat eai vurkejuvvo jus don heaittát vástideame gaskan, dehe gaskkalduvat, ja de ii leat maiddái šat vejolašvuohta máhccat ruovttoluotta guorahallamii, danne go buot vástádusat jávket ja don fertet fas áibbas ođđasit vástidišgoahtit.

Du identitehta čihkkojuvvo.

Jus eará ii leat namuhuvvon, de gusket dát gažaldagat dálvejagi dillái 2019 - 2020.

Gažaldagat gusket dan olbmui gii vástida ja searvá dán guorahallamii.

1) * Guđe riikkas lea du váldoboazodoallu?

- ○ Norggas
- ○ Ruoŧas

Denne informasjonen vises kun i forhåndsvisningen

Følgende betingelser må være oppfylt for at spørsmålet skal vises for respondenten:

If the question I vilket land bedriver du huvudsakligen renskötsel? contains any of these alternatives

- Ruoŧas

2) * Guđe guovllus lea dus váldosiiddastallanguovlu?

- ○ Dalarnas/Jämtlánddas
- ○ Västerbottenis
- ○ Norrbottenis

Denne informasjonen vises kun i forhåndsvisningen

Følgende betingelser må være oppfylt for at spørsmålet skal vises for respondenten:

If the question I vilket land bedriver du huvudsakligen renskötsel? contains any of these alternatives

- Ruoŧas

3) * Guđe siiddas/čearus barggat bohccuiguin?

- Duottarčearru
- Vuovdečearru
- Konsešuvdnačearru

Denne informasjonen vises kun i forhåndsvisningen

Følgende betingelser må være oppfylt for at spørsmålet skal vises for respondenten:

If the question I vilket land bedriver du huvudsakligen renskötsel? contains any of these alternatives

- Norggas

4) * Guđe guovllus lea dus váldosiiddastallanguovlu?

- Nuorta-Finnmárkkus
- Oarje-Finnmárkkus
- Romssas
- Nordlánndas
- Davvi-Trøndelágas
- Lulli-Trøndelágas
- Møre ja Romsdálas
- Hedemárkkus

Denne informasjonen vises kun i forhåndsvisningen

Følgende betingelser må være oppfylt for at spørsmålet skal vises for respondenten:

If the question I vilket land bedriver du huvudsakligen renskötsel? contains any of these alternatives

- Norggas

5) * Man guhká leat bargan bohccuiguin (siiddastallan)?

- Vuollel 5 jagi
- 5–9 jagi
- 10–29 jagi
- Badjel 30 jagi

6) Sohkabealli?

- ○ Nisu
- ○ Dievdu
- ○ In hálit vástidit

7) Ahki?

- ○ Nuorat go 20 jagi
- ○ 20-39 jagi
- ○ 40-59 jagi
- ○ badjel 60 jagi

8) * Sullii man stuora eallu lea dus (bohccot dálvesiiddas) maŋŋil jahkásaš njuovvama?

- ○ Vuollel 50
- ○ 50-99
- ○ 100-249
- ○ 250-499
- ○ 500-999
- ○ 1000-1999
- ○ 2000-2999
- ○ Badjel 3000

Dás leat gažaldagat golmma vuolšindávdda/-vigi birra mat soitet vuolgit biebmamis.

1. NJOAMMU ČALBMEVUOLŠI/-VIHKI / ČALMMIID RIEVDAN:

Njoammu čalbmevigi dehe čalmmiid rievdama sáhttá fuomášit go čalmmit golgagohtet ja njuoskistahttá čalmmevuole guolggaid. Vihki sáhttá vuolgit sihke virusis ja bakteriijain ja lea danne njoammuvihki ja sáhttá dagahit sierranas čalbmerievdamiid, nu go ovdamearkka dihte oainnát

Govain 1. A-C:

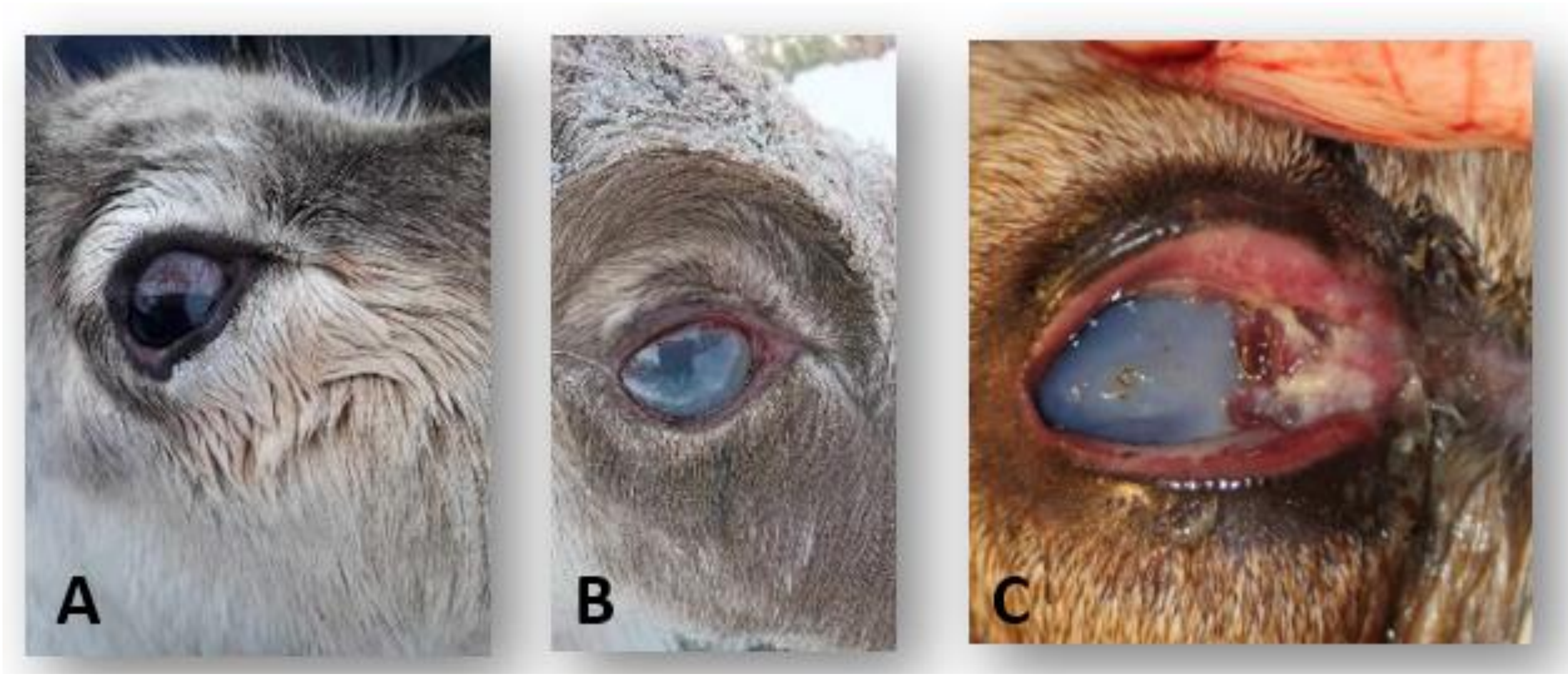

A: Čalmmiid golgan njuoskkistahttá čalmmevuole guolggaid – álgodássi.

B: Alitránes(dieigan) čoarvecuozza ja ruvssodan vuohččecuozza, guhkálmastan vihkemearkkat .

C: Rukses vuohččecuozza, čalbmi bohtanan ja čalmmit golget, guhkálmastan vihkemearkkat.

9) * Leat go don dan maŋemus 10 jagis oaidnán iežat bohccuin dakkár čalbmerievdamiid mat leat govain 1. A-C?

- Jua
- In
- In dieđe / Earálágán čalbmerievdama, čilges dás vulobeale makkár

10) Čilge dákko makkár čalbmerievdamiid don leat oaidnán:

Denne informasjonen vises kun i forhåndsvisningen

Følgende betingelser må være oppfylt for at spørsmålet skal vises for respondenten:

If the question Har du sett liknande ögonförändringar som Bild 1. A-C visar på dina renar under de senaste 10 åren? contains any of these alternatives

- In dieđe / Earálágán čalbmerievdama, čilges dás vulobeale makkár
- Jua

NJOAMMU ČALBMEVUOLŠI/-VIHKI / ČALMMIID RIEVDAN

Govain 1. A-C:

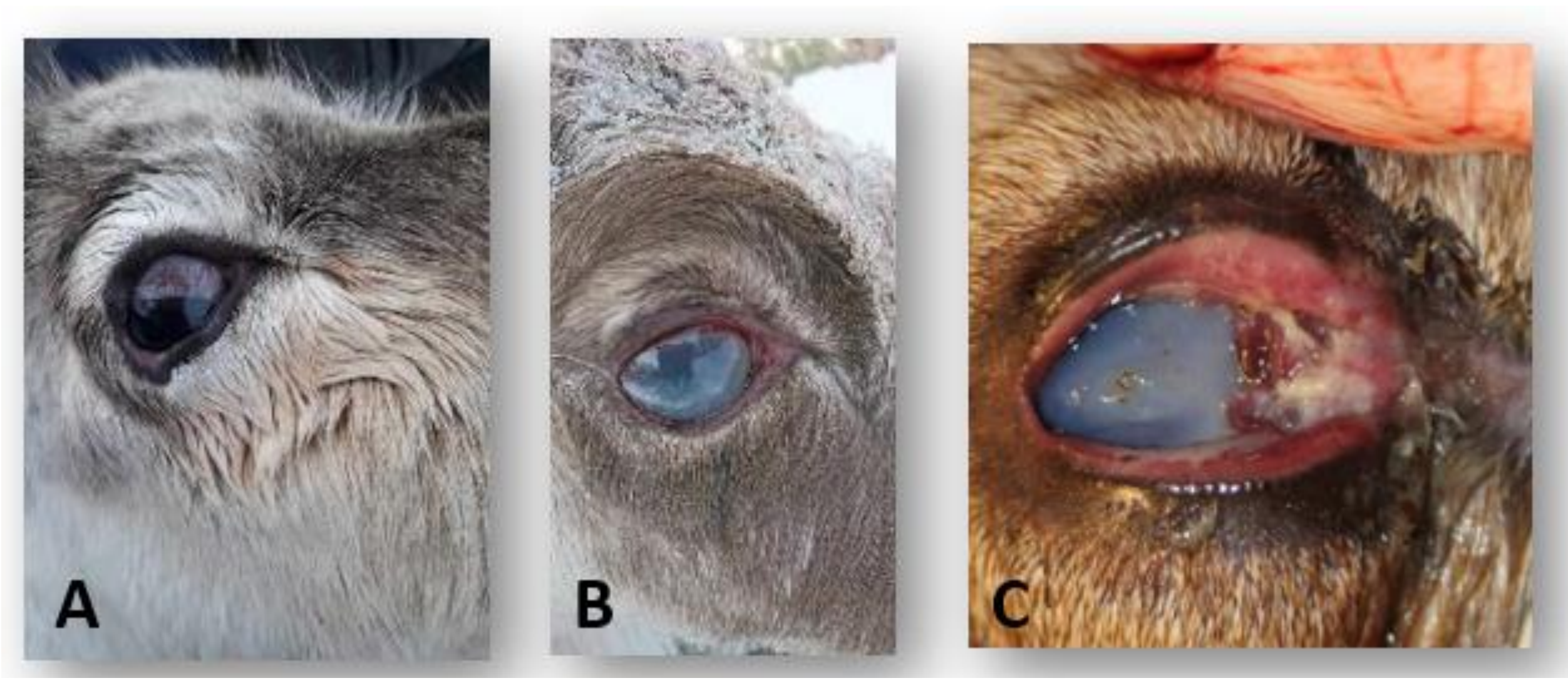

11) Goas oidnet maŋemus dakkár sullasaš čalbmerievdamiid go dat mat leat govain 1. A-C? Jus leat muitalan oaidnán earálágán čalbmerievdama/vigi mearkkaid, de vástidat čuovvovaš gažaldaga čalbmevuolšši/-rievdama dan ektui.

- Dán maŋemus jagi
- In dán maŋemus jagi gal, muhto dan maŋemus viđa jagis
- Eanet go vihtta jagi áigi
- In dieđe

Denne informasjonen vises kun i forhåndsvisningen

Følgende betingelser må være oppfylt for at spørsmålet skal vises for respondenten:

If the question Har du sett liknande ögonförändringar som Bild 1. A-C visar på dina renar under de senaste 10 åren? contains any of these alternatives

- In dieđe / Earálágán čalbmerievdama, čilges dás vulobeale makkár
- Jua

NJOAMMU ČALBMEVUOLŠI/-VIHKI / ČALMMIID RIEVDAN

Govain 1. A-C:

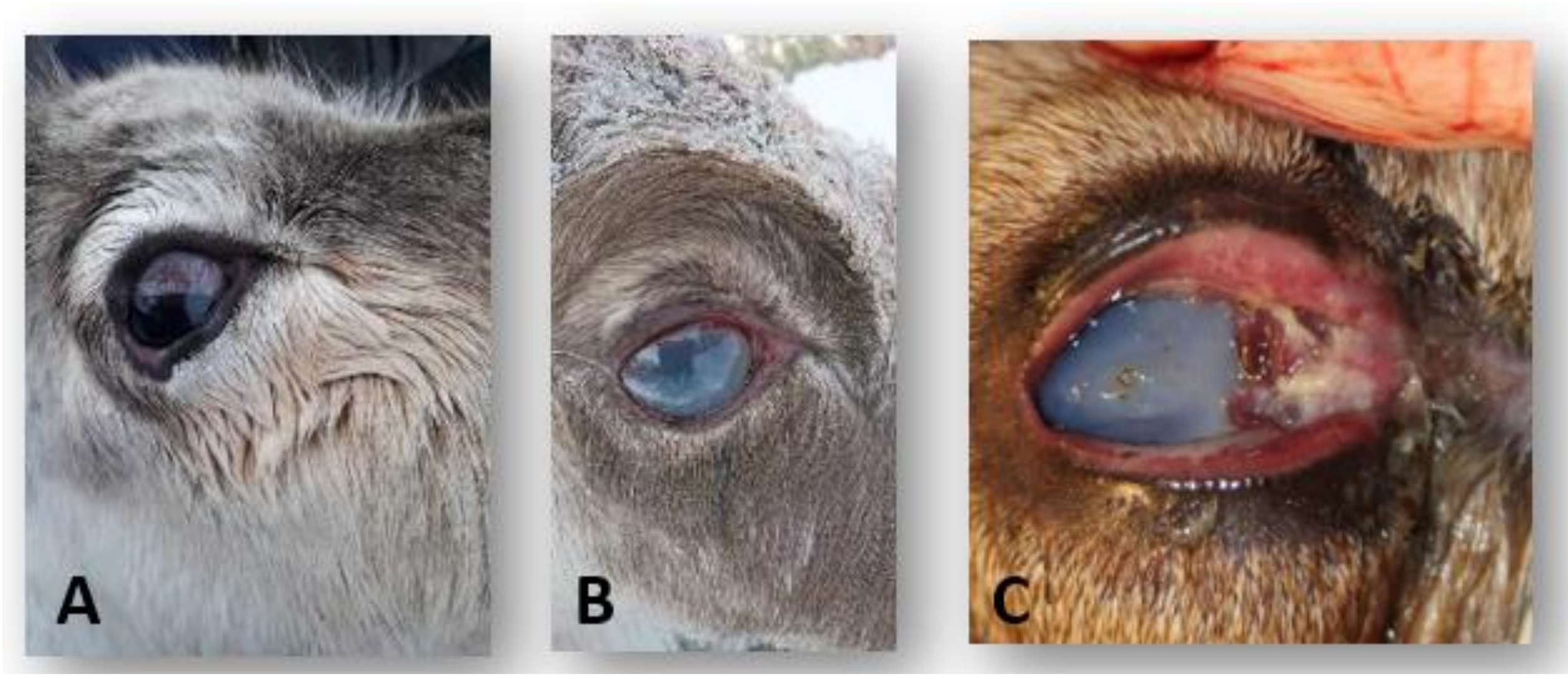

12) Guđemuš dávdamearkka/vigi, mat leat govain 1. A-C, leat dávjjimusat oaidnán dain bohccuin main lea/leamašan čalbmevihki?

	In goassege	Muhtomin	Dávjá
A	◯	◯	◯
B	◯	◯	◯
C	◯	◯	◯
Eará vihkemearkkat, makkárat:	◯	◯	◯

Denne informasjonen vises kun i forhåndsvisningen

Følgende betingelser må være oppfylt for at spørsmålet skal vises for respondenten:

If the question Har du sett liknande ögonförändringar som Bild 1. A-C visar på dina renar under de senaste 10 åren? contains any of these alternatives

- In dieđe / Earálágán čalbmerievdama, čilges dás vulobeale makkár
- Jua

13) Eará mearkkašumit:

Denne informasjonen vises kun i forhåndsvisningen

Følgende betingelser må være oppfylt for at spørsmålet skal vises for respondenten:

If the question Har du sett liknande ögonförändringar som Bild 1. A-C visar på dina renar under de senaste 10 åren? contains any of these alternatives

- In dieđe / Earálágán čalbmerievdama, čilges dás vulobeale makkár
- Jua

NJOAMMU ČALBMEVUOLŠI/-VIHKI / ČALMMIID RIEVDAN

14) Guđe muttos jagis leat oaidnán eanemusat bohccuid main leat čalbmevihki? Muital goas misiin, nuorra bohccuin ja rávis bohccuin. Lea vejolaš válljet sierranas áiggiid jagis.

	In leat oaidnán/ vuohttán dávdda	Giđđat	Geasset	Čakčat	Dálvet	Birra jagi
Miesit (vuollel jagi)	☐	☐	☐	☐	☐	☐
Nuorra bohccot (1-3 jagi)	☐	☐	☐	☐	☐	☐
Rávis bohccot (boarrásit go 3 jagi)	☐	☐	☐	☐	☐	☐

Denne informasjonen vises kun i forhåndsvisningen

Følgende betingelser må være oppfylt for at spørsmålet skal vises for respondenten:

If the question Har du sett liknande ögonförändringar som Bild 1. A-C visar på dina renar under de senaste 10 åren? contains any of these alternatives

- In dieđe / Earálágán čalbmerievdama, čilges dás vulobeale makkár
- Jua

15) Eará mearkkašumit:

Denne informasjonen vises kun i forhåndsvisningen

Følgende betingelser må være oppfylt for at spørsmålet skal vises for respondenten:

If the question Har du sett liknande ögonförändringar som Bild 1. A-C visar på dina renar under de senaste 10 åren? contains any of these alternatives

- In dieđe / Earálágán čalbmerievdama, čilges dás vulobeale makkár
- Jua

NJOAMMU ČALBMEVUOLŠI/-VIHKI / ČALMMIID RIEVDAN

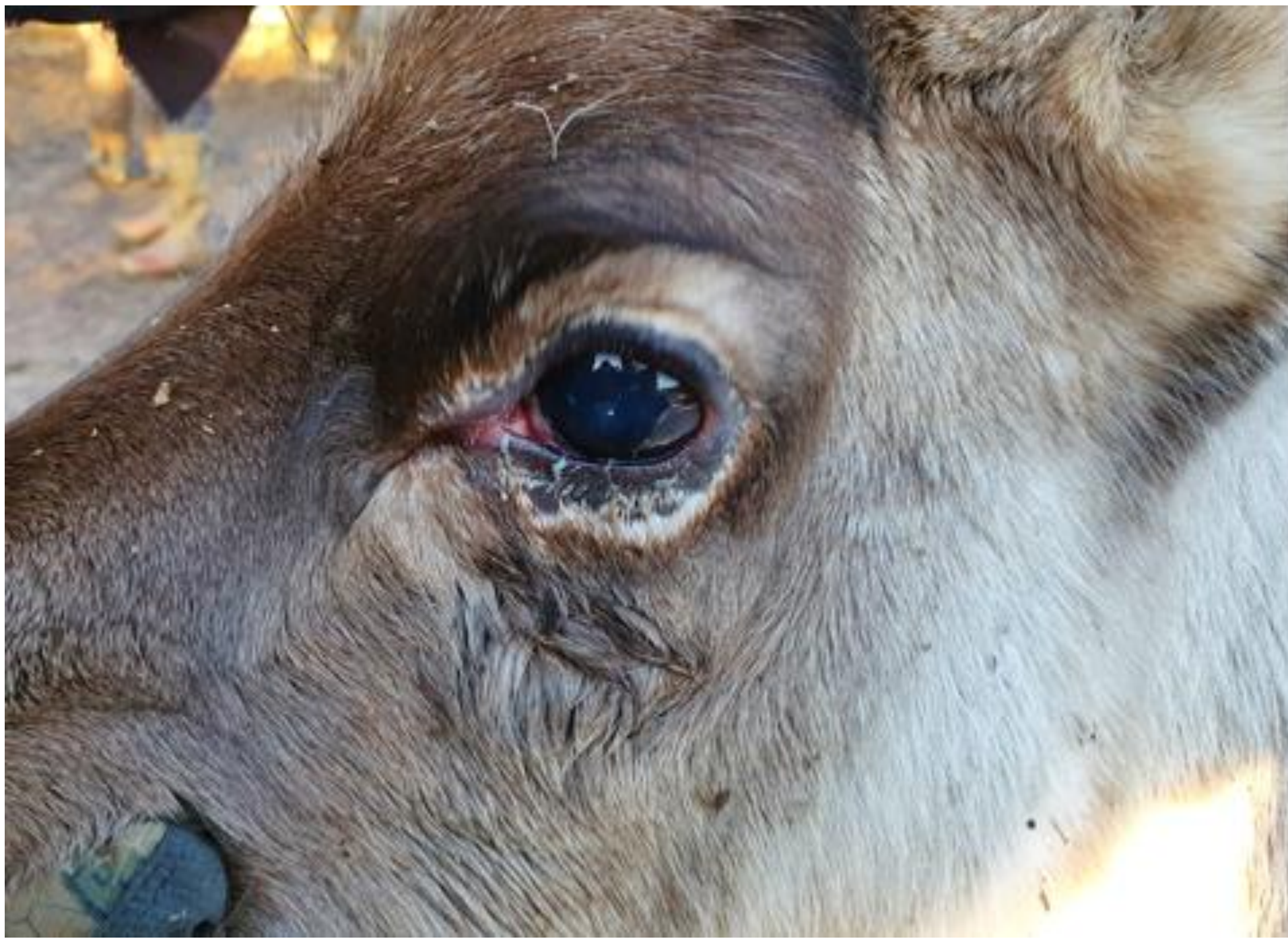

Boazu mas čalmmit golget.

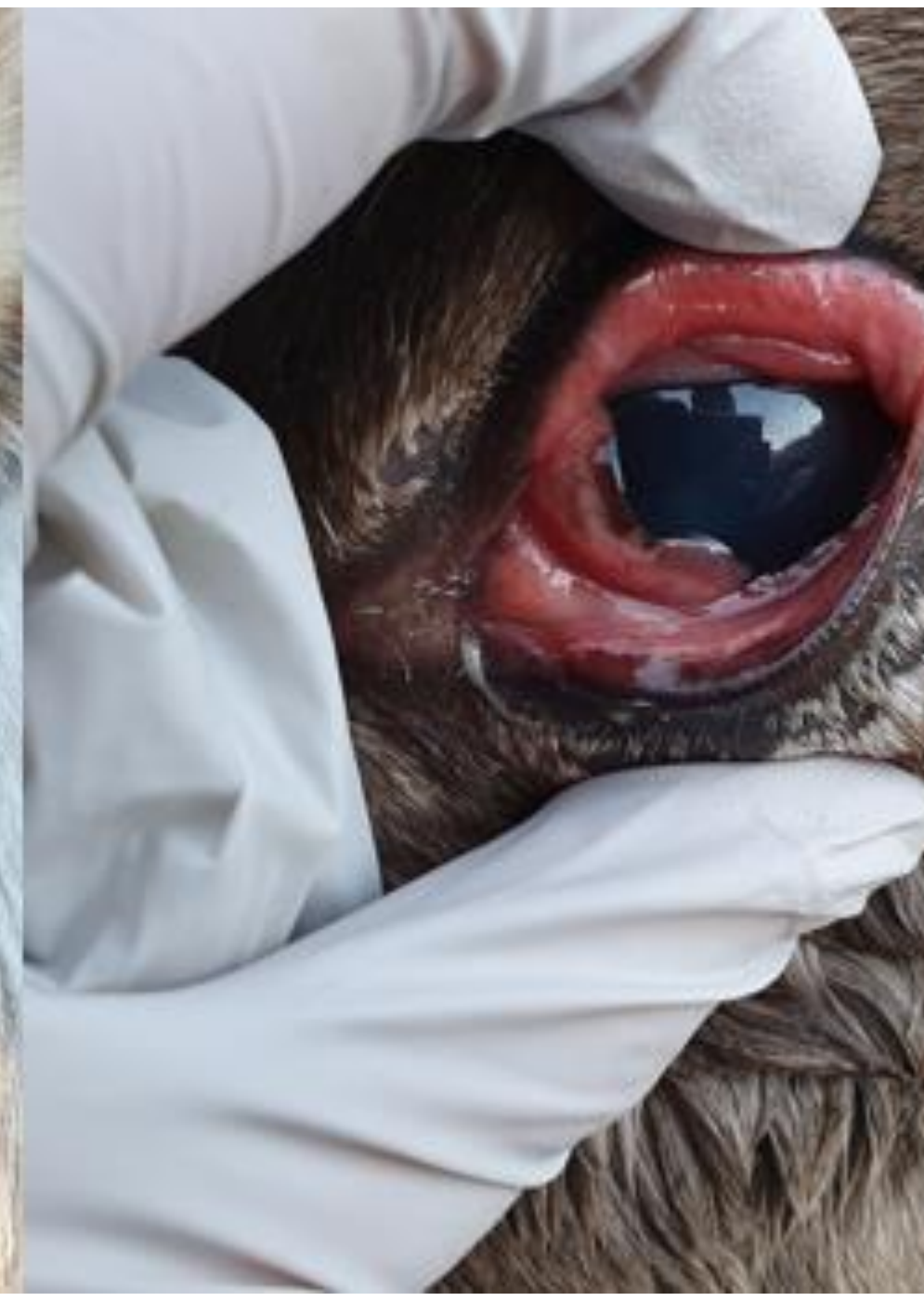

Čalbmi lea bohtas ja go iská dan lagabuidda de oaidná ahte lea maid ruvssodan.

16) Muital, juohke jagi áigodaga dáfus dás vulobealde, gos lei eallu/bohccot dalle go njoammu čalbmevihki /čalmmiid rievdan fuomášuvvui. Dehe jus bohccot eai dohppehallan dakkár vihkái. Vigi ihtin mearkkaša dan go vigivuolis bohccuid lohku lassána eambbo muhtun áiggi siste, ovdamearkka dihte dalle go dihto áiggi jagis eambbo bohccot go dábálaččat dohppehallet vihkái, dehe muhtun oanehit áiggis dohppehallet vihkái, omd. muhtun vahkuid siste.

	In leat oaidnán / vuohttán vigi/rievdama	Dohppehalle gárddis	Dohppehalle dalle go bibmojuvvo lávda ealus / luovos dievás	Dohppehalle lávda/ čoahkke ealu/ luovos dievá guohtuma oktavuođas
2015/2016	☐	☐	☐	☐
2016/2017	☐	☐	☐	☐
2017/2018	☐	☐	☐	☐
2018/2019	☐	☐	☐	☐

	In leat oaidnán / vuohttán vigi/rievdama	Dohppehalle gárddis	Dohppehalle dalle go bibmojuvvo lávda ealus / luovos dievás	Dohppehalle lávda/ čoahkke ealu/ luovos dievá guohtuma oktavuođas
2019/2020	☐	☐	☐	☐
2020/2021	☐	☐	☐	☐

Denne informasjonen vises kun i forhåndsvisningen

Følgende betingelser må være oppfylt for at spørsmålet skal vises for respondenten:

If the question Har du sett liknande ögonförändringar som Bild 1. A-C visar på dina renar under de senaste 10 åren? contains any of these alternatives

- In dieđe / Earálágán čalbmerievdama, čilges dás vulobeale makkár
- Jua

17) Eará mearkkašumit:

Denne informasjonen vises kun i forhåndsvisningen

Følgende betingelser må være oppfylt for at spørsmålet skal vises for respondenten:

If the question Har du sett liknande ögonförändringar som Bild 1. A-C visar på dina renar under de senaste 10 åren? contains any of these alternatives

- In dieđe / Earálágán čalbmerievdama, čilges dás vulobeale makkár
- Jua

NJOAMMU ČALBMEVUOLŠI/-VIHKI / ČALMMIID RIEVDAN

18) Sullii man galle bohccos ealus /siiddas lei vihki dan maŋemus háve dalle go bohccot dohppehalle njoammu čalbmevihkái/rivde čalmmit? Muital vuos álggos galle bohcco dohppehalle vihkái, jus leat vásihan dan.

Galle miesi (vuollel jahkásaččat)

Galle nuorra bohcco (1-3 jagi)

Galle rávis bohcco (boarrásit go 3 jagi)

Oktiibuot galle bohcco ledje olles ealus/siiddas dalle go dávda fuomášuvvui (sihke dearvvaš ja skihpa/dávdavuolis)

Denne informasjonen vises kun i forhåndsvisningen

Følgende betingelser må være oppfylt for at spørsmålet skal vises for respondenten:

If the question Har du sett liknande ögonförändringar som Bild 1. A-C visar på dina renar under de senaste 10 åren? contains any of these alternatives

- In dieđe / Earálágán čalbmerievdama, čilges dás vulobeale makkár
- Jua

19) Eará mearkkašumit:

Denne informasjonen vises kun i forhåndsvisningen

Følgende betingelser må være oppfylt for at spørsmålet skal vises for respondenten:

If the question Har du sett liknande ögonförändringar som Bild 1. A-C visar på dina renar under de senaste 10 åren? contains any of these alternatives

- In dieđe / Earálágán čalbmerievdama, čilges dás vulobeale makkár
- Jua

NJOAMMU ČALBMEVUOLŠI/-VIHKI / ČALMMIID RIEVDAN

20) * Leat go mearkkašan lea go bohccuid lohku, mat leat ožžon njoammu čalbmevigi/rievdan čalmmit, rievdan dan maŋemus 5 jagis?

- ○ Jua, dat leat lassánan
- ○ Jua, dat lea unnon
- ○ In, lohku ii leat lassánan iige unnon
- ○ In dieđe

Denne informasjonen vises kun i forhåndsvisningen

Følgende betingelser må være oppfylt for at spørsmålet skal vises for respondenten:

If the question Har du sett liknande ögonförändringar som Bild 1. A-C visar på dina renar under de senaste 10 åren? contains any of these alternatives

- In dieđe / Earálágán čalbmerievdama, čilges dás vulobeale makkár
- Jua

og

If the question Upplever du att antalet renar som drabbats av smittsam ögoninflammation/ögonförändring har förändrats över de senaste fem åren? contains any of these alternatives

- Jua, dat leat lassánan

NJOAMMU ČALBMEVUOLŠI/-VIHKI / ČALMMIID RIEVDAN

21) Manne jáhkát dat lohku lea lassánan?

Denne informasjonen vises kun i forhåndsvisningen

Følgende betingelser må være oppfylt for at spørsmålet skal vises for respondenten:

If the question Har du sett liknande ögonförändringar som Bild 1. A-C visar på dina renar under de senaste 10 åren? contains any of these alternatives

- In dieđe / Earálágán čalbmerievdama, čilges dás vulobeale makkár
- Jua

og

If the question Upplever du att antalet renar som drabbats av smittsam ögoninflammation/ögonförändring har förändrats över de senaste fem åren? contains any of these alternatives

- Jua, dat lea unnon

NJOAMMU ČALBMEVUOLŠI/-VIHKI / ČALMMIID RIEVDAN

22) Manne jáhkát dat lohku lea unnon?

Denne informasjonen vises kun i forhåndsvisningen

Følgende betingelser må være oppfylt for at spørsmålet skal vises for respondenten:

If the question Har du sett liknande ögonförändringar som Bild 1. A-C visar på dina renar under de senaste 10 åren? contains any of these alternatives

- In dieđe / Earálágán čalbmerievdama, čilges dás vulobeale makkár
- Jua

NJOAMMU ČALBMEVUOLŠI/-VIHKI / ČALMMIID RIEVDAN

23) * Álggahit go makkárge doaimmaid/dálkkodeami (omd. válddát bohccuid sierra, njuovat dehe gieđahalat eará ládje) dalle go fuomášat bohccuid main lea njoammu čalbmevihki/čalmmit rievdan?

- ○ Jua
- ○ In
- ○ In dieđe

Denne informasjonen vises kun i forhåndsvisningen

Følgende betingelser må være oppfylt for at spørsmålet skal vises for respondenten:

If the question Har du sett liknande ögonförändringar som Bild 1. A-C visar på dina renar under de senaste 10 åren? contains any of these alternatives

- In dieđe / Earálágán čalbmerievdama, čilges dás vulobeale makkár
- Jua

24) Maid barget, čađahit dalle maŋemus go fuomášit bohccuin njoammu čalbmevihki/čalmmiid rievdan? Merke buot doaibmabijuid/dálkkodeami mii álggahuvvui

- ☐ In bargan/čađahan maidege
- ☐ Skihpa/dávdavuolis bohccot sirrejuvvo sierra gárdái
- ☐ Válden oktavuođa šibitdoaktáriin
- ☐ Vihki dálkkoduvvui penicilliinnain mii náluin cirgguhuvvui deahkkái
- ☐ Vihki dálkkoduvvui penicilliinnain mii goaikkuhuvvo njuolga čalbmái
- ☐ Njuvvojuvvo
- ☐ Bohccot ráddjojuvvo (goddojuvvo) eret/bálkestuvvo
- ☐ Bohccot dálkkoduvvo eará dálkasiiguin, čális makkáriiguin dás vulobealde
- ☐ Eará, čilge dás vuolábealde maid ja mot

Denne informasjonen vises kun i forhåndsvisningen

Følgende betingelser må være oppfylt for at spørsmålet skal vises for respondenten:

If the question Har du sett liknande ögonförändringar som Bild 1. A-C visar på dina renar under de senaste 10 åren? contains any of these alternatives

- In dieđe / Earálágán čalbmerievdama, čilges dás vulobeale makkár
- Jua

25) Eará mearkkašumit:

Denne informasjonen vises kun i forhåndsvisningen

Følgende betingelser må være oppfylt for at spørsmålet skal vises for respondenten:

If the question Har du sett liknande ögonförändringar som Bild 1. A-C visar på dina renar under de senaste 10 åren? contains any of these alternatives

- In dieđe / Earálágán čalbmerievdama, čilges dás vulobeale makkár
- Jua

NJOAMMU ČALBMEVUOLŠI/-VIHKI / ČALMMIID RIEVDAN

26) Lea go njoammu čalbmevihki/čalmmiid rievdan váikkuhan dunnje ruđalaččat?

- ○ Jua
- ○ In
- ○ In dieđe

Denne informasjonen vises kun i forhåndsvisningen

Følgende betingelser må være oppfylt for at spørsmålet skal vises for respondenten:

If the question Har du sett liknande ögonförändringar som Bild 1. A-C visar på dina renar under de senaste 10 åren? contains any of these alternatives

- In dieđe / Earálágán čalbmerievdama, čilges dás vulobeale makkár
- Jua

27) Eará mearkkašumit:

Denne informasjonen vises kun i forhåndsvisningen

Følgende betingelser må være oppfylt for at spørsmålet skal vises for respondenten:

If the question Har du sett liknande ögonförändringar som Bild 1. A-C visar på dina renar under de senaste 10 åren? contains any of these alternatives

- In dieđe / Earálágán čalbmerievdama, čilges dás vulobeale makkár
- Jua

NJOAMMU ČALBMEVUOLŠI/-VIHKI / ČALMMIID RIEVDAN

Govain 1. A-C:

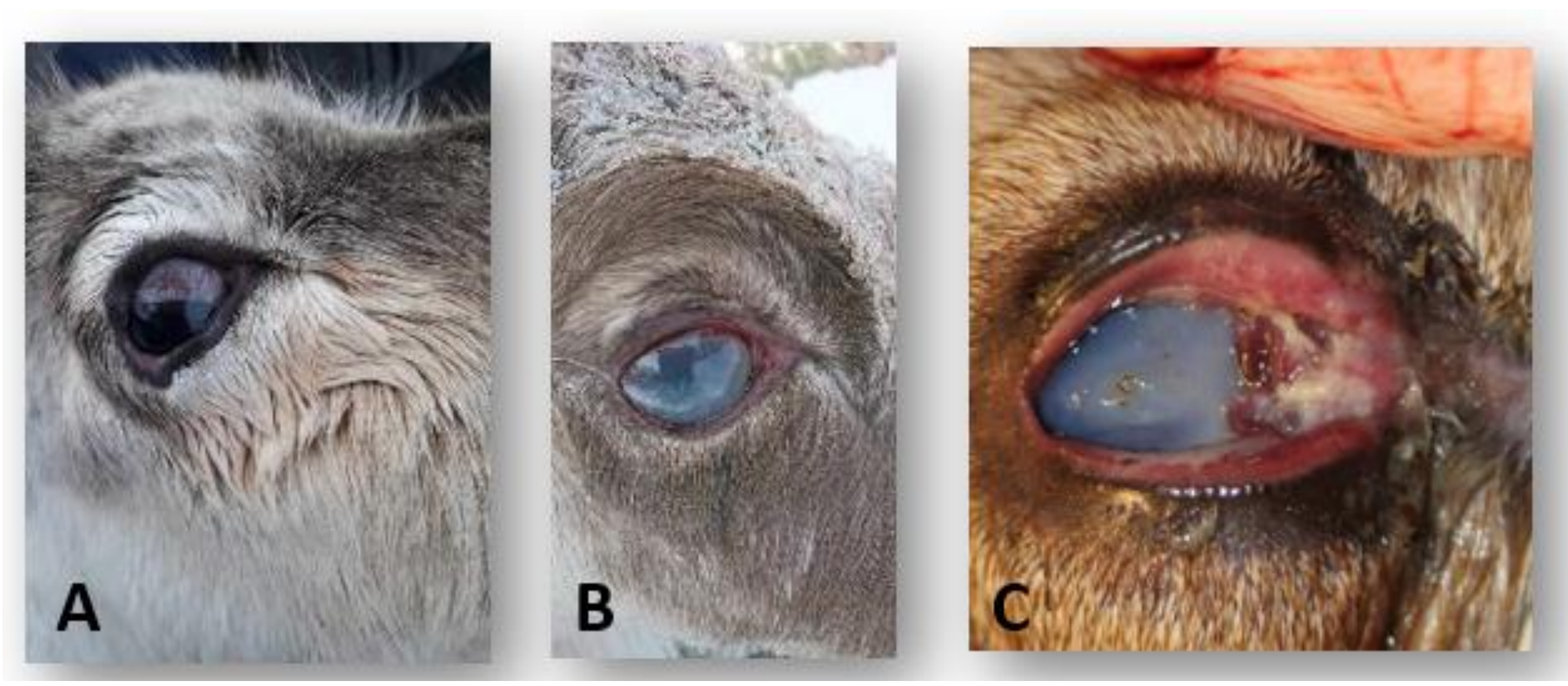

28) Lea go dus / siiddas árbevirolaš sámegiel namma daidda vigiide/rievdamiidda maid oainnát govain?

- ◯ Jua, čális/čilges dás vulobealde
- ◯ In
- ◯ In dieđe

Denne informasjonen vises kun i forhåndsvisningen

Følgende betingelser må være oppfylt for at spørsmålet skal vises for respondenten:

If the question Har du sett liknande ögonförändringar som Bild 1. A-C visar på dina renar under de senaste 10 åren? contains any of these alternatives

- In dieđe / Earálágán čalbmerievdama, čilges dás vulobeale makkár

- Jua

29) Eará mearkkašumit:

A:

B:

C:

Eará čalbmerievdan

Denne informasjonen vises kun i forhåndsvisningen

Følgende betingelser må være oppfylt for at spørsmålet skal vises for respondenten:

If the question Har du sett liknande ögonförändringar som Bild 1. A-C visar på dina renar under de senaste 10 åren? contains any of these alternatives

- In dieđe / Earálágán čalbmerievdama, čilges dás vulobeale makkár
- Jua

30) Dovddat dehe dieđát go eará «dološ» árbevirolaš vugiid mot dálkkodit dákkár njoammu čalbmevigi/čalmmiid rievdama?

- Jus jua, čále/čilges dan dás vulobealde
- In
- In dieđe

Denne informasjonen vises kun i forhåndsvisningen

Følgende betingelser må være oppfylt for at spørsmålet skal vises for respondenten:

If the question Har du sett liknande ögonförändringar som Bild 1. A-C visar på dina renar under de senaste 10 åren? contains any of these alternatives

- In dieđe / Earálágán čalbmerievdama, čilges dás vulobeale makkár
- Jua

31) Eará mearkkašumit:

Denne informasjonen vises kun i forhåndsvisningen

Følgende betingelser må være oppfylt for at spørsmålet skal vises for respondenten:

If the question Har du sett liknande ögonförändringar som Bild 1. A-C visar på dina renar under de senaste 10 åren? contains any of these alternatives

- In dieđe / Earálágán čalbmerievdama, čilges dás vulobeale makkár
- In
- Jua

Dás joatkit gažaldagaiguin njálbmevigi (orf) birrarf

32) Jus dus leat, atte áinnas eambbo dieđuid njoammu čalbmevigiid/-rievdamiid birra dákko:

2. Njoammu njálbmevihki/-vuorri (orf virus):

Govain 2. A-B:

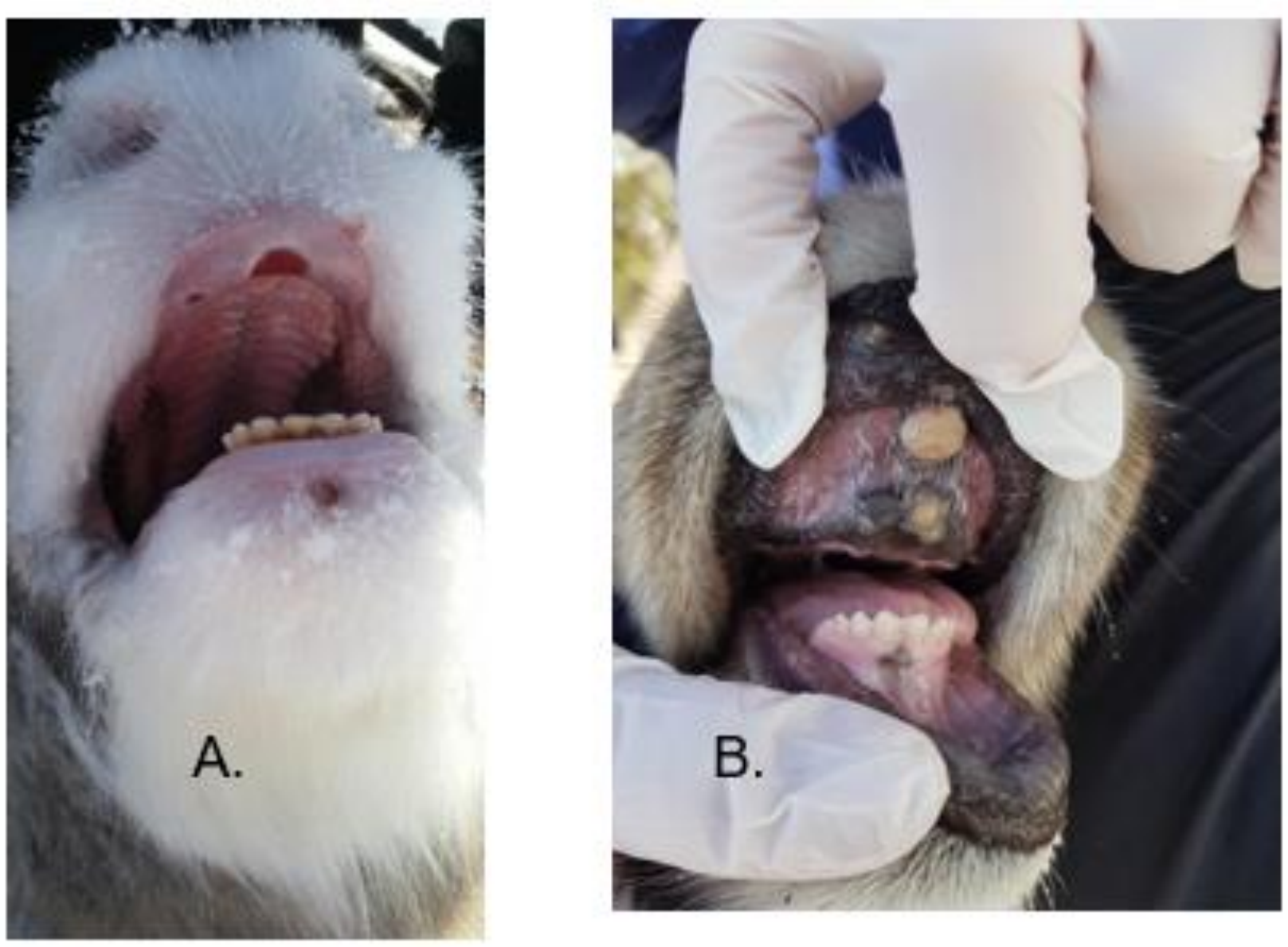

A: Ráigánan buogu, skoavhli njálmmis ja baksamis man orf virus lea dagahan. Dávda álgodásis.

B: Buogut, skoavhllit njálmmis ja bátneoaččis. Dávda guhkálmastan.

33) * Leat go don dan maŋemus 10 jagiin oaidnán sullasaš mearkkaid/rievdamiid iežat bohccuin go dat mat leat govas 2. A-B?

- Jua
- In
- In dieđe

Denne informasjonen vises kun i forhåndsvisningen

Følgende betingelser må være oppfylt for at spørsmålet skal vises for respondenten:

If the question Har du sett liknande förändringar som Bild 2. A-B visar på dina renar under de senaste 10 åren? contains any of these alternatives

- In dieđe
- Jua

Njoammu njálbmevihki/-vuorri (orf virus)

Govain 2. A-B:

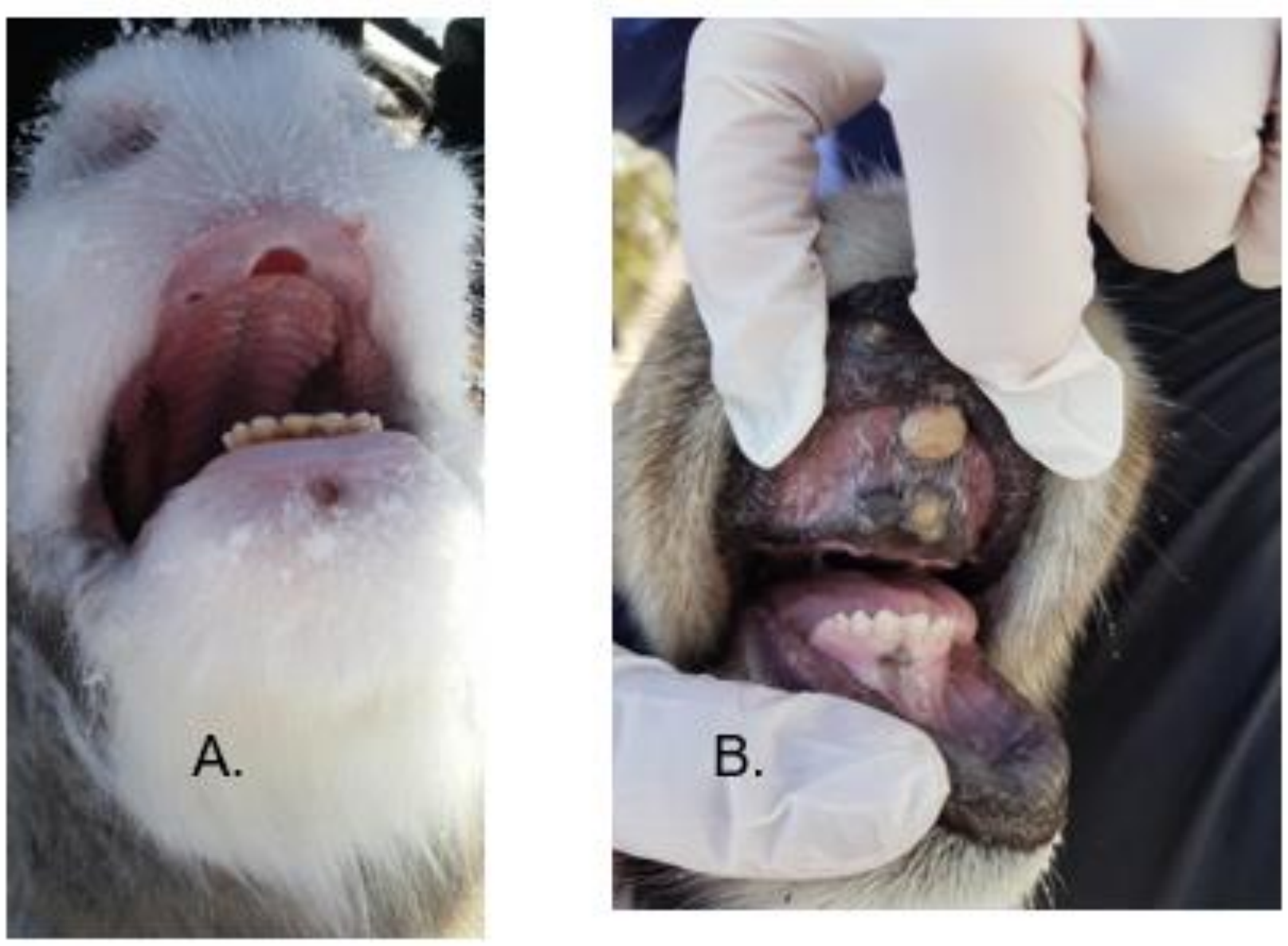

34) Goas oidnet maŋemus dakkár sullasaš mearkkaid/rievdamiid go mat leat govain 2. A-B?

- ◯ Dán maŋemus jagi
- ◯ Ii dan maŋemus jagi, muhto vuollel 5 jagi áigi.
- ◯ Eanet og 5 jagi áigi
- ◯ In dieđe

Denne informasjonen vises kun i forhåndsvisningen

Følgende betingelser må være oppfylt for at spørsmålet skal vises for respondenten:

If the question Har du sett liknande förändringar som Bild 2. A-B visar på dina renar under de senaste 10 åren? contains any of these alternatives

- In dieđe
- Jua

Njoammu njálbmevihki/-vuorri (orf virus)

35) Guđe áiggi jagis leat oaidnán eanemusat bohccuid main lea njálbmevihki/-vuorri (orf)? Merke galle miesi, nuorra bohcco ja rávis bohcco. Lea vejolaš merket máŋga jahkeáiggi.

	In leat oaidnán/ vuohttán dávdda	Giđđat	Geasset	Čakčat	Dálvet	Birra jagi (loahppalohku)
Miesit (vuollel jagi)	☐	☐	☐	☐	☐	☐
Nuorra bohccot (1-3 jagi)	☐	☐	☐	☐	☐	☐
Rávis bohccot (boarrásit go 3 jagi)	☐	☐	☐	☐	☐	☐

Denne informasjonen vises kun i forhåndsvisningen

Følgende betingelser må være oppfylt for at spørsmålet skal vises for respondenten:

If the question Har du sett liknande förändringar som Bild 2. A-B visar på dina renar under de senaste 10 åren? contains any of these alternatives

- In dieđe
- Jua

36) Eará mearkkašumit:

Denne informasjonen vises kun i forhåndsvisningen

Følgende betingelser må være oppfylt for at spørsmålet skal vises for respondenten:

If the question Har du sett liknande förändringar som Bild 2. A-B visar på dina renar under de senaste 10 åren? contains any of these alternatives

- In dieđe
- Jua

Njoammu njálbmevihki/-vuorri (orf virus)

37) Muital, juohke jagi áigodaga dáfus dás vulobealde, gos lei eallu/bohccot dalle go njálbmevihki/-vuorri (orf) fuomášuvvui. Dehe jus bohccot eai dohppehallan obanassiige dakkár vihkái. Vigi ihtin mearkkaša dan go vigivuolis bohccuid lohku lassána eambbo muhtun áiggi siste, ovdamearkka dihte dalle go dihto áiggi jagis eambbo bohccot go dábálaččat oččohallet vihkái, dehe muhtun oanehit áiggis oččohallet vihkái, omd. muhtun vahkuid siste.

	In leat oaidnán/ vuohttán dávdda	Dohppehalle gárddis	Dohppehalle dalle go bibmojuvvo lávda ealus/luovos dievás	Dohppehalle lávda/ čoahkke ealu/ luovos dievá guohtuma oktavuođas
2015/2016	☐	☐	☐	☐
2016/2017	☐	☐	☐	☐
2017/2018	☐	☐	☐	☐
2018/2019	☐	☐	☐	☐
2019/2020	☐	☐	☐	☐
2020/2021	☐	☐	☐	☐

Denne informasjonen vises kun i forhåndsvisningen

Følgende betingelser må være oppfylt for at spørsmålet skal vises for respondenten:

If the question Har du sett liknande förändringar som Bild 2. A-B visar på dina renar under de senaste 10 åren? contains any of these alternatives

- In dieđe
- Jua

38) Eará mearkkašumit:

Denne informasjonen vises kun i forhåndsvisningen

Følgende betingelser må være oppfylt for at spørsmålet skal vises for respondenten:

If the question Har du sett liknande förändringar som Bild 2. A-B visar på dina renar under de senaste 10 åren? contains any of these alternatives

- In dieđe
- Jua

Njoammu njálbmevihki/-vuorri (orf virus)

39) Sullii man galle bohccos ealus /siiddas lei vihki dan maŋemus háve dalle go bohccot dohppehalle njálbmevihkái /-vuorrái (orf)? Muital vuos álggos galle bohcco dohppehalle vihkái, jus leat vásihan dan.

Galle miesi (jahkásaččat dehe nuorat)

Galle nuorra bohcco (1-3 jagi)

Galle rávis bohcco (boarrásit og 3 jagi)

Oktiibuot galle bohcco siiddas/ealus (sihke dearvvaš ja skihpa/dávdavuolis)

Denne informasjonen vises kun i forhåndsvisningen

Følgende betingelser må være oppfylt for at spørsmålet skal vises for respondenten:

If the question Har du sett liknande förändringar som Bild 2. A-B visar på dina renar under de senaste 10 åren? contains any of these alternatives

- In dieđe
- Jua

40) Eará mearkkašumit:

Denne informasjonen vises kun i forhåndsvisningen

Følgende betingelser må være oppfylt for at spørsmålet skal vises for respondenten:

If the question Har du sett liknande förändringar som Bild 2. A-B visar på dina renar under de senaste 10 åren? contains any of these alternatives

- In dieđe
- Jua

Njoammu njálbmevihki/-vuorri (orf virus)

41) * Leat go mearkkašan lea go bohccuid lohku, mat leat dohppehallan njálbmevihkái/-vuorrái, rievdan dan maŋemus 5 jagis?

- Jua, dat leat lassánan
- Jua, dat lea unnon
- In, lohku ii leat lassánan iige unnon
- In dieđe

Denne informasjonen vises kun i forhåndsvisningen

Følgende betingelser må være oppfylt for at spørsmålet skal vises for respondenten:

If the question Har du sett liknande förändringar som Bild 2. A-B visar på dina renar under de senaste 10 åren? contains any of these alternatives

- In dieđe
- Jua

og

If the question Upplever du att antalet renar som drabbats av munvårtsjuka/orf har förändrats över de senaste 5 åren? contains any of these alternatives

- Jua, dat leat lassánan

Njoammu njálbmevihki/-vuorri (orf virus)

42) Maid doaivvut leat sivvan dasa go lohku lea lassánan?

Denne informasjonen vises kun i forhåndsvisningen

Følgende betingelser må være oppfylt for at spørsmålet skal vises for respondenten:

If the question Har du sett liknande förändringar som Bild 2. A-B visar på dina renar under de senaste 10 åren? contains any of these alternatives

- In dieđe
- Jua

og

If the question Upplever du att antalet renar som drabbats av munvårtsjuka/orf har förändrats över de senaste 5 åren? contains any of these alternatives

- Jua, dat lea unnon

Njoammu njálbmevihki/-vuorri (orf virus)

43) Maid doaivvut leat sivvan dasa go lohku lea unnon?

Denne informasjonen vises kun i forhåndsvisningen

Følgende betingelser må være oppfylt for at spørsmålet skal vises for respondenten:

If the question Har du sett liknande förändringar som Bild 2. A-B visar på dina renar under de senaste 10 åren? contains any of these alternatives

- In dieđe
- Jua

Njoammu njálbmevihki/-vuorri (orf virus)

44) * Álggahuvvojit go dábálaččat makkárge doaimmat/dálkkodeamit (omd. váldit bohccuid sierra, njuovat dehe gieđahalat eará ládje) go fuomášuvvo bohccuin njálbmevihki/-vuorri?

- ○ Jua
- ○ In
- ○ In dieđe

Denne informasjonen vises kun i forhåndsvisningen

Følgende betingelser må være oppfylt for at spørsmålet skal vises for respondenten:

If the question Har du sett liknande förändringar som Bild 2. A-B visar på dina renar under de senaste 10 åren? contains any of these alternatives

- In dieđe
- Jua

45) Makkár doaibma/dálkkodeapmi álggahuvvui dalle maŋemus go fuomášuvvui bohccuin njálbmevihki/-vuorri (orf)? Merke ovtta/máŋga doaibmavuogi/dálkkodeami mii álggahuvvui:

☐In bargan/čađahan maidege
☐Skihpa/dávdavuolis bohccot sirrejuvvo sierra gárdái
☐Dieđihuvvui šibitdoaktárii
☐Penicilliidnadálkkodeapmi álggahuvvui
☐Njuvvojuvvo
☐Bohccot ráddjojuvvo (goddojuvvo) eret/bálkestuvvo
☐Bohccot dálkkoduvvo eará dálkasiiguin, čilges mot ja mainna dás vulobealde
☐Eará, čilge dás vuolábealde maid ja mot

Denne informasjonen vises kun i forhåndsvisningen

Følgende betingelser må være oppfylt for at spørsmålet skal vises for respondenten:

If the question Har du sett liknande förändringar som Bild 2. A-B visar på dina renar under de senaste 10 åren? contains any of these alternatives

- In dieđe
- Jua

46) Eará mearkkašumit:

Denne informasjonen vises kun i forhåndsvisningen

Følgende betingelser må være oppfylt for at spørsmålet skal vises for respondenten:

If the question Har du sett liknande förändringar som Bild 2. A-B visar på dina renar under de senaste 10 åren? contains any of these alternatives

- In dieđe
- Jua

Njoammu njálbmevihki/-vuorri (orf virus)

47) Lea go dávdi dohppehallan dehe dávdii dohppehallan lohku (orf) váikkuhan dutnje ruđalaččat?

- Jua
- In
- In dieđe

Denne informasjonen vises kun i forhåndsvisningen

Følgende betingelser må være oppfylt for at spørsmålet skal vises for respondenten:

If the question Har du sett liknande förändringar som Bild 2. A-B visar på dina renar under de senaste 10 åren? contains any of these alternatives

- In dieđe
- Jua

48) Eará mearkkašumit:

Denne informasjonen vises kun i forhåndsvisningen

Følgende betingelser må være oppfylt for at spørsmålet skal vises for respondenten:

If the question Har du sett liknande förändringar som Bild 2. A-B visar på dina renar under de senaste 10 åren? contains any of these alternatives

- In dieđe
- Jua

Njoammu njálbmevihki/-vuorri (orf virus)

Govain 2. A-B:

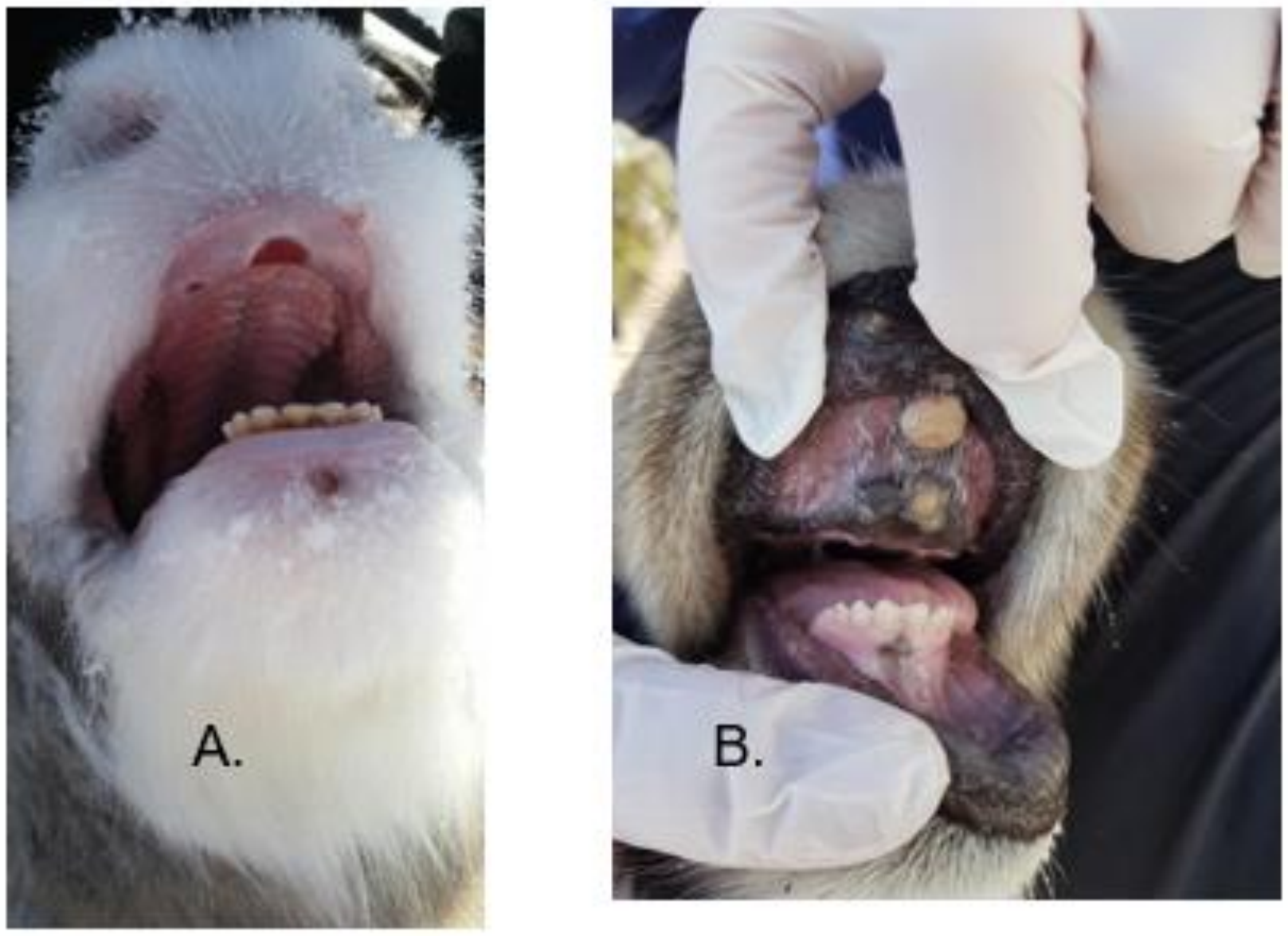

49) Lea go dus / siiddas árbevirolaš sámegiel namma daidda vigiide/rievdamiidda maid oainnát govain?

- Jua, čális/čilges dás vulobealde
- In
- In dieđe

Denne informasjonen vises kun i forhåndsvisningen

Følgende betingelser må være oppfylt for at spørsmålet skal vises for respondenten:

If the question Har du sett liknande förändringar som Bild 2. A-B visar på dina renar under de senaste 10 åren? contains any of these alternatives

- In dieđe
- Jua

50) Eará mearkkašumit:

Denne informasjonen vises kun i forhåndsvisningen

Følgende betingelser må være oppfylt for at spørsmålet skal vises for respondenten:

If the question Har du sett liknande förändringar som Bild 2. A-B visar på dina renar under de senaste 10 åren? contains any of these alternatives

- In dieđe

- Jua

51) Dovddat dehe dieđát go eará «dološ» árbevirolaš vugiid mot dálkkodit dákkár njálbmevigi/-vuorri (orf)?

- ◯ Jua, čilges dás vulobealde
- ◯ In
- ◯ In dieđe

Denne informasjonen vises kun i forhåndsvisningen

Følgende betingelser må være oppfylt for at spørsmålet skal vises for respondenten:

If the question Har du sett liknande förändringar som Bild 2. A-B visar på dina renar under de senaste 10 åren? contains any of these alternatives

- In dieđe
- Jua

52) Eará mearkkašumit:

Denne informasjonen vises kun i forhåndsvisningen

Følgende betingelser må være oppfylt for at spørsmålet skal vises for respondenten:

If the question Har du sett liknande förändringar som Bild 2. A-B visar på dina renar under de senaste 10 åren? contains any of these alternatives

- In dieđe
- In
- Jua

Dás joatkit gažaldagaiguin njunnevigi (oral nekrobacillose) birra

53) Jus dus leat, atte áinnas eambbo dieđuid njálbmevigi (orf) birra dákko:

3. Njunnevihki / oral nekrobacillose:

Govain 3. A-C:

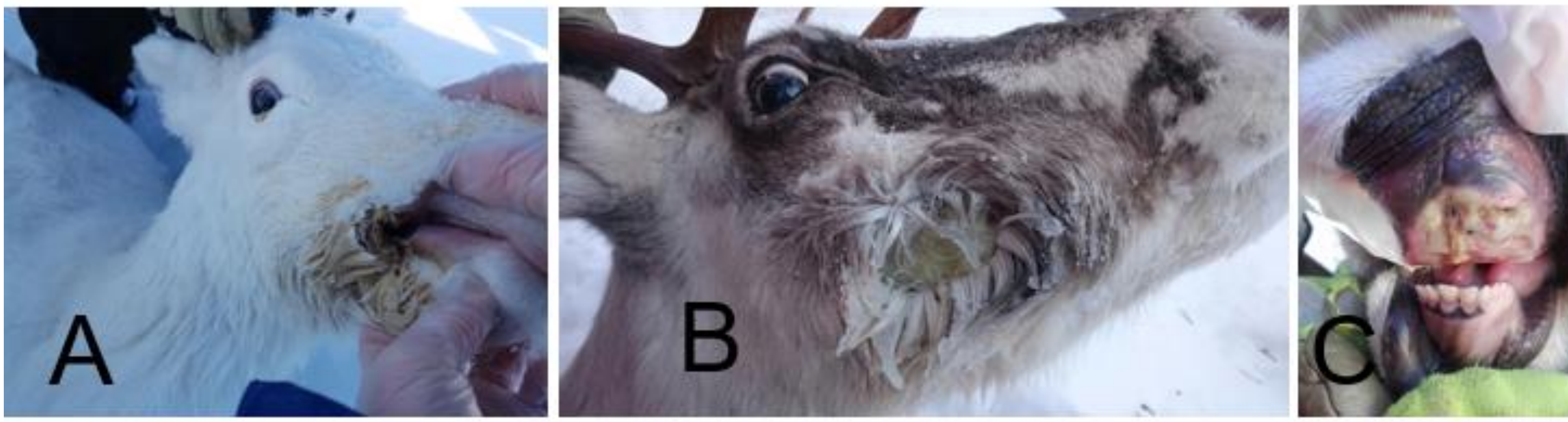

A: Beđđon buogu ja siedja golgan njálbmegeahčái.

B: Buogu, muohtovuolši mii lea dagahan bistevaš ráiggi muhtui.

C: Buogu, sidjon vuolši bátneoaččis.

54) * Leat go don dan maŋemus 10 jagiin oaidnán sullasaš mearkkaid/rievdamiid iežat bohccuin go dat mat leat govain 3. A-C?

- Jua
- In
- In dieđe

Denne informasjonen vises kun i forhåndsvisningen

Følgende betingelser må være oppfylt for at spørsmålet skal vises for respondenten:

If the question Har du sett liknande förändringar som Bild 3. A-C visar på dina renar under de senaste 10 åren? contains any of these alternatives

- In dieđe
- Jua

Njunnevihki / oral nekrobacillose:

Govain 3. A-C:

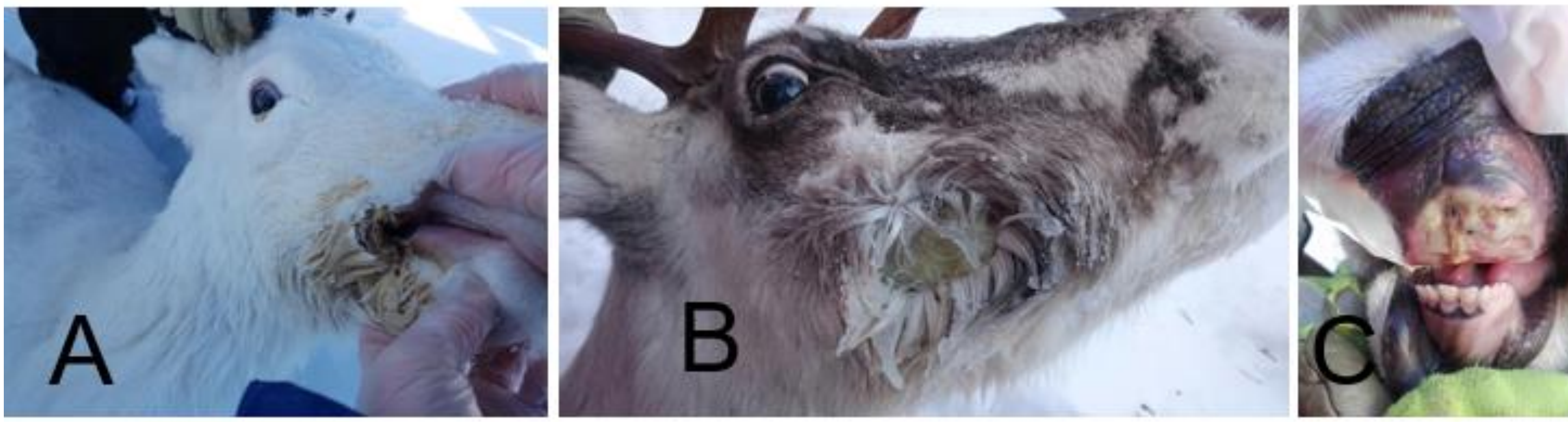

55) Goas oidnet maŋemus dakkár sullasaš dávdamearkkaid mat leat govain 3. A-C?

- ◯ Dán maŋemus jagi
- ◯ Ii dan maŋemus jagi, muhto vuollel 5 jagi áigi.
- ◯ Eanet og 5 jagi áigi
- ◯ In dieđe

Denne informasjonen vises kun i forhåndsvisningen

Følgende betingelser må være oppfylt for at spørsmålet skal vises for respondenten:

If the question Har du sett liknande förändringar som Bild 3. A-C visar på dina renar under de senaste 10 åren? contains any of these alternatives

- In dieđe
- Jua

Njunnevihki / oral nekrobacillose:

56) Guđe áiggi jagis oainnát eanemusat bohccuid main lea njunnevihki? Merke misiid, nuorra bohccuid ja rávis bohccuid dáfus. Lea vejolaš merket máŋga jahkeáiggi.

	In oaidnán/ vuohttán	Giđđat	Geasset	Čakčat	Dálvet	Birra jagi (loahppalohku)
Miesit (jahkásaš dehe vuollel jagi)	☐	☐	☐	☐	☐	☐
Nuorra bohccot (1-3 jagi)	☐	☐	☐	☐	☐	☐
Rávis bohccot (boarrásit go 3 jagi)	☐	☐	☐	☐	☐	☐

Denne informasjonen vises kun i forhåndsvisningen

Følgende betingelser må være oppfylt for at spørsmålet skal vises for respondenten:

If the question Har du sett liknande förändringar som Bild 3. A-C visar på dina renar under de senaste 10 åren? contains any of these alternatives

- In dieđe
- Jua

57) Eará mearkkašumit:

Denne informasjonen vises kun i forhåndsvisningen

Følgende betingelser må være oppfylt for at spørsmålet skal vises for respondenten:

If the question Har du sett liknande förändringar som Bild 3. A-C visar på dina renar under de senaste 10 åren? contains any of these alternatives

- In dieđe
- Jua

Njunnevihki / oral nekrobacillose:

58) Muital, juohke jagi áigodaga dáfus dás vulobealde, gos lei eallu/bohccot dalle go njunnevihki fuomášuvvui. Dehe jus bohccot eai dohppehallan obanassiige dakkár vihkái. Vigi ihtin mearkkaša dan go vigivuolis bohccuid lohku lassána eambbo muhtun áiggi siste, ovdamearkka dihte dalle go dihto áiggi jagis eambbo bohccot go dábálaččat oččohallet vihkái, dehe muhtun oanehit áiggis oččohallet vihkái, omd. muhtun vahkuid siste.

	In leat oaidnán/ vuohttán dávdda	Dohppehalle gárddis	Dohppehalle dalle go lávda/čoahkke eallu /luovos dievás bibmojuvvui	Dohppehalle lávda/ čoahkke ealu/ luovos dievá guohtuma oktavuođas
2015/2016	☐	☐	☐	☐
2016/2017	☐	☐	☐	☐
2017/2018	☐	☐	☐	☐
2018/2019	☐	☐	☐	☐

	In leat oaidnán/ vuohttán dávdda	Dohppehalle gárddis	Dohppehalle dalle go lávda/čoahkke eallu /luovos dievás bibmojuvvui	Dohppehalle lávda/ čoahkke ealu/ luovos dievá guohtuma oktavuođas
2019/2020	☐	☐	☐	☐
2020/2021	☐	☐	☐	☐

Denne informasjonen vises kun i forhåndsvisningen

Følgende betingelser må være oppfylt for at spørsmålet skal vises for respondenten:

If the question Har du sett liknande förändringar som Bild 3. A-C visar på dina renar under de senaste 10 åren? contains any of these alternatives

- In dieđe
- Jua

59) Eará mearkkašumit:

Denne informasjonen vises kun i forhåndsvisningen

Følgende betingelser må være oppfylt for at spørsmålet skal vises for respondenten:

If the question Har du sett liknande förändringar som Bild 3. A-C visar på dina renar under de senaste 10 åren? contains any of these alternatives

- In dieđe
- Jua

Njunnevihki / oral nekrobacillose:

60) Sullii man galle bohccos ealus /siiddas lei vihki dan maŋemus háve dalle go bohccot dohppehalle njunnevihkái? Muital vuos álggos galle bohcco dohppehalle vihkái, jus leat vásihan dan.

Galle miesi (jahkásaččat dehe nuorat)

Galle nuorra bohcco (1-3 jagi)

Galle rávis bohcco (boarrásit og 3 jagi)

Oktiibuot galle bohcco siiddas/ealus (sihke dearvvaš ja skihpa/dávdavuolis)

Denne informasjonen vises kun i forhåndsvisningen

Følgende betingelser må være oppfylt for at spørsmålet skal vises for respondenten:

If the question Har du sett liknande förändringar som Bild 3. A-C visar på dina renar under de senaste 10 åren? contains any of these alternatives

- In dieđe
- Jua

61) Eará mearkkašumit:

Denne informasjonen vises kun i forhåndsvisningen

Følgende betingelser må være oppfylt for at spørsmålet skal vises for respondenten:

If the question Har du sett liknande förändringar som Bild 3. A-C visar på dina renar under de senaste 10 åren? contains any of these alternatives

- In dieđe
- Jua

Njunnevihki / oral nekrobacillose:

62) * Leat go mearkkašan lea go bohccuid lohku, mat leat dohppehallon njunnevihkái, rievdan dan maŋemus 5 jagis?

- ○ Jua, dat leat lassánan
- ○ Jua, dat lea unnon
- ○ In, lohku ii leat lassánan iige unnon
- ○ In dieđe

Denne informasjonen vises kun i forhåndsvisningen

Følgende betingelser må være oppfylt for at spørsmålet skal vises for respondenten:

If the question Har du sett liknande förändringar som Bild 3. A-C visar på dina renar under de senaste 10 åren? contains any of these alternatives

- In dieđe
- Jua

og

If the question Upplever du att antalet renar som drabbats av munröta/oral nekrobacillos/njunnevikke har förändrats över de senaste fem åren? contains any of these alternatives

- Jua, dat leat lassánan

Njunnevihki / oral nekrobacillose:

63) Maid doaivvut leat sivvan dasa go lohku lea lassánan?

Denne informasjonen vises kun i forhåndsvisningen

Følgende betingelser må være oppfylt for at spørsmålet skal vises for respondenten:

If the question Har du sett liknande förändringar som Bild 3. A-C visar på dina renar under de senaste 10 åren? contains any of these alternatives

- In dieđe
- Jua

og

If the question Upplever du att antalet renar som drabbats av munröta/oral nekrobacillos/njunnevikke har förändrats över de senaste fem åren? contains any of these alternatives

- Jua, dat lea unnon

Njunnevihki / oral nekrobacillose:

64) Maid doaivvut leat sivvan dasa go lohku lea unnon?

Denne informasjonen vises kun i forhåndsvisningen

Følgende betingelser må være oppfylt for at spørsmålet skal vises for respondenten:

If the question Har du sett liknande förändringar som Bild 3. A-C visar på dina renar under de senaste 10 åren? contains any of these alternatives

- In dieđe
- Jua

Njunnevihki / oral nekrobacillose:

65) * Álggahuvvojit go dábálaččat makkárge doaimmat/dálkkodeamit (omd. váldit bohccuid sierra, njuovat dehe gieđahalat eará ládje) go fuomášuvvo bohccuin njunnevihki?

- ○ Jua
- ○ In
- ○ In dieđe

Denne informasjonen vises kun i forhåndsvisningen

Følgende betingelser må være oppfylt for at spørsmålet skal vises for respondenten:

If the question Har du sett liknande förändringar som Bild 3. A-C visar på dina renar under de senaste 10 åren? contains any of these alternatives

- In dieđe
- Jua

66) Makkár doaibma/dálkkodeapmi álggahuvvui dalle maŋemus go fuomášuvvui bohccuin njunnevihki?

- ☐ In bargan/čađahan maidege
- ☐ Skihpa/dávdavuolis bohccot sirrejuvvo sierra gárdái
- ☐ Dieđihuvvui šibitdoaktárii
- ☐ Penicilliidnadálkkodeapmi álggahuvvui
- ☐ Njuvvojuvvo
- ☐ Bohccot ráddjojuvvo (goddojuvvo) eret/bálkestuvvo
- ☐ Dálkkoduvvo eará dálkasiiguin, čilges mot ja mainna dás vulobealde
- ☐ Eará, čilge dás vuolábealde maid ja mot:

Denne informasjonen vises kun i forhåndsvisningen

Følgende betingelser må være oppfylt for at spørsmålet skal vises for respondenten:

If the question Har du sett liknande förändringar som Bild 3. A-C visar på dina renar under de senaste 10 åren? contains any of these alternatives

- In dieđe

- Jua

67) Eará mearkkašumit:

Denne informasjonen vises kun i forhåndsvisningen

Følgende betingelser må være oppfylt for at spørsmålet skal vises for respondenten:

If the question Har du sett liknande förändringar som Bild 3. A-C visar på dina renar under de senaste 10 åren? contains any of these alternatives

- In dieđe
- Jua

Njunnevihki / oral nekrobacillose:

68) Lea go njunnedávdi dohppehallan dehe njunnedávdii dohppehallan lohku váikkuhan dunnje ruđalaččat?

- ○ Jua
- ○ In
- ○ In dieđe

Denne informasjonen vises kun i forhåndsvisningen

Følgende betingelser må være oppfylt for at spørsmålet skal vises for respondenten:

If the question Har du sett liknande förändringar som Bild 3. A-C visar på dina renar under de senaste 10 åren? contains any of these alternatives

- In dieđe
- Jua

69) Eará mearkkašumit:

Denne informasjonen vises kun i forhåndsvisningen

Følgende betingelser må være oppfylt for at spørsmålet skal vises for respondenten:

If the question Har du sett liknande förändringar som Bild 3. A-C visar på dina renar under de senaste 10 åren? contains any of these alternatives

- In dieđe
- Jua

Njunnevihki / oral nekrobacillose:

Govain 3. A-C:

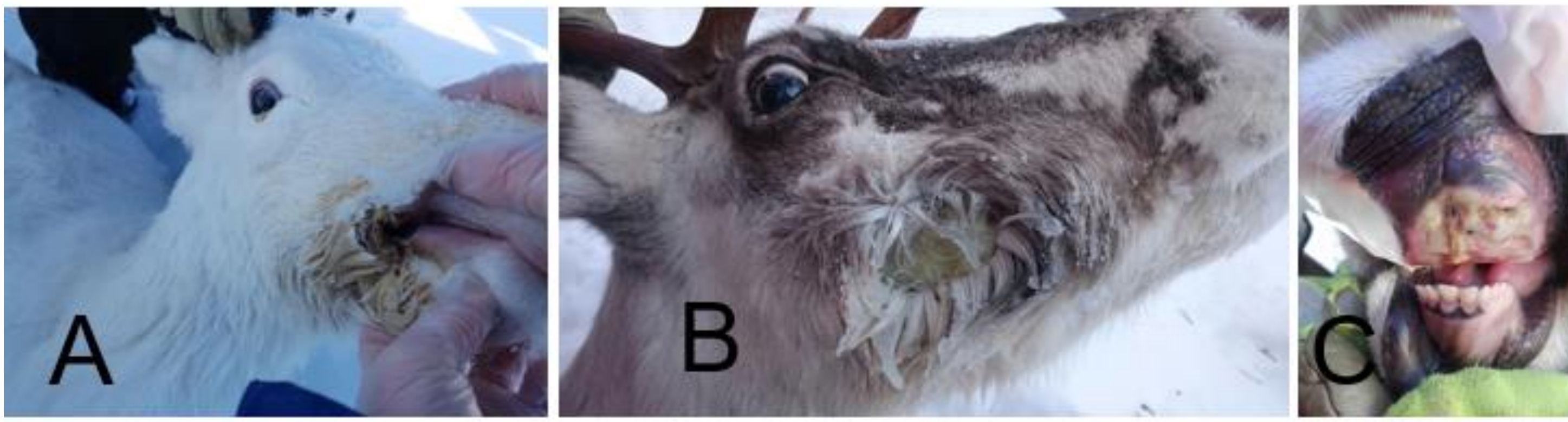

70) Lea go dus / siiddas árbevirolaš sámegiel namma daidda vigiide/rievdamiidda maid oainnát govain?

- ○ Jua, čális/čilges dás vulobealde
- ○ Ii
- ○ In dieđe

Denne informasjonen vises kun i forhåndsvisningen

Følgende betingelser må være oppfylt for at spørsmålet skal vises for respondenten:

If the question Har du sett liknande förändringar som Bild 3. A-C visar på dina renar under de senaste 10 åren? contains any of these alternatives

- In dieđe
- Jua

71) Eará mearkkašumit:

Denne informasjonen vises kun i forhåndsvisningen

Følgende betingelser må være oppfylt for at spørsmålet skal vises for respondenten:

If the question Har du sett liknande förändringar som Bild 3. A-C visar på dina renar under de senaste 10 åren? contains any of these alternatives

- In dieđe
- Jua

72) Dovddat dehe dieđát go eará «dološ» árbevirolaš vugiid mot dálkkodit dákkár njunnevigi?

- Jua, čilges dás vulobealde
- In
- In dieđe

Denne informasjonen vises kun i forhåndsvisningen

Følgende betingelser må være oppfylt for at spørsmålet skal vises for respondenten:

If the question Har du sett liknande förändringar som Bild 3. A-C visar på dina renar under de senaste 10 åren? contains any of these alternatives

- In dieđe
- Jua

73) Eará mearkkašumit:

Denne informasjonen vises kun i forhåndsvisningen

Følgende betingelser må være oppfylt for at spørsmålet skal vises for respondenten:

If the question Har du sett liknande förändringar som Bild 3. A-C visar på dina renar under de senaste 10 åren? contains any of these alternatives

- In dieđe
- In
- Jua

Njunnevihki / nekrobacillose bohcco čovjjiin:

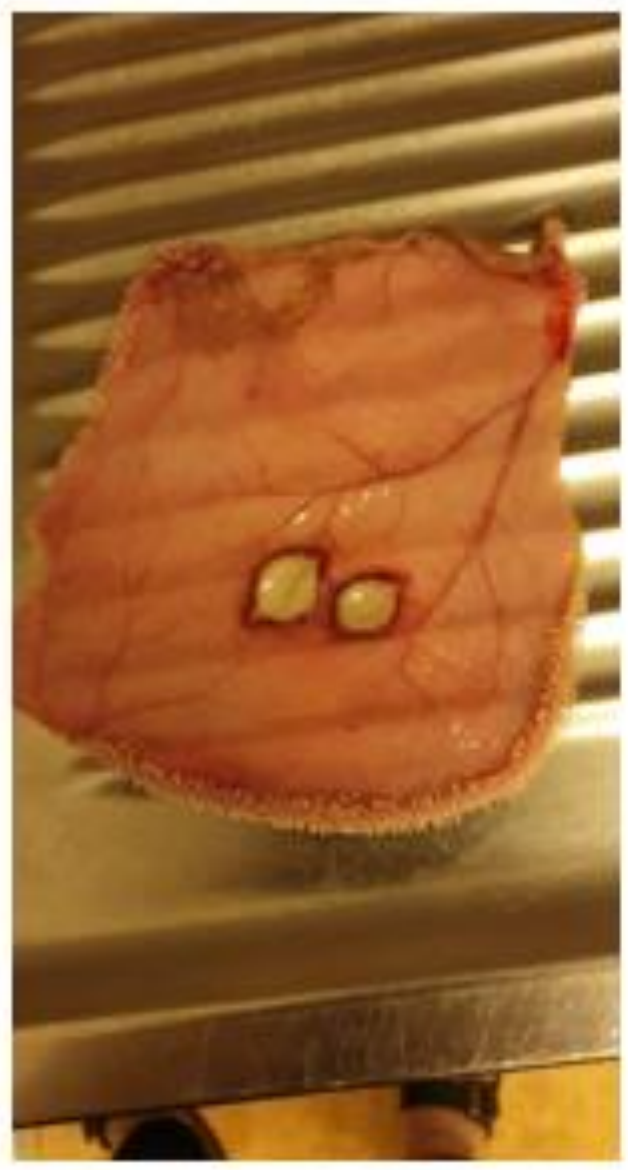 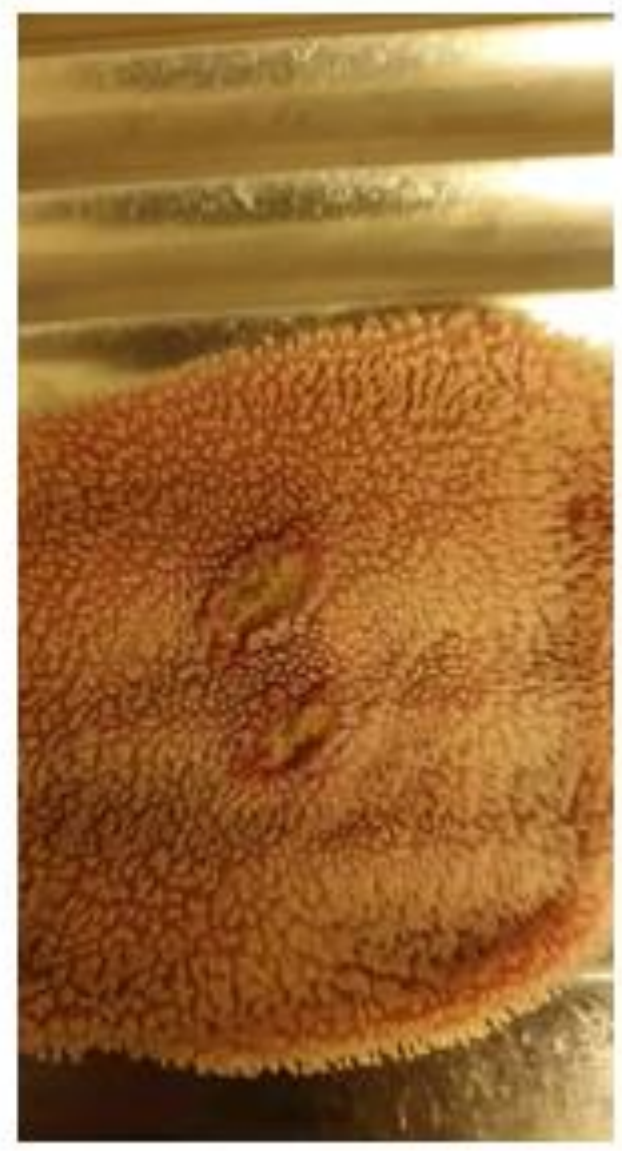

74) Leat go vásihan dakkár mearkkaid/rievdamiid, maid oainnát govain dás bajábeale govain, iežat bohccuid čovjjiin (go leat njuovvan/iskan sivaid)?

- Jua
- In
- In dieđe

Denne informasjonen vises kun i forhåndsvisningen

Følgende betingelser må være oppfylt for at spørsmålet skal vises for respondenten:

If the question Har du sett liknande förändringar som Bild 3. A-C visar på dina renar under de senaste 10 åren? contains any of these alternatives

- In dieđe
- In
- Jua

Dás joatkit gažaldagaiguin bohccuid eará dávddaid/vigiid birra

75) Jus dus leat, atte áinnas eambbo dieđuid njunnevigi birra dákko:

4. Gažaldagat bohccuid eará vigiid/dávddaid birra:

76) * Makkár vigiid leat oaidnán bohccuin 2019/2020 dálvvis gitta dássážii? Merke buot vástádusaid mat heivejit dasa.

- ☐ Reiton / Olguleapmi
- ☐ Siigan /jorgásan miessegoahti
- ☐ Livzá/livzzas / skierbmun / roavvi
- ☐ Váibbat
- ☐ Čáhcečoavji / Njuoskaoaččát čoavjjevuolli
- ☐ Lužohat / njárbes čoavji
- ☐ Čáhce-/Skulči/duški čoavji (čoalit skuhraidit) - (njárbes, čáhcešliivi čoavjjis)
- ☐ Baggan / rippágan /obbonvihki
- ☐ Oaivevuorri /oaivevuorahat
- ☐ Luhtehasat/parasihtat (čielgeboaru/gurbmá, njunneboaru/sávlagat, čoalleparasihtat, náhkke/guolgaparasihtat)
- ☐ Jápmán bohccot main eai leat dihto vigit/dávddat
- ☐ Eará, čális mearkkašumiid vuolláí dás vulobealde
- ☐ In guđege namuhuvvon vigiin/dávddain

77) Eará mearkkašumit:

Denne informasjonen vises kun i forhåndsvisningen

Følgende betingelser må være oppfylt for at spørsmålet skal vises for respondenten:

If the question Vilka av följande sjukdomar har du observerat från vintersäsongen 2019/2020 fram till idag? Ange alla som observerats. contains any of these alternatives

- Čáhcečoavji / Njuoskaoaččát čoavjjevuolli

78) Mainna bibmet dan bohcco ovdal go oaččui čáhcečoavjji?

- ○ Duše pelletsiiguin
- ○ Duše siilosuinniiguin/suvrasuinniiguin
- ○ Sihke pellets + siilosuinniiguin/suvrasuinniiguin
- ○ In biebman mainnage
- ○ Eará: čális dás vuolábealde mearkkašupmái.

Denne informasjonen vises kun i forhåndsvisningen

Følgende betingelser må være oppfylt for at spørsmålet skal vises for respondenten:

If the question Vilka av följande sjukdomar har du observerat från vintersäsongen 2019/2020 fram till idag? Ange alla som observerats. contains any of these alternatives

- Čáhcečoavji / Njuoskaoaččát čoavjjevuolli

79) Eará mearkkašumit:

Denne informasjonen vises kun i forhåndsvisningen

Følgende betingelser må være oppfylt for at spørsmålet skal vises for respondenten:

If the question Vilka av följande sjukdomar har du observerat från vintersäsongen 2019/2020 fram till idag? Ange alla som observerats. contains any of these alternatives

- Čáhcečoavji / Njuoskaoaččát čoavjjevuolli

80) Leat go oaidnán eará/eanet dávdamearkkaid bohccuin mat leat ožžon čáhcečoavjji? Čilges.

Denne informasjonen vises kun i forhåndsvisningen

Følgende betingelser må være oppfylt for at spørsmålet skal vises for respondenten:

81) Oaččut go šibitdoaktáris dan veahki maid dárbbašat?

- Jua
- In, namut/čális dás vulobealde manne it

Denne informasjonen vises kun i forhåndsvisningen

Følgende betingelser må være oppfylt for at spørsmålet skal vises for respondenten:

82) Eará mearkkašumit:

83) Leat go sádden muhtun jápmán bohccuid iskkadeapmái?

- ○ Jua
- ○ In

84) Leat go ieš jeavddalaččat/jámma iskkan jápmán bohcco šibitdoaktára vehkiin?

- ○ Jua
- ○ In

85) Eará mearkkašumit:

86) * Dálkkoduvvojit/boahkuhuvvojit go bohccot jeavddalaččat gurpmáid ja sávlagiid vuostá?

- ○ Jua
- ○ In

Denne informasjonen vises kun i forhåndsvisningen

Følgende betingelser må være oppfylt for at spørsmålet skal vises for respondenten:

If the question Behandlas renarna regelbundet mot Korm? contains any of these alternatives

- Jua

87) Goas jagis dálkkoduvvojit/boahkuhuvvojit bohccot gurpmáid ja/dehe sávlagiid vuostá?

- ☐ Giđđat
- ☐ Geasset
- ☐ Čakčat
- ☐ Dálvet

Denne informasjonen vises kun i forhåndsvisningen

Følgende betingelser må være oppfylt for at spørsmålet skal vises for respondenten:

If the question Behandlas renarna regelbundet mot Korm? contains any of these alternatives

- Jua

88) Makkár bohccot dálkkoduvvojit/boahkuhuvvojit dábálaččat gurpmáid ja/dehe sávlagiid vuostá?

- Dušše ealihanmiesit (jahkásaš dehe nuorat)
- Dušše álddut (badjel jahkásaččat)
- Dušše varrásat (badjel jahkásaččat)
- Sihke ealihanmiesit, varrásat ja álddut
- Eará, čilges makkár bohccot

Denne informasjonen vises kun i forhåndsvisningen

Følgende betingelser må være oppfylt for at spørsmålet skal vises for respondenten:

If the question Behandlas renarna regelbundet mot Korm? contains any of these alternatives

- Jua

89) Eará mearkkašumit:

Govain 4. A-B. Dábálaš nirana:

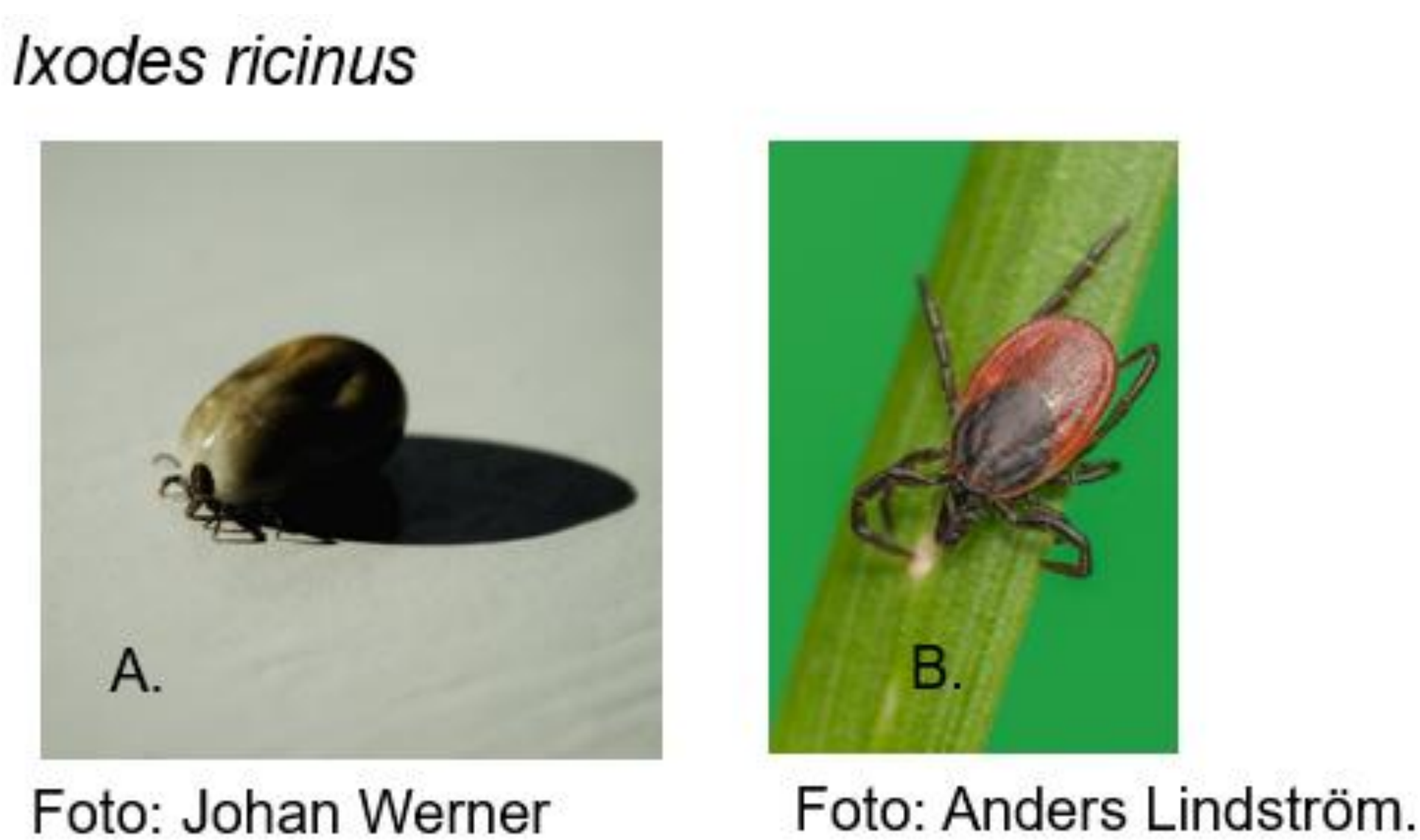

A: Njiŋŋelas niranas mii lea njamman vara.

B: Njiŋŋelas niranas suoinni nalde.

90) Niranas sáhttá buktit njoammuskibaid ja go šaddet dađistaga lieggasit dálkkit, de leavvá niranas maid davás guvlui. Leat go gávdnan/oaidnán nirranása iežat bohccuin (geahča 4. gova)?

- Jua
- In
- In dieđe

Dás joatkit gažaldagaiguin bohccuid biebmama birra

91) Jus dus leat, atte áinnas eambbo dieđuid eará vigiid/dávddaid mat leat bohccuin dákko:

5. Dál vel dás muhtun gažaldagat bohccuid biebmama birra ovdal go gearggat iskkademiin!

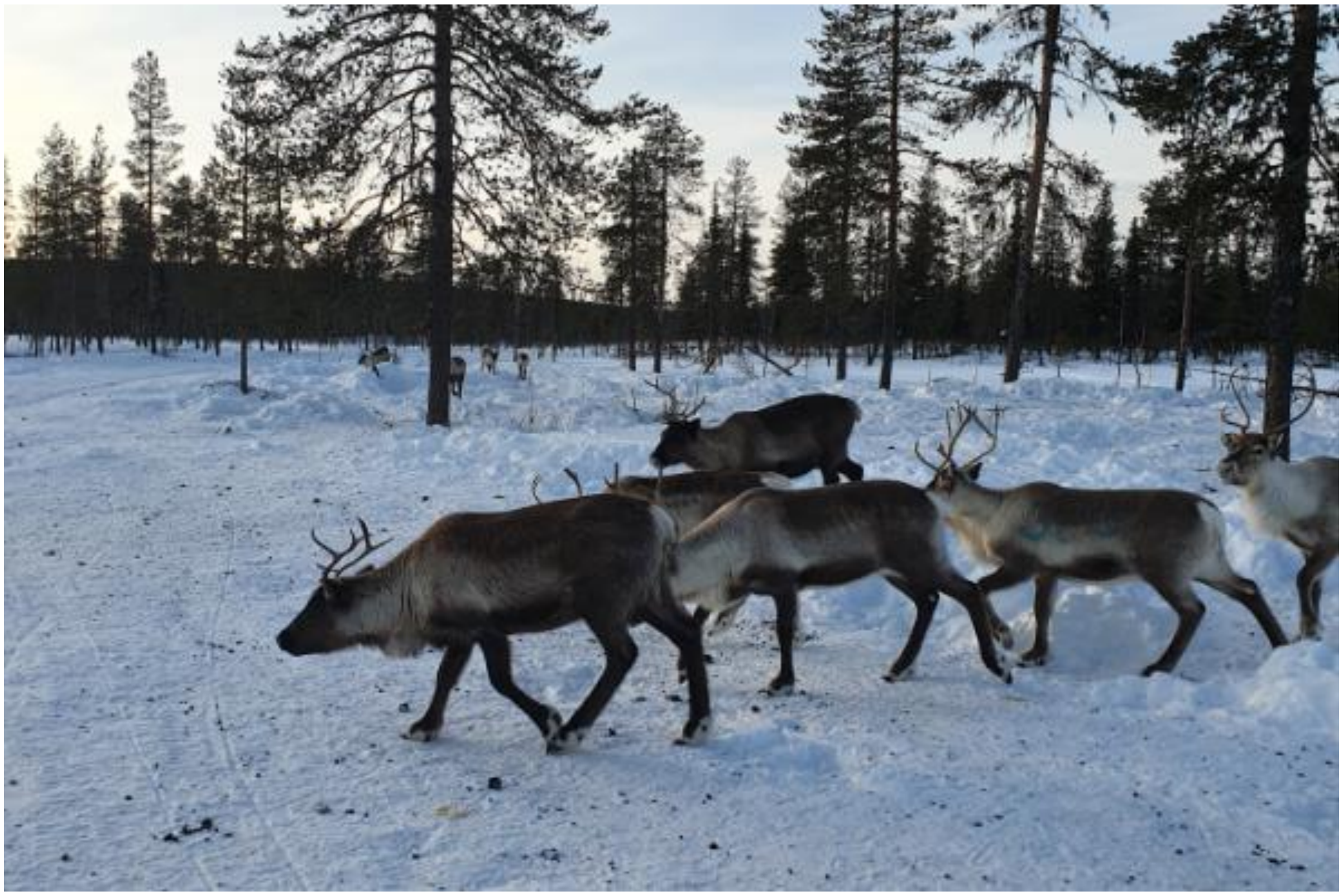

Gažaldagat biebmama birra leat juohkán čuovvovaš osiide:

1. Oppalaš oassi

2. Biebmama ávki

3. Biebmama rutiinnat (guoská 2019/2020 áigái)

4. Eará (guoská dan viđa maŋemus jagiide)

92) * Leat go biebman (heahte- dehe lassibiebman) bohccuidat dálveeatnamiin goassege dan maŋemus 5 jagis? Jus din dálvesiida ii leat rátkkašan máŋga dálvesiiddaide, de vástidat don olle dálvesiidda ovddas. FUOMÁŠ: Dás lea sáhka biebmamis dálvesiiddas gárddis ja/dehe lávda/čoahkke ealu biebmamis mii bistii guhkit go guokte vahkku, muhto ii johtima ja čohkkema vuolde mii ádjánii vuollel guokte vahkku.

- ○ Jua
- ○ In

Denne informasjonen vises kun i forhåndsvisningen

Følgende betingelser må være oppfylt for at spørsmålet skal vises for respondenten:

If the question Har du utfodrat (nöd- eller stödutfodrat) dina renar i din vintergruppen vid något tillfälle under de senaste fem åren? Om samebyn inte är uppdelad i olika vintergrupper svarar du för hela samebyn. Obs: här avses utfodring av vintergrupp (slakt- och livren) i hägn och/eller på fribete i över två veckor, men ej under flytt och samling som pågår under en kortare period än två veckor. contains any of these alternatives

- Jua

BIEBMAN

1. Oppalaš oassi

Dás lea sáhka bohccuidat (njuovvan- ja ealihanbohccuid) biebmamis (heahte-/lassibiebman) dálvesiiddas mii bistii badjel guokte vahkku, gárddis dehe lávda/čoahkke ealu biebmamis, muhto ii johtima ja čohkkema vuolde mii ádjánii unnit go guokte vahkku. Jus dálvesiida ii leat rátkojuvvon sierra siiddaide, de vástidat olles dálvesiidda ovddas.

93) * Merke juohke (dálve)jagi, gos bohccot bibmojuvvo, dehe jus eai bibmojuvvon obanassiige. Merke juohke jagi ovddas, ovtta /eanet sivaid dasa.

	In leat biebman	Biebman gárddis	Lávda/čoahkke ealu biebman	In dieđe
2015/2016	☐	☐	☐	☐
2016/2017	☐	☐	☐	☐
2017/2018	☐	☐	☐	☐
2018/2019	☐	☐	☐	☐
2019/2020	☐	☐	☐	☐
2020/2021	☐	☐	☐	☐

Denne informasjonen vises kun i forhåndsvisningen

Følgende betingelser må være oppfylt for at spørsmålet skal vises for respondenten:

If the question Har du utfodrat (nöd- eller stödutfodrat) dina renar i din vintergruppen vid något tillfälle under de senaste fem åren? Om samebyn inte är uppdelad i olika vintergrupper svarar du för hela samebyn. Obs: här avses utfodring av vintergrupp (slakt- och livren) i hägn och/eller på fribete i över två veckor, men ej under flytt och samling som pågår under en kortare period än två veckor. contains any of these alternatives

- Jua

94) Eará mearkkašumit:

Denne informasjonen vises kun i forhåndsvisningen

Følgende betingelser må være oppfylt for at spørsmålet skal vises for respondenten:

If the question Har du utfodrat (nöd- eller stödutfodrat) dina renar i din vintergruppen vid något tillfälle under de senaste fem åren? Om samebyn inte är uppdelad i olika vintergrupper svarar du för hela samebyn. Obs: här avses utfodring av vintergrupp (slakt- och livren) i hägn och/eller på fribete i över två veckor, men ej under flytt och samling som pågår under en kortare period än två veckor. contains any of these alternatives

- Jua

BIEBMAN

1. Oppalaš oassi

Dás lea sáhka bohccuidat (njuovvan- ja ealihanbohccuid) biebmamis (heahte-/lassibiebman) dálvesiiddas mii bistii badjel guokte vahkku, gárddis dehe lávda/čoahkke ealu biebmamis, muhto ii johtima ja čohkkema vuolde mii ádjánii unnit go guokte vahkku. Jus dálvesiida ii leat rátkojuvvon sierra siiddaide, de vástidat olles dálvesiidda ovddas.

95) Manne bibmet bohccuidat dan maŋemus viđa jagi? Merke buot vejolaš vástádusaid mat gusket ealihan bohccuide ja gávpebohccuide. Jus leat erohusaid dan viđa jagis, de čilge dan áinnas dása mearkkašumiid vuolláí (vuolábealde dás).

	Ealihanbohccot	Gávpe-/njuovvanbohccot
Heajos dálveealáhat (guohtun)	☐	☐
Birgen	☐	☐
Ahtanuššan	☐	☐
Boraspiret	☐	☐
Dávddat	☐	☐
Unnidit cesiumdási (Tsjernobyla lihkuhisvuohta)	☐	☐

	Ealihanbohccot	Gávpe-/njuovvanbohccot
Gilvaleaddji eanangeavaheapmi. Čilge, jus lea vejolaš, dás vulobealde	☐	☐
Eará, čilges mii, dás vulobealde	☐	☐

Denne informasjonen vises kun i forhåndsvisningen

Følgende betingelser må være oppfylt for at spørsmålet skal vises for respondenten:

If the question Har du utfodrat (nöd- eller stödutfodrat) dina renar i din vintergruppen vid något tillfälle under de senaste fem åren? Om samebyn inte är uppdelad i olika vintergrupper svarar du för hela samebyn. Obs: här avses utfodring av vintergrupp (slakt- och livren) i hägn och/eller på fribete i över två veckor, men ej under flytt och samling som pågår under en kortare period än två veckor. contains any of these alternatives

- Jua

96) Mearkkašumit, čális guoská go dat njuovvanbohccuide vai ealihanbohccuide:

Denne informasjonen vises kun i forhåndsvisningen

Følgende betingelser må være oppfylt for at spørsmålet skal vises for respondenten:

If the question Har du utfodrat (nöd- eller stödutfodrat) dina renar i din vintergruppen vid något tillfälle under de senaste fem åren? Om samebyn inte är uppdelad i olika vintergrupper svarar du för hela samebyn. Obs: här avses utfodring av vintergrupp (slakt- och livren) i hägn och/eller på fribete i över två veckor, men ej under flytt och samling som pågår under en kortare period än två veckor. contains any of these alternatives

- Jua

2. Gažaldagat biebmama ávkki birra

97) Leat go mearkkašan láhttejit go bohccot, mat leat jeavddalaččat bibmojuvvon unnimusat guokte vahkku maŋŋálaga, eará ládje go luitojuvvojit luovos ellui?

(Ovdamearkka dihte lodjivuođa ektui, álkit dehe váddáseabbo čohkket. Fuomáš: dás ii leat sáhka láhttemis biebmama vuolde).

	Jua	In	In dieđe
Bohccot mat leat bibmojuvvon gárddis	○	○	○
Bohccot mat leat bibmojuvvon čoahkke/lávda ealus / luovos dievás	○	○	○

Denne informasjonen vises kun i forhåndsvisningen

Følgende betingelser må være oppfylt for at spørsmålet skal vises for respondenten:

If the question Har du utfodrat (nöd- eller stödutfodrat) dina renar i din vintergruppen vid något tillfälle under de senaste fem åren? Om samebyn inte är uppdelad i olika vintergrupper svarar du för hela samebyn. Obs: här avses utfodring av vintergrupp (slakt- och livren) i hägn och/eller på fribete i över två veckor, men ej under flytt och samling som pågår under en kortare period än två veckor. contains any of these alternatives

- Jua

98) Eará mearkkašumit:

Denne informasjonen vises kun i forhåndsvisningen

Følgende betingelser må være oppfylt for at spørsmålet skal vises for respondenten:

If the question Har du utfodrat (nöd- eller stödutfodrat) dina renar i din vintergruppen vid något tillfälle under de senaste fem åren? Om samebyn inte är uppdelad i olika vintergrupper svarar du för hela samebyn. Obs: här avses utfodring av vintergrupp (slakt- och livren) i hägn och/eller på fribete i över två veckor, men ej under flytt och samling som pågår under en kortare period än två veckor. contains any of these alternatives

- Jua

2. Gažaldagat biebmama ávkki birra

99) Leat go mearkkašan váikkuha go misiid biebman dálvet daid njuovvandeddui ja vuoibmái čakčat daid misiid ektui mat eai leat bibmojuvvon?

○ Jua

- In
- In dieđe

Denne informasjonen vises kun i forhåndsvisningen

Følgende betingelser må være oppfylt for at spørsmålet skal vises for respondenten:

If the question Har du utfodrat (nöd- eller stödutfodrat) dina renar i din vintergruppen vid något tillfälle under de senaste fem åren? Om samebyn inte är uppdelad i olika vintergrupper svarar du för hela samebyn. Obs: här avses utfodring av vintergrupp (slakt- och livren) i hägn och/eller på fribete i över två veckor, men ej under flytt och samling som pågår under en kortare period än två veckor. contains any of these alternatives

- Jua

100) Leat go mearkkašan lea go áldduid biebman dálvet váikkuhan dasa man miessái (áldduid lohku main lea miessi) dat lea miessemearkuma vuolde ja/dehe čakčat?

- Jua
- In
- In dieđe

Denne informasjonen vises kun i forhåndsvisningen

Følgende betingelser må være oppfylt for at spørsmålet skal vises for respondenten:

If the question Har du utfodrat (nöd- eller stödutfodrat) dina renar i din vintergruppen vid något tillfälle under de senaste fem åren? Om samebyn inte är uppdelad i olika vintergrupper svarar du för hela samebyn. Obs: här avses utfodring av vintergrupp (slakt- och livren) i hägn och/eller på fribete i över två veckor, men ej under flytt och samling som pågår under en kortare period än två veckor. contains any of these alternatives

- Jua

101) Eará mearkkašumit:

Denne informasjonen vises kun i forhåndsvisningen

Følgende betingelser må være oppfylt for at spørsmålet skal vises for respondenten:

If the question Har du utfodrat (nöd- eller stödutfodrat) dina renar i din vintergruppen vid något tillfälle under de senaste fem åren? Om samebyn inte är uppdelad i olika

vintergrupper svarar du för hela samebyn. Obs: här avses utfodring av vintergrupp (slakt- och livren) i hägn och/eller på fribete i över två veckor, men ej under flytt och samling som pågår under en kortare period än två veckor. contains any of these alternatives

- Jua

2. Gažaldagat biebmama ávkki birra

102) Leat go mearkkašan váikkuha go biebman misiid guohtundábiide (mot vistet, ohcet guohtuma) dálvvi maŋŋil biebmama?

- Jua
- In
- In dieđe

Denne informasjonen vises kun i forhåndsvisningen

Følgende betingelser må være oppfylt for at spørsmålet skal vises for respondenten:

If the question Har du utfodrat (nöd- eller stödutfodrat) dina renar i din vintergruppen vid något tillfälle under de senaste fem åren? Om samebyn inte är uppdelad i olika vintergrupper svarar du för hela samebyn. Obs: här avses utfodring av vintergrupp (slakt- och livren) i hägn och/eller på fribete i över två veckor, men ej under flytt och samling som pågår under en kortare period än två veckor. contains any of these alternatives

- Jua

103) Eará mearkkašumit:

Denne informasjonen vises kun i forhåndsvisningen

Følgende betingelser må være oppfylt for at spørsmålet skal vises for respondenten:

If the question Har du utfodrat (nöd- eller stödutfodrat) dina renar i din vintergruppen vid något tillfälle under de senaste fem åren? Om samebyn inte är uppdelad i olika vintergrupper svarar du för hela samebyn. Obs: här avses utfodring av vintergrupp (slakt- och livren) i hägn och/eller på fribete i över två veckor, men ej under flytt och samling som pågår under en kortare period än två veckor. contains any of these alternatives

- Jua

og

If the question 2019/2020 contains any of these alternatives

- Lávda/čoahkke ealu biebman
- Biebman gárddis

3. Gažaldagat mat gusket din dálvesiidda biebmandábiide, 2019/2020 dálvvi ja/dehe giđa.

Dás lea sáhka bohccuidat (njuovvan- ja ealihanbohccuid) biebmamis (heahte-/lassibiebman) dálvesiiddas mii bistii badjel guokte vahkku, gárddis dehe lávda ealu biebmamis, muhto ii johtima ja čohkkema vuolde mii ádjánii unnit go guokte vahkku. Jus olles dálvesiida ii leat rátkojuvvon sierra siiddaide, de vástidat olles dálvesiidda ovddas. Dáiguin gažaldagaiguin iskat otná biebmanrutiinnaid.

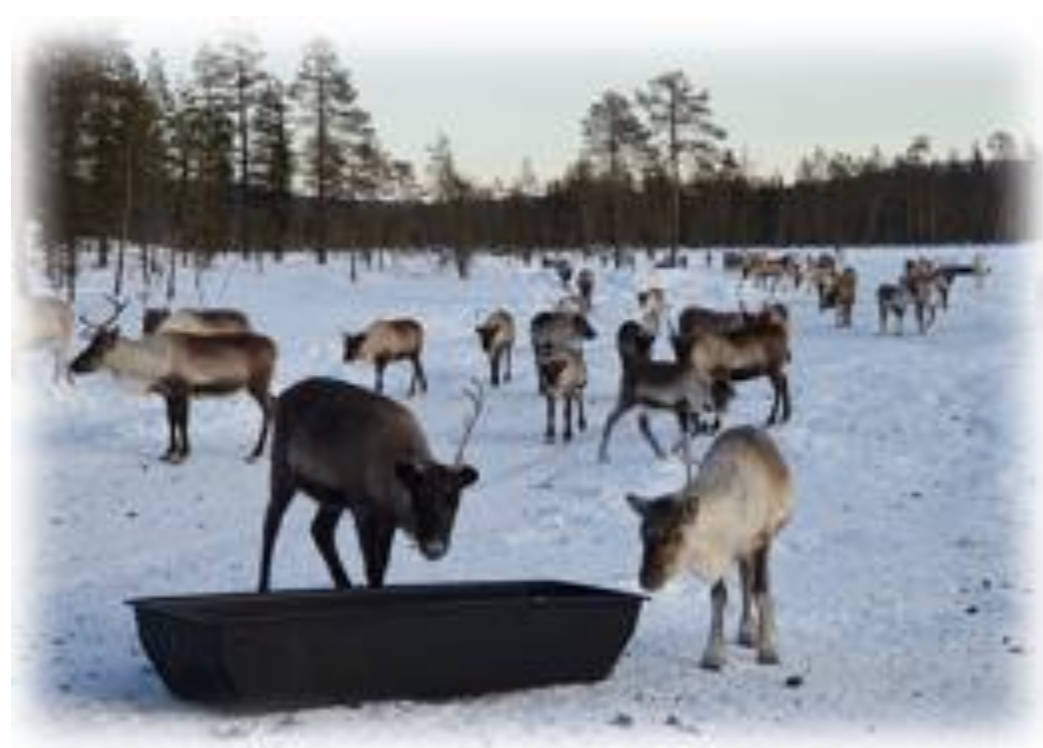

104) Čuovvovaš gažaldagat gusket 2019/2020 boazojahkái. Sullii ollu bohccot bibmojuvvo oktiibuot din dálvesiiddas?

	Gárddis	Čoahkke/lávda ealus/luovos dievás
Vuollel 100	☐	☐
100-299	☐	☐
300-499	☐	☐
500-999	☐	☐
Badjel 1000	☐	☐

Denne informasjonen vises kun i forhåndsvisningen

Følgende betingelser må være oppfylt for at spørsmålet skal vises for respondenten:

If the question Har du utfodrat (nöd- eller stödutfodrat) dina renar i din vintergruppen vid något tillfälle under de senaste fem åren? Om samebyn inte är uppdelad i olika vintergrupper svarar du för hela samebyn. Obs: här avses utfodring av vintergrupp (slakt- och livren) i hägn och/eller på fribete i över två veckor, men ej under flytt och samling som pågår under en kortare period än två veckor. contains any of these alternatives

- Jua

og

If the question 2019/2020 contains any of these alternatives

- Lávda/čoahkke ealu biebman
- Biebman gárddis

105) Makkár čuovvovaš sorttat bohccot bibmojuvvo eanas, ja gos ledje bohccot dalle? Válljie ovtta/eanet molssaeavttuid.

	Gárddis	Čoahkke/lávda ealus/luovos dievás
Olles dálveeallu	☐	☐
Eanas miesit (jahkásaččaid dehe nuorat)	☐	☐
Eanas rávis bohccot (badjel jahkásaččat)	☐	☐

Denne informasjonen vises kun i forhåndsvisningen

Følgende betingelser må være oppfylt for at spørsmålet skal vises for respondenten:

If the question Har du utfodrat (nöd- eller stödutfodrat) dina renar i din vintergruppen vid något tillfälle under de senaste fem åren? Om samebyn inte är uppdelad i olika vintergrupper svarar du för hela samebyn. Obs: här avses utfodring av vintergrupp (slakt- och livren) i hägn och/eller på fribete i över två veckor, men ej under flytt och samling som pågår under en kortare period än två veckor. contains any of these alternatives

- Jua

106) Eará mearkkašumit:

Denne informasjonen vises kun i forhåndsvisningen

Følgende betingelser må være oppfylt for at spørsmålet skal vises for respondenten:

If the question Har du utfodrat (nöd- eller stödutfodrat) dina renar i din vintergruppen vid något tillfälle under de senaste fem åren? Om samebyn inte är uppdelad i olika vintergrupper svarar du för hela samebyn. Obs: här avses utfodring av vintergrupp (slakt- och livren) i hägn och/eller på fribete i över två veckor, men ej under flytt och samling som pågår under en kortare period än två veckor. contains any of these alternatives

- Jua

og

If the question 2019/2020 contains any of these alternatives

- Lávda/čoahkke ealu biebman
- Biebman gárddis

3. Gažaldagat mat gusket din dálvesiidda biebmandábiide, 2019/2020 dálvvi ja/dehe giđa.

107) Sullii man guhká bibmet bohccuid oktilaččat (dan rájes go vuosttaš bohcco biebmagohtet)? Dás čujuhit heahte- dehe lassibiebmamii mii bistii badjel guokte vahku 2019/2020 boazojagi.

	Vuollel mánu	1-3 mánu	Badjel 3 mánu
Gárddis	○	○	○
Čoahkke/lávda ealus/luovos dievás	○	○	○

Denne informasjonen vises kun i forhåndsvisningen

Følgende betingelser må være oppfylt for at spørsmålet skal vises for respondenten:

If the question Har du utfodrat (nöd- eller stödutfodrat) dina renar i din vintergruppen vid något tillfälle under de senaste fem åren? Om samebyn inte är uppdelad i olika vintergrupper svarar du för hela samebyn. Obs: här avses utfodring av vintergrupp (slakt- och livren) i hägn och/eller på fribete i över två veckor, men ej under flytt och samling som pågår under en kortare period än två veckor. contains any of these alternatives

- Jua

og

If the question 2019/2020 contains any of these alternatives

- Lávda/čoahkke ealu biebman
- Biebman gárddis

108) Eará mearkkašumit:

Denne informasjonen vises kun i forhåndsvisningen

Følgende betingelser må være oppfylt for at spørsmålet skal vises for respondenten:

If the question Har du utfodrat (nöd- eller stödutfodrat) dina renar i din vintergruppen vid något tillfälle under de senaste fem åren? Om samebyn inte är uppdelad i olika vintergrupper svarar du för hela samebyn. Obs: här avses utfodring av vintergrupp (slakt- och livren) i hägn och/eller på fribete i över två veckor, men ej under flytt och samling som pågår under en kortare period än två veckor. contains any of these alternatives

- Jua

og

If the question 2019/2020 contains any of these alternatives

- Biebman gárddis

3. Gažaldagat mat gusket din dálvesiidda biebmandábiide, 2019/2020 dálvvi ja/dehe giđa.

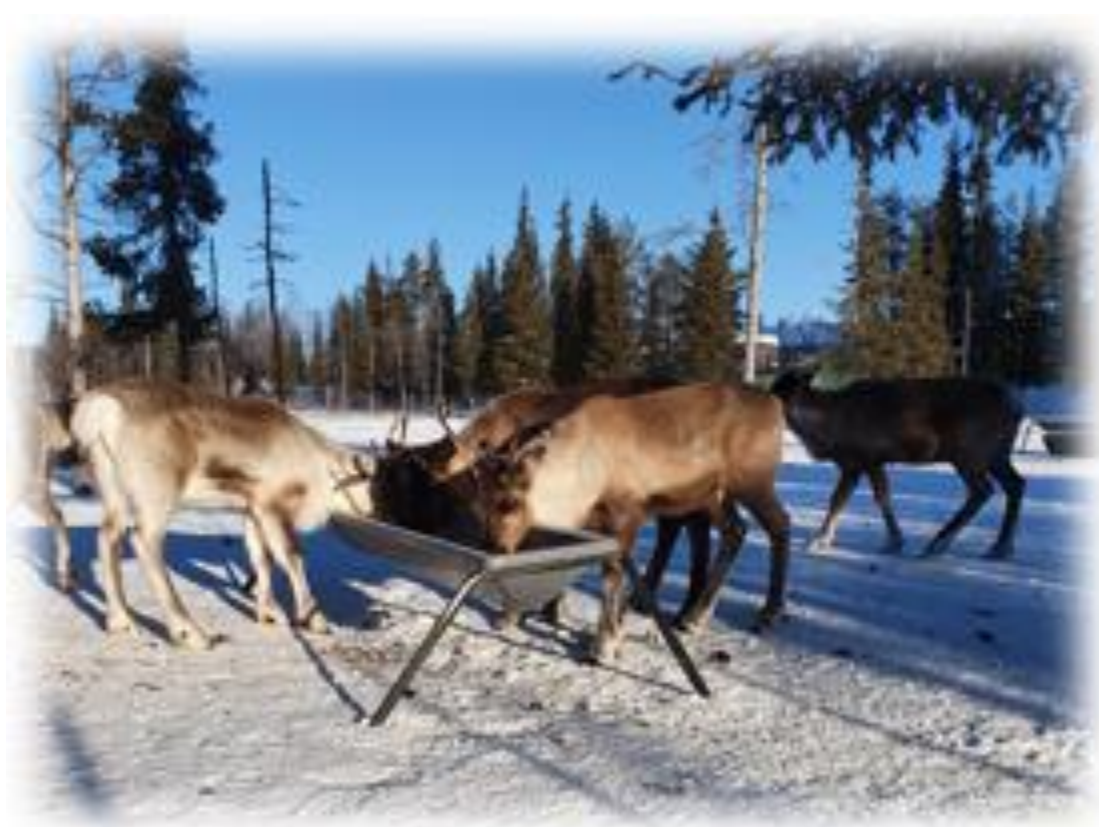

Dáiguin gažaldagaiguin iskat otná biebmanrutiinnaid.

109) * Gažaldagat fuođđariid birra gullet boazodoalu rutiinnaid ja diliid iskamii. Makkár čuovvovaš fuođđariid atnet go bibmet bohccuidat gárddis 2019/2020 boazojagi?

- ○ Dušše suinniiguin (siilosuoinnit/suvra-suoinnit/eará suoidnesorttaiguin)
- ○ Dušše pelletsiiguin

Sihke suinniiguin ja pelletsiiguin

Denne informasjonen vises kun i forhåndsvisningen

Følgende betingelser må være oppfylt for at spørsmålet skal vises for respondenten:

If the question Har du utfodrat (nöd- eller stödutfodrat) dina renar i din vintergruppen vid något tillfälle under de senaste fem åren? Om samebyn inte är uppdelad i olika vintergrupper svarar du för hela samebyn. Obs: här avses utfodring av vintergrupp (slakt- och livren) i hägn och/eller på fribete i över två veckor, men ej under flytt och samling som pågår under en kortare period än två veckor. contains any of these alternatives

- Jua

og

If the question 2019/2020 contains any of these alternatives

- Lávda/čoahkke ealu biebman

110) * Makkár čuovvovaš fuođđariiguin bibmet bohccuidat lávda/čoahkke ealus/luovos dievas 2019/2020 boazojagi?

Duššе suinniiguin (siilosuoinnit/suvra-suoinnit/eará suoidnesorttaiguin)

Duššе pelletsiiguin

Sihke suinniiguin ja pelletsiiguin

Denne informasjonen vises kun i forhåndsvisningen

Følgende betingelser må være oppfylt for at spørsmålet skal vises for respondenten:

If the question Har du utfodrat (nöd- eller stödutfodrat) dina renar i din vintergruppen vid något tillfälle under de senaste fem åren? Om samebyn inte är uppdelad i olika vintergrupper svarar du för hela samebyn. Obs: här avses utfodring av vintergrupp (slakt- och livren) i hägn och/eller på fribete i över två veckor, men ej under flytt och samling som pågår under en kortare period än två veckor. contains any of these alternatives

- Jua

og

If the question 2019/2020 contains any of these alternatives

- Lávda/čoahkke ealu biebman
- Biebman gárddis

111) Eará mearkkašumit:

Denne informasjonen vises kun i forhåndsvisningen

Følgende betingelser må være oppfylt for at spørsmålet skal vises for respondenten:

If the question Har du utfodrat (nöd- eller stödutfodrat) dina renar i din vintergruppen vid något tillfälle under de senaste fem åren? Om samebyn inte är uppdelad i olika vintergrupper svarar du för hela samebyn. Obs: här avses utfodring av vintergrupp (slakt- och livren) i hägn och/eller på fribete i över två veckor, men ej under flytt och samling som pågår under en kortare period än två veckor. contains any of these alternatives

- Jua

og

If the question 2019/2020 contains any of these alternatives

- Lávda/čoahkke ealu biebman
- Biebman gárddis

3. Gažaldagat mat gusket din dálvesiidda biebmandábiide, 2019/2020 dálvvi ja/dehe giđa.

112) Goas bibmet bohccuidat jeahkáliiguin ja/dehe lahpuiguin earret dan dábálaš guohtumii mii lei gávdnamis 2019/2020 boazojagi?

	Gárddis	Čoahkke/lávda ealus/luovos dievás
Lassin fuođđariidda	☐	☐
Skihpa/vuoimmehis bohccuide	☐	☐
Go hárjehin bohccuide fuođđariid	☐	☐
Eai lean jeahkálat/lahput	☐	☐
Eará, čáles dás vulobealde	☐	☐

Denne informasjonen vises kun i forhåndsvisningen

Følgende betingelser må være oppfylt for at spørsmålet skal vises for respondenten:

If the question Har du utfodrat (nöd- eller stödutfodrat) dina renar i din vintergruppen vid något tillfälle under de senaste fem åren? Om samebyn inte är uppdelad i olika vintergrupper svarar du för hela samebyn. Obs: här avses utfodring av vintergrupp (slakt- och livren) i hägn och/eller på fribete i över två veckor, men ej under flytt och

samling som pågår under en kortare period än två veckor. contains any of these alternatives

- Jua

og

If the question 2019/2020 contains any of these alternatives

- Lávda/čoahkke ealu biebman
- Biebman gárddis

og

If the question För att kartlägga rutiner och förutsättningar inom renskötseln följer frågor om olika fodermedel. Vilka av följande fodermedel utfodrade du dina renar med i hägn 2019/2020? contains any of these alternatives

- Sihke suinniiguin ja pelletsiiguin
- Dušše suinniiguin (siilosuoinnit/suvra-suoinnit/eará suoidnesorttaiguin)

og

If the question Vilka av följande fodermedel utfodrade du dina renar med på fribete 2019/2020? contains any of these alternatives

- Sihke suinniiguin ja pelletsiiguin
- Dušše suinniiguin (siilosuoinnit/suvra-suoinnit/eará suoidnesorttaiguin)

113) Jus vejolaš, árvvoštala man ollu roavvafuođđarat (suoinnit), siilosuoidnespáppat, ja makkár šlájat (omd. jorba- dehe njealječiegatspáppat) manne biebmamii olles biebmanáigodagas 2019/2020 boazojagi.

Gárddis

Čoahkke/lávda ealus/luovos dievás

Denne informasjonen vises kun i forhåndsvisningen

Følgende betingelser må være oppfylt for at spørsmålet skal vises for respondenten:

If the question Har du utfodrat (nöd- eller stödutfodrat) dina renar i din vintergruppen vid något tillfälle under de senaste fem åren? Om samebyn inte är uppdelad i olika vintergrupper svarar du för hela samebyn. Obs: här avses utfodring av vintergrupp (slakt- och livren) i hägn och/eller på fribete i över två veckor, men ej under flytt och samling som pågår under en kortare period än två veckor. contains any of these alternatives

- Jua

og

If the question 2019/2020 contains any of these alternatives

- Lávda/čoahkke ealu biebman
- Biebman gárddis

114) Eará mearkkašumit:

Denne informasjonen vises kun i forhåndsvisningen

Følgende betingelser må være oppfylt for at spørsmålet skal vises for respondenten:

If the question Har du utfodrat (nöd- eller stödutfodrat) dina renar i din vintergruppen vid något tillfälle under de senaste fem åren? Om samebyn inte är uppdelad i olika vintergrupper svarar du för hela samebyn. Obs: här avses utfodring av vintergrupp (slakt- och livren) i hägn och/eller på fribete i över två veckor, men ej under flytt och samling som pågår under en kortare period än två veckor. contains any of these alternatives

- Jua

og

If the question 2019/2020 contains any of these alternatives

- Lávda/čoahkke ealu biebman
- Biebman gárddis

og

If the question För att kartlägga rutiner och förutsättningar inom renskötseln följer frågor om olika fodermedel. Vilka av följande fodermedel utfodrade du dina renar med i hägn 2019/2020? contains any of these alternatives

- Sihke suinniiguin ja pelletsiiguin
- Duššе pelletsiiguin

og

If the question Vilka av följande fodermedel utfodrade du dina renar med på fribete 2019/2020? contains any of these alternatives

- Sihke suinniiguin ja pelletsiiguin
- Duššе pelletsiiguin

3. Gažaldagat mat gusket din dálvesiidda biebmandábiide, 2019/2020 dálvvi ja/dehe giđa.

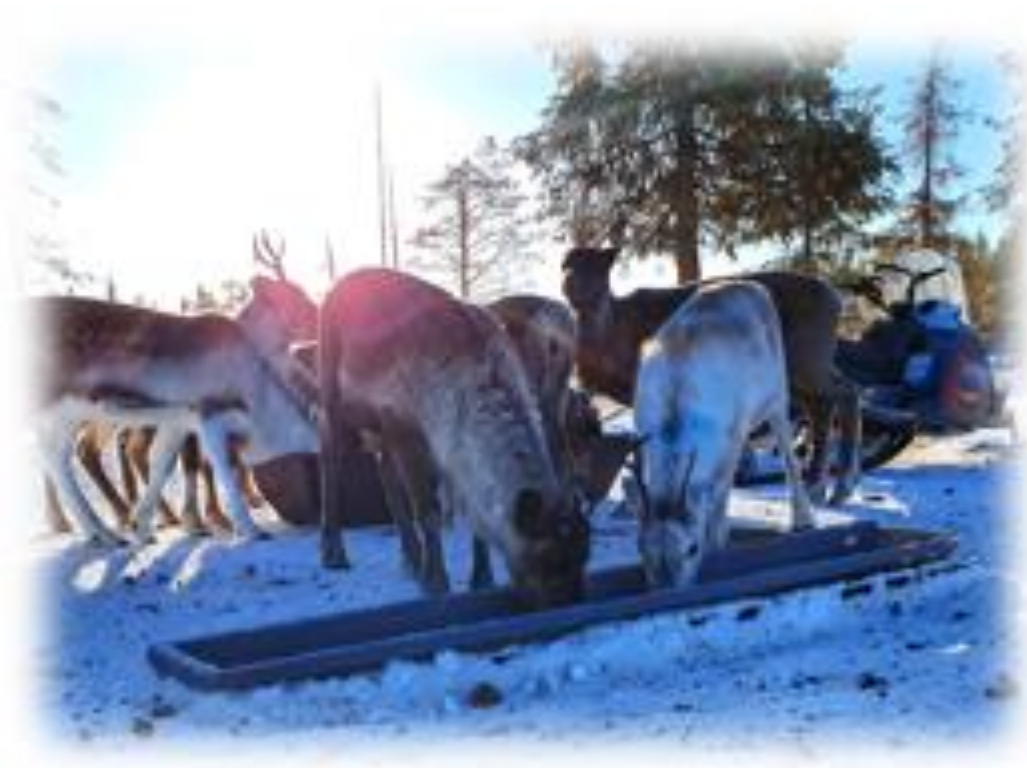

115) Gallii beaivvis bibmet pelletsiiguin (2019/2020)?

	1	2 dehe eanet
Gárddis	○	○
Čoahkke/lávda ealus/luovos dievás	○	○

Denne informasjonen vises kun i forhåndsvisningen

Følgende betingelser må være oppfylt for at spørsmålet skal vises for respondenten:

If the question Har du utfodrat (nöd- eller stödutfodrat) dina renar i din vintergruppen vid något tillfälle under de senaste fem åren? Om samebyn inte är uppdelad i olika vintergrupper svarar du för hela samebyn. Obs: här avses utfodring av vintergrupp (slakt- och livren) i hägn och/eller på fribete i över två veckor, men ej under flytt och samling som pågår under en kortare period än två veckor. contains any of these alternatives

- Jua

og

If the question 2019/2020 contains any of these alternatives

- Lávda/čoahkke ealu biebman
- Biebman gárddis

og

If the question För att kartlägga rutiner och förutsättningar inom renskötseln följer frågor om olika fodermedel. Vilka av följande fodermedel utfodrade du dina renar med i hägn 2019/2020? contains any of these alternatives

- Sihke suinniiguin ja pelletsiiguin
- Duššе pelletsiiguin

og

If the question Vilka av följande fodermedel utfodrade du dina renar med på fribete 2019/2020? contains any of these alternatives

- Sihke suinniiguin ja pelletsiiguin
- Dušše pelletsiiguin

116) Jus vejolaš, árvvoštala man galle gilo manai juohke bohccui juohke beaivái gaskamearalaččat 2019/2020 boazojagi ja čále guoskái go dat heahte- ja/dehe lassibiebmamii.

	Nødfôring	Tilleggsfôring
Gárddis		
Čoahkke/lávda ealus/luovos dievás		

Denne informasjonen vises kun i forhåndsvisningen

Følgende betingelser må være oppfylt for at spørsmålet skal vises for respondenten:

If the question Har du utfodrat (nöd- eller stödutfodrat) dina renar i din vintergruppen vid något tillfälle under de senaste fem åren? Om samebyn inte är uppdelad i olika vintergrupper svarar du för hela samebyn. Obs: här avses utfodring av vintergrupp (slakt- och livren) i hägn och/eller på fribete i över två veckor, men ej under flytt och samling som pågår under en kortare period än två veckor. contains any of these alternatives

- Jua

og

If the question 2019/2020 contains any of these alternatives

- Lávda/čoahkke ealu biebman
- Biebman gárddis

og

If the question För att kartlägga rutiner och förutsättningar inom renskötseln följer frågor om olika fodermedel. Vilka av följande fodermedel utfodrade du dina renar med i hägn 2019/2020? contains any of these alternatives

- Sihke suinniiguin ja pelletsiiguin
- Dušše pelletsiiguin

og

If the question Vilka av följande fodermedel utfodrade du dina renar med på fribete 2019/2020? contains any of these alternatives

- Sihke suinniiguin ja pelletsiiguin
- Dušše pelletsiiguin

117) Eará mearkkašumit:

Denne informasjonen vises kun i forhåndsvisningen

Følgende betingelser må være oppfylt for at spørsmålet skal vises for respondenten:

If the question Har du utfodrat (nöd- eller stödutfodrat) dina renar i din vintergruppen vid något tillfälle under de senaste fem åren? Om samebyn inte är uppdelad i olika vintergrupper svarar du för hela samebyn. Obs: här avses utfodring av vintergrupp (slakt- och livren) i hägn och/eller på fribete i över två veckor, men ej under flytt och samling som pågår under en kortare period än två veckor. contains any of these alternatives

- Jua

og

If the question 2019/2020 contains any of these alternatives

- Lávda/čoahkke ealu biebman
- Biebman gárddis

og

If the question För att kartlägga rutiner och förutsättningar inom renskötseln följer frågor om olika fodermedel. Vilka av följande fodermedel utfodrade du dina renar med i hägn 2019/2020? contains any of these alternatives

- Sihke suinniiguin ja pelletsiiguin
- Duššse pelletsiiguin

og

If the question Vilka av följande fodermedel utfodrade du dina renar med på fribete 2019/2020? contains any of these alternatives

- Sihke suinniiguin ja pelletsiiguin
- Dušše pelletsiiguin

3. Gažaldagat mat gusket din dálvesiidda biebmandábiide, 2019/2020 dálvvi ja/dehe giđa.

118) Jus vejolaš vástidit: Gii lei buvttadan/lágidii/vuvddii pelletsaid 2019/2020 boazojagi:

Gárddis:

Čoahkke/lávda ealus/luovos dievás:

Denne informasjonen vises kun i forhåndsvisningen

Følgende betingelser må være oppfylt for at spørsmålet skal vises for respondenten:

If the question Har du utfodrat (nöd- eller stödutfodrat) dina renar i din vintergruppen vid något tillfälle under de senaste fem åren? Om samebyn inte är uppdelad i olika vintergrupper svarar du för hela samebyn. Obs: här avses utfodring av vintergrupp (slakt- och livren) i hägn och/eller på fribete i över två veckor, men ej under flytt och samling som pågår under en kortare period än två veckor. contains any of these alternatives

- Jua

og

If the question 2019/2020 contains any of these alternatives

- Lávda/čoahkke ealu biebman
- Biebman gárddis

og

If the question För att kartlägga rutiner och förutsättningar inom renskötseln följer frågor om olika fodermedel. Vilka av följande fodermedel utfodrade du dina renar med i hägn 2019/2020? contains any of these alternatives

- Sihke suinniiguin ja pelletsiiguin
- Dušše pelletsiiguin

og

If the question Vilka av följande fodermedel utfodrade du dina renar med på fribete 2019/2020? contains any of these alternatives

- Sihke suinniiguin ja pelletsiiguin
- Dušše pelletsiiguin

119) Mot bibmojuvvo dábálaččat bohccot pelletsiiguin ovddit boazojagi (2019/2020). Merke ovtta/eanet vugiid.

	Gárddis	Čoahkke/lávda ealus/luovos dievás
Botkkuhan njuolga eatnamii	☐	☐
Juolge-biebmangárrái (soimmii)	☐	☐
Juolggehis biebmangárrái (soimmii)	☐	☐
Eará, mii: čális dás vulobealde:	☐	☐

Denne informasjonen vises kun i forhåndsvisningen

Følgende betingelser må være oppfylt for at spørsmålet skal vises for respondenten:

If the question Har du utfodrat (nöd- eller stödutfodrat) dina renar i din vintergruppen vid något tillfälle under de senaste fem åren? Om samebyn inte är uppdelad i olika vintergrupper svarar du för hela samebyn. Obs: här avses utfodring av vintergrupp (slakt- och livren) i hägn och/eller på fribete i över två veckor, men ej under flytt och samling som pågår under en kortare period än två veckor. contains any of these alternatives

- Jua

og

If the question 2019/2020 contains any of these alternatives

- Lávda/čoahkke ealu biebman
- Biebman gárddis

og

If the question För att kartlägga rutiner och förutsättningar inom renskötseln följer frågor om olika fodermedel. Vilka av följande fodermedel utfodrade du dina renar med i hägn 2019/2020? contains any of these alternatives

- Sihke suinniiguin ja pelletsiiguin
- Duššе pelletsiiguin

og

If the question Vilka av följande fodermedel utfodrade du dina renar med på fribete 2019/2020? contains any of these alternatives

- Sihke suinniiguin ja pelletsiiguin
- Duššе pelletsiiguin

120) Eará mearkkašumit:

Denne informasjonen vises kun i forhåndsvisningen

Følgende betingelser må være oppfylt for at spørsmålet skal vises for respondenten:

If the question Har du utfodrat (nöd- eller stödutfodrat) dina renar i din vintergruppen vid något tillfälle under de senaste fem åren? Om samebyn inte är uppdelad i olika vintergrupper svarar du för hela samebyn. Obs: här avses utfodring av vintergrupp

(slakt- och livren) i hägn och/eller på fribete i över två veckor, men ej under flytt och samling som pågår under en kortare period än två veckor. contains any of these alternatives

- Jua

4.Gažaldagat eará šlájat fuođđariid ja biebmanrutiinnaid birra dan maŋemus viđa jagis.

Dás lea sáhka bohccuidat (njuovvan- ja ealihanbohccuid) biebmamis (heahte-/lassibiebman) dálvesiiddas mii bistii badjel guokte vahkku, gárddis dehe lávda ealu biebmamis, muhto ii johtima ja čohkkema vuolde mii ádjánii unnit go guokte vahkku. Jus olles dálvesiida ii leat rátkojuvvon sierra siiddaide, de vástidat olles dálvesiidda ovddas.

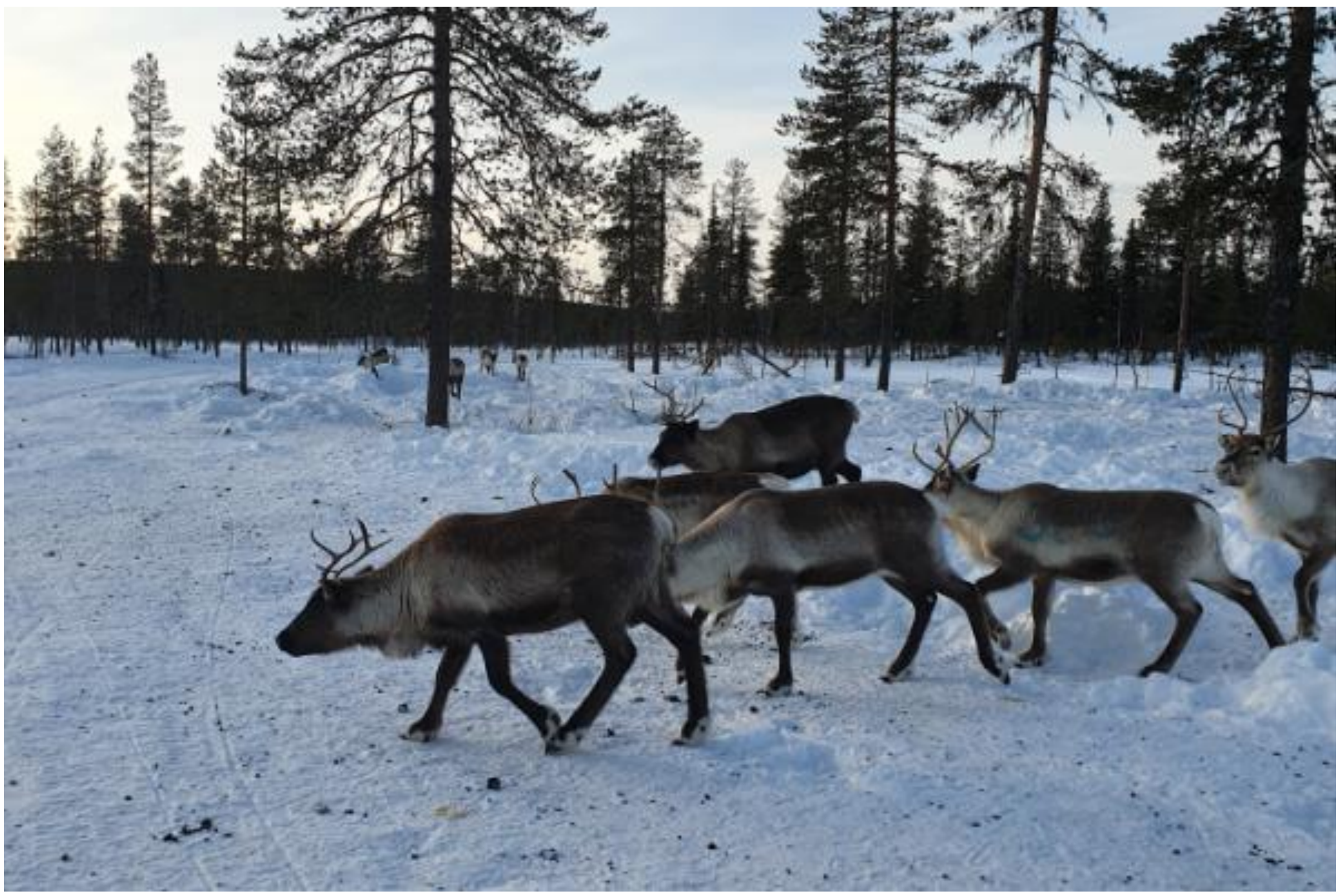

Dáiguin gažaldagaiguin háliidit oažžut dieđuid otná biebmanrutiinnaid birra.

121) Man dábálaš lea leamaš addit bohccuide minerálalasáhusaid (omd. minerálasáltegári, minerálagári, fuođardáiggi, injekšuvnna/náluin) biebmama oktavuođas dán maŋemus viđa jagis? Vállje ovtta/eanet vuogi.

	Gárddis	Čoahkke/lávda ealus/luovos dievás
Álohii biebmama oktavuođas	☐	☐

	Gárddis	Čoahkke/lávda ealus/luovos dievás
Muhtomin biebmama oktavuođas	☐	☐
Muhtun áigodagaid	☐	☐
Ii goassege	☐	☐
Dihto diliid oktavuođas (omd. bohccuide mat leat heajos vuoimmis ja skihpa bohccuide gárddis)	☐	☐
Eará, čilges dás vulobealde:	☐	☐

Denne informasjonen vises kun i forhåndsvisningen

Følgende betingelser må være oppfylt for at spørsmålet skal vises for respondenten:

If the question Har du utfodrat (nöd- eller stödutfodrat) dina renar i din vintergruppen vid något tillfälle under de senaste fem åren? Om samebyn inte är uppdelad i olika vintergrupper svarar du för hela samebyn. Obs: här avses utfodring av vintergrupp (slakt- och livren) i hägn och/eller på fribete i över två veckor, men ej under flytt och samling som pågår under en kortare period än två veckor. contains any of these alternatives

- Jua

122) Eará mearkkašumit:

Denne informasjonen vises kun i forhåndsvisningen

Følgende betingelser må være oppfylt for at spørsmålet skal vises for respondenten:

If the question Har du utfodrat (nöd- eller stödutfodrat) dina renar i din vintergruppen vid något tillfälle under de senaste fem åren? Om samebyn inte är uppdelad i olika vintergrupper svarar du för hela samebyn. Obs: här avses utfodring av vintergrupp (slakt- och livren) i hägn och/eller på fribete i över två veckor, men ej under flytt och samling som pågår under en kortare period än två veckor. contains any of these alternatives

- Jua

123) Jus leat addán minerálaid, čális makkár mearka ja šládja lei das maid don maŋemus addet bohccuide.

Gárddis

Čoahkke/lávda ealus/luovos dievás

Denne informasjonen vises kun i forhåndsvisningen

Følgende betingelser må være oppfylt for at spørsmålet skal vises for respondenten:

If the question Har du utfodrat (nöd- eller stödutfodrat) dina renar i din vintergruppen vid något tillfälle under de senaste fem åren? Om samebyn inte är uppdelad i olika vintergrupper svarar du för hela samebyn. Obs: här avses utfodring av vintergrupp (slakt- och livren) i hägn och/eller på fribete i över två veckor, men ej under flytt och samling som pågår under en kortare period än två veckor. contains any of these alternatives

- Jua

4.Gažaldagat eará šlájat fuođđariid ja biebmanrutiinnaid birra dan maŋemus viđa jagis.

124) Man dábálaš lea addit bohccuide sáltegári go biebmá bohccuid (jurddaš dán maŋemus viđa jagis). Vállje ovtta/eanet vástádusaid.

	Gárddis	Čoahkke/lávda ealus/luovos dievás
Álohii biebmama oktavuođas	☐	☐
Muhtomin biebmama oktavuođas	☐	☐
Muhtun áigodagaid	☐	☐
Ii goassege	☐	☐
Dihto diliid oktavuođas (omd. bohccuide mat leat heajos vuoimmis ja skihpa bohccuide gárddis)	☐	☐
Eará, čilges dás vulobealde:	☐	☐

Denne informasjonen vises kun i forhåndsvisningen

Følgende betingelser må være oppfylt for at spørsmålet skal vises for respondenten:

If the question Har du utfodrat (nöd- eller stödutfodrat) dina renar i din vintergruppen vid något tillfälle under de senaste fem åren? Om samebyn inte är uppdelad i olika vintergrupper svarar du för hela samebyn. Obs: här avses utfodring av vintergrupp (slakt- och livren) i hägn och/eller på fribete i över två veckor, men ej under flytt och samling som pågår under en kortare period än två veckor. contains any of these alternatives

- Jua

125) Leat go addán eará fuođđariid bohccuide dán maŋemus viđa jagis (omd. fuođardáiggi)?

Gárddis

Čoahkke/lávda ealus/luovos dievás

Denne informasjonen vises kun i forhåndsvisningen

Følgende betingelser må være oppfylt for at spørsmålet skal vises for respondenten:

If the question Har du utfodrat (nöd- eller stödutfodrat) dina renar i din vintergruppen vid något tillfälle under de senaste fem åren? Om samebyn inte är uppdelad i olika vintergrupper svarar du för hela samebyn. Obs: här avses utfodring av vintergrupp (slakt- och livren) i hägn och/eller på fribete i över två veckor, men ej under flytt och samling som pågår under en kortare period än två veckor. contains any of these alternatives

- Jua

126) Eará mearkkašumit:

Denne informasjonen vises kun i forhåndsvisningen

Følgende betingelser må være oppfylt for at spørsmålet skal vises for respondenten:

If the question Har du utfodrat (nöd- eller stödutfodrat) dina renar i din vintergruppen vid något tillfälle under de senaste fem åren? Om samebyn inte är uppdelad i olika vintergrupper svarar du för hela samebyn. Obs: här avses utfodring av vintergrupp (slakt- och livren) i hägn och/eller på fribete i över två veckor, men ej under flytt och samling som pågår under en kortare period än två veckor. contains any of these alternatives

- Jua

eller

If the question 2015/2016 contains any of these alternatives

- Biebman gárddis

eller

If the question 2016/2017 contains any of these alternatives

- Biebman gárddis

eller

If the question 2017/2018 contains any of these alternatives

- Biebman gárddis

eller

If the question 2018/2019 contains any of these alternatives

- Biebman gárddis

eller

If the question 2019/2020 contains any of these alternatives

- Biebman gárddis

eller

If the question 2020/2021 contains any of these alternatives

- Biebman gárddis

4.Gažaldagat eará šlájat fuođđariid ja biebmanrutiinnaid birra dan maŋemus viđa jagis.

127) Mot leat čázi addán bohccuide go leat biebman daid dán maŋemus viđa jagiin. Vállje ovtta/eanet vugiid.

☐Muohta gárdde siste
☐Muohta lihtiin
☐Čáhci lihtiin
☐Jogaš
☐Galbma ájačáhci
☐Eará, mii: Čális dása vulobeallái mii

Denne informasjonen vises kun i forhåndsvisningen

Følgende betingelser må være oppfylt for at spørsmålet skal vises for respondenten:

If the question Har du utfodrat (nöd- eller stödutfodrat) dina renar i din vintergruppen vid något tillfälle under de senaste fem åren? Om samebyn inte är uppdelad i olika

vintergrupper svarar du för hela samebyn. Obs: här avses utfodring av vintergrupp (slakt- och livren) i hägn och/eller på fribete i över två veckor, men ej under flytt och samling som pågår under en kortare period än två veckor. contains any of these alternatives

- Jua

eller

If the question 2015/2016 contains any of these alternatives

- Biebman gárddis

eller

If the question 2016/2017 contains any of these alternatives

- Biebman gárddis

eller

If the question 2017/2018 contains any of these alternatives

- Biebman gárddis

eller

If the question 2018/2019 contains any of these alternatives

- Biebman gárddis

eller

If the question 2019/2020 contains any of these alternatives

- Biebman gárddis

128) Eará mearkkašumit:

Denne informasjonen vises kun i forhåndsvisningen

Følgende betingelser må være oppfylt for at spørsmålet skal vises for respondenten:

If the question Har du utfodrat (nöd- eller stödutfodrat) dina renar i din vintergruppen vid något tillfälle under de senaste fem åren? Om samebyn inte är uppdelad i olika vintergrupper svarar du för hela samebyn. Obs: här avses utfodring av vintergrupp (slakt- och livren) i hägn och/eller på fribete i över två veckor, men ej under flytt och samling som pågår under en kortare period än två veckor. contains any of these alternatives

- Jua

4.Gažaldagat eará šlájat fuođđariid ja biebmanrutiinnaid birra dan maŋemus viđa jagis.

129) Leat go vuogit mot hárjehit bohccuid guohtut fuođđariid?

- ○ Jua
- ○ In

Denne informasjonen vises kun i forhåndsvisningen

Følgende betingelser må være oppfylt for at spørsmålet skal vises for respondenten:

If the question Har du utfodrat (nöd- eller stödutfodrat) dina renar i din vintergruppen vid något tillfälle under de senaste fem åren? Om samebyn inte är uppdelad i olika vintergrupper svarar du för hela samebyn. Obs: här avses utfodring av vintergrupp (slakt- och livren) i hägn och/eller på fribete i över två veckor, men ej under flytt och samling som pågår under en kortare period än två veckor. contains any of these alternatives

- Jua

130) Eará mearkkašumit:

Denne informasjonen vises kun i forhåndsvisningen

Følgende betingelser må være oppfylt for at spørsmålet skal vises for respondenten:

If the question Har du utfodrat (nöd- eller stödutfodrat) dina renar i din vintergruppen vid något tillfälle under de senaste fem åren? Om samebyn inte är uppdelad i olika vintergrupper svarar du för hela samebyn. Obs: här avses utfodring av vintergrupp (slakt- och livren) i hägn och/eller på fribete i över två veckor, men ej under flytt och samling som pågår under en kortare period än två veckor. contains any of these alternatives

- Jua

4.Gažaldagat eará šlájat fuođđariid ja biebmanrutiinnaid birra dan maŋemus viđa jagis.

131) * Válddát go dábálaččat eret boares fuođđariid ovdal go attát ođđa/varas fuođđariid bohccuide?

- Jua
- In

Denne informasjonen vises kun i forhåndsvisningen

Følgende betingelser må være oppfylt for at spørsmålet skal vises for respondenten:

If the question Har du utfodrat (nöd- eller stödutfodrat) dina renar i din vintergruppen vid något tillfälle under de senaste fem åren? Om samebyn inte är uppdelad i olika vintergrupper svarar du för hela samebyn. Obs: här avses utfodring av vintergrupp (slakt- och livren) i hägn och/eller på fribete i över två veckor, men ej under flytt och samling som pågår under en kortare period än två veckor. contains any of these alternatives

- Jua

132) Eará mearkkašumit:

Denne informasjonen vises kun i forhåndsvisningen

Følgende betingelser må være oppfylt for at spørsmålet skal vises for respondenten:

If the question Har du utfodrat (nöd- eller stödutfodrat) dina renar i din vintergruppen vid något tillfälle under de senaste fem åren? Om samebyn inte är uppdelad i olika vintergrupper svarar du för hela samebyn. Obs: här avses utfodring av vintergrupp (slakt- och livren) i hägn och/eller på fribete i över två veckor, men ej under flytt och samling som pågår under en kortare period än två veckor. contains any of these alternatives

- Jua

og

If the question Tar du vanligtvis bort överblivet foder innan du utfodrar med nytt? contains any of these alternatives

- Jua

4.Gažaldagat eará šlájat fuođđariid ja biebmanrutiinnaid birra dan maŋemus viđa jagis.

133) Gosa dábálaččat bálkestat/bijat boares fuođđariid?

- Gárdái

- Gárddi olggobeallái
- Eará, gosa

Denne informasjonen vises kun i forhåndsvisningen

Følgende betingelser må være oppfylt for at spørsmålet skal vises for respondenten:

If the question Har du utfodrat (nöd- eller stödutfodrat) dina renar i din vintergruppen vid något tillfälle under de senaste fem åren? Om samebyn inte är uppdelad i olika vintergrupper svarar du för hela samebyn. Obs: här avses utfodring av vintergrupp (slakt- och livren) i hägn och/eller på fribete i över två veckor, men ej under flytt och samling som pågår under en kortare period än två veckor. contains any of these alternatives

- Jua

og

If the question Tar du vanligtvis bort överblivet foder innan du utfodrar med nytt? contains any of these alternatives

- Jua

134) Eará mearkkašumit:

Denne informasjonen vises kun i forhåndsvisningen

Følgende betingelser må være oppfylt for at spørsmålet skal vises for respondenten:

If the question Har du utfodrat (nöd- eller stödutfodrat) dina renar i din vintergruppen vid något tillfälle under de senaste fem åren? Om samebyn inte är uppdelad i olika vintergrupper svarar du för hela samebyn. Obs: här avses utfodring av vintergrupp (slakt- och livren) i hägn och/eller på fribete i över två veckor, men ej under flytt och samling som pågår under en kortare period än två veckor. contains any of these alternatives

- Jua

4.Gažaldagat eará šlájat fuođđariid ja biebmanrutiinnaid birra dan maŋemus viđa jagis.

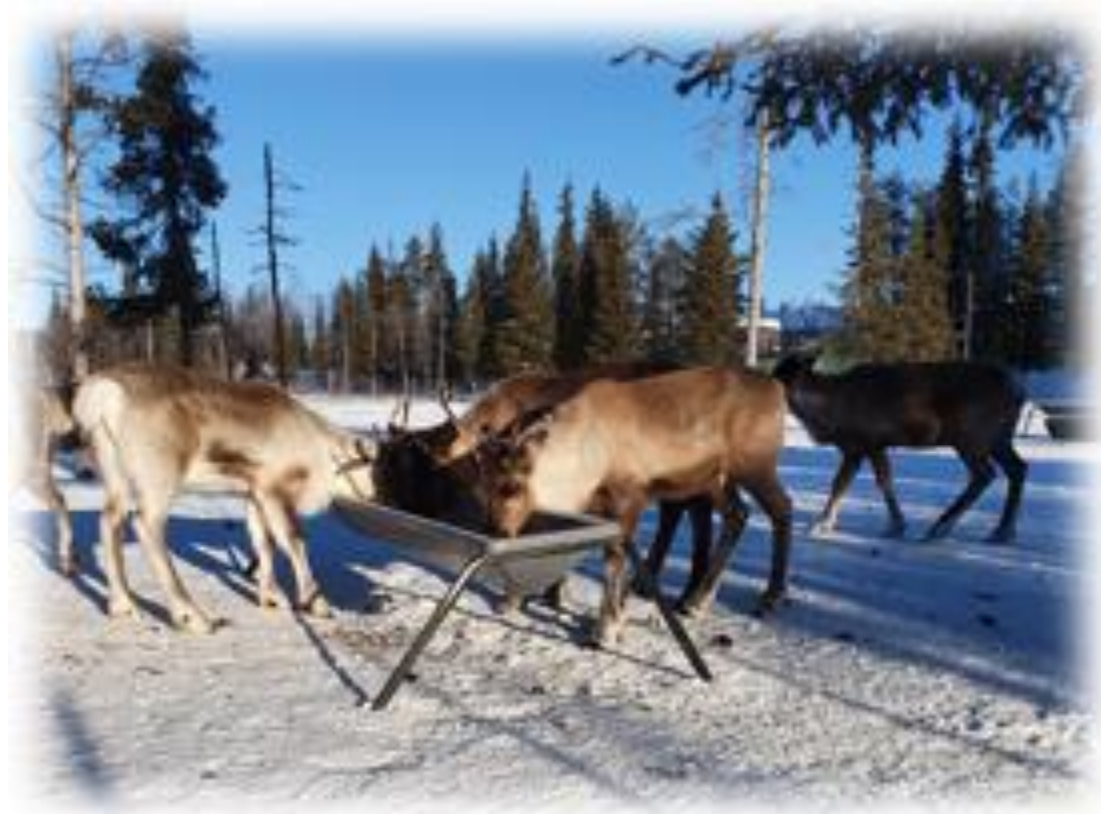

135) Váikkuha go dat, ahte gávdnojit sierranas fuođđarat (siilosuoinnit/suvrrasuoinnit/pellets/ jeahkálat) dasa maiguin vállјet biebmat bohccuidat? Čilge áinnas dan dás vulobealde.

- ○ Jua, mainnalágiin – čilges dás vulobealde
- ○ In

Denne informasjonen vises kun i forhåndsvisningen

Følgende betingelser må være oppfylt for at spørsmålet skal vises for respondenten:

If the question Har du utfodrat (nöd- eller stödutfodrat) dina renar i din vintergruppen vid något tillfälle under de senaste fem åren? Om samebyn inte är uppdelad i olika vintergrupper svarar du för hela samebyn. Obs: här avses utfodring av vintergrupp (slakt- och livren) i hägn och/eller på fribete i över två veckor, men ej under flytt och samling som pågår under en kortare period än två veckor. contains any of these alternatives

- Jua

136) Iskat go roavvafuođđariin (suoidne-) makkár eallámušsisdoallu dain lea ja/dehe man čorgadat/ráidnasat dat leat?

- ○ Jua, eallámušsisdoalu
- ○ Jua, ráinnasvuođa
- ○ Jua, sihke eallámušsisdoalu ja ráinnasvuođa
- ○ In

Denne informasjonen vises kun i forhåndsvisningen

Følgende betingelser må være oppfylt for at spørsmålet skal vises for respondenten:

If the question Har du utfodrat (nöd- eller stödutfodrat) dina renar i din vintergruppen vid något tillfälle under de senaste fem åren? Om samebyn inte är uppdelad i olika vintergrupper svarar du för hela samebyn. Obs: här avses utfodring av vintergrupp

(slakt- och livren) i hägn och/eller på fribete i över två veckor, men ej under flytt och samling som pågår under en kortare period än två veckor. contains any of these alternatives

- Jua

137) Eará mearkkašumit:

Denne informasjonen vises kun i forhåndsvisningen

Følgende betingelser må være oppfylt for at spørsmålet skal vises for respondenten:

If the question Har du utfodrat (nöd- eller stödutfodrat) dina renar i din vintergruppen vid något tillfälle under de senaste fem åren? Om samebyn inte är uppdelad i olika vintergrupper svarar du för hela samebyn. Obs: här avses utfodring av vintergrupp (slakt- och livren) i hägn och/eller på fribete i över två veckor, men ej under flytt och samling som pågår under en kortare period än två veckor. contains any of these alternatives

- Jua

138) Atte áinnas eará dieđuid ja buvtte mearkkašumiid dasa mii guoská bohccuid biebmamii ja/dehe dán guorahallamii dákko:

Denne informasjonen vises kun i forhåndsvisningen

Actions vil skje for følgende alternativer:

Jua, háliidan diehtit eambbo : Redirect to an external URL (https://response.questback.com/statensveterinrmedicinskaansta/erfarenheteravsjukdomigaochmunhosren)

Searvva mielde háhkat eanet dieđuid ja máhtu ja oassálaste ođđaseamos boazodutkamiidda!

Mii ohcat oasseváldiid geat sáhttet searvat vuđoleappot jearahallamiidda mii guoská bohccuid biebmamii. Jearahallama čađaheaba eanas Karin Wallin Philippot Ruoŧas ja Alfa Josteinsdottir Norggas ja mii soahpat searválagaid goas ja gos sáhttá čađahit jearahallama.

139) * Háliidat go searvat háhkat eanet dieđuid ja máhtu ja oassálastit ođđaseamos boazodutkamiidda boazovigiid ja -dávddaid birra?

- In
- Jua, háliidan diehtit eambbo

© Copyright www.questback.com. All Rights Reserved.
Trial Essentials for free - Click here to create your survey today.
